# Pervasive duplication, biased molecular evolution and comprehensive functional analysis of the PP2C family in *Glycine max*

**DOI:** 10.1186/s12864-020-06877-4

**Published:** 2020-07-06

**Authors:** Kai Fan, Yunrui Chen, Zhijun Mao, Yao Fang, Zhaowei Li, Weiwei Lin, Yongqiang Zhang, Jianping Liu, Jinwen Huang, Wenxiong Lin

**Affiliations:** 1 Key Laboratory of Ministry of Education for Genetics, Breeding and Multiple Utilization of Crops, College of Agriculture, Fujian Agriculture and Forestry University, Fuzhou, 350002 P. R. China; 2 Fujian Provincial Key Laboratory of Agroecological Processing and Safety Monitoring, College of Life Sciences, Fujian Agriculture and Forestry University, Fuzhou, 350002 P. R. China; 3 Key Laboratory of Crop Ecology and Molecular Physiology (Fujian Agriculture and Forestry University), Fujian Province University, Fuzhou, 35002 P. R. China

**Keywords:** Protein phosphatase 2C, Soybean, Molecular evolution, Expansion, Functional analysis

## Abstract

**Background:**

Soybean (*Glycine max*) is an important oil provider and ecosystem participant. The protein phosphatase 2C (PP2C) plays important roles in key biological processes. Molecular evolution and functional analysis of the PP2C family in soybean are yet to be reported.

**Results:**

The present study identified 134 GmPP2Cs with 10 subfamilies in soybean. Duplication events were prominent in the GmPP2C family, and all duplicated gene pairs were involved in the segmental duplication events. The legume-common duplication event and soybean-specific tetraploid have primarily led to expanding GmPP2C members in soybean. Sub-functionalization was the main evolutionary fate of duplicated GmPP2C members. Meanwhile, massive genes were lost in the GmPP2C family, especially from the F subfamily. Compared with other genes, the evolutionary rates were slower in the GmPP2C family. The PP2C members from the H subfamily resembled their ancestral genes. In addition, some GmPP2Cs were identified as the putative key regulator that could control plant growth and development.

**Conclusions:**

A total of 134 GmPP2Cs were identified in soybean, and their expansion, molecular evolution and putative functions were comprehensively analyzed. Our findings provided the detailed information on the evolutionary history of the GmPP2C family, and the candidate genes can be used in soybean breeding.

## Background

Reversible protein phosphorylation by protein kinases (PKs) and protein phosphatases (PPs) can regulate numerous biological processes [1]. The PKs can phosphorylate serine (Ser), threonine (Thr), and tyrosine (Tyr), whereas PPs can reverse these reactions by removing the phosphate groups [2]. Based on substrate specificity, the PPs can be classified into three major groups, namely, Ser/Thr phosphatases (STPs), protein Tyr phosphatases (PTPs), and dual-specificity phosphatases (DSPTPs) [2, 3]. PTPs can be further divided into phosphor-protein phosphatases (PPPs) and phosphoprotein metallophosphatases (PPMs) [4]. Many PPs belongs to the PPP family, whereas the PPM family includes protein phosphatase 2C (PP2Cs) and pyruvate dehydrogenase phosphate [2].

The PP2C enzyme is a monomer Mg^2+^/Mn^2+^-dependent PP [2]. The PP2C family has been found in archaea, bacteria, fungi, plants and animals [4]. In plants, the PP2C family is the largest PP family [5, 6]. Over 70 PP2C members have been identified in Arabidopsis [7], rice [8], *Brachypodium distachyon* [9], *Medicago truncatula* [10], maize [11], and cotton [12]. In addition, the PP2C family can regulate plant growth and development, including root development [13], organ initiation [14], stem cell polarity [15], seed dormancy [16], and cell expansion [17]. Moreover, the PP2C members can respond to many biotic and abiotic stresses, including bacterial [18], wound [19], drought [20], salt [21], and cold stresses [22]. PP2C members are very important in hormonal signaling, such as abscisic acid (ABA) [23], jasmonic acid [19] and salicylic acid (SA) [24] pathways.

Soybean (*Glycine max*) is an important oil provider and a natural nitrogen-fixing plant. The release of the soybean genome can help comprehensively characterize the important gene families and analyze their putative functions [25]. Some PP2Cs have been previously identified in soybean. The PP2C-1 can be related to seed weight and size [26], and GmPP2C1 can interact with GmPYL1 in an ABA-dependent manner [27]. GmPP2Cs have already been previously found by searching the NCBI protein database [28], but a comprehensive expansion, molecular evolution and functional analysis of the PP2C family are still lacking in soybean. In this study, a comparative genomic analysis of the PP2C family revealed the species-specific molecular evolution and expansion in soybean. In addition, the expression profiles of the GmPP2C family in growth and development were systematically performed. The gene co-expression network (GCN) and gene regulatory network (GRN) in the GmPP2C members were constructed to unravel certain tissue-specific candidate genes and their putative regulatory mechanisms.

## Results

### Identification and phylogenetic analysis of the PP2C family in soybean, grape, Arabidopsis, *Amborella trichopoda,* and *Nymphaea colorata*

**Fig. 1:**
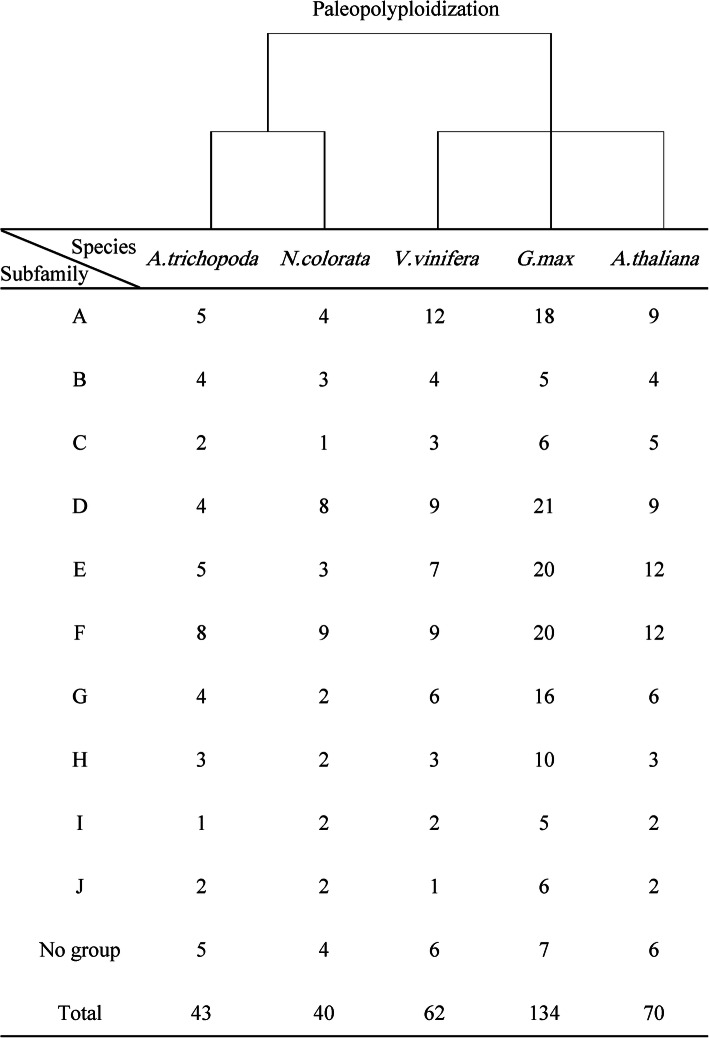
Distribution of the PP2C members in grape, soybean, Arabidopsis, *A. trichopoda*, and *N. colorata*. The upper phylogenetic tree represents the evolutionary relationship of the five species

The Hidden Markov Model (HMM) profile of the PP2C domain (PF00481) was used as the query to identify the PP2C members in soybean, grape, Arabidopsis, and two sequenced basal angiosperms through the HMMER program. After confirming the presence of the PP2C domain using the CDD program, we found 134 GmPP2Cs in soybean, 62 VvPP2Cs in grape, 70 PP2C members in Arabidopsis, 43 AtrPP2Cs in *A. trichopoda*, and 40 AcPP2Cs in *N. colorata* (Fig. 1, Additional files 1, 2, 3, 4, 5, 6).

**Fig. 2:**
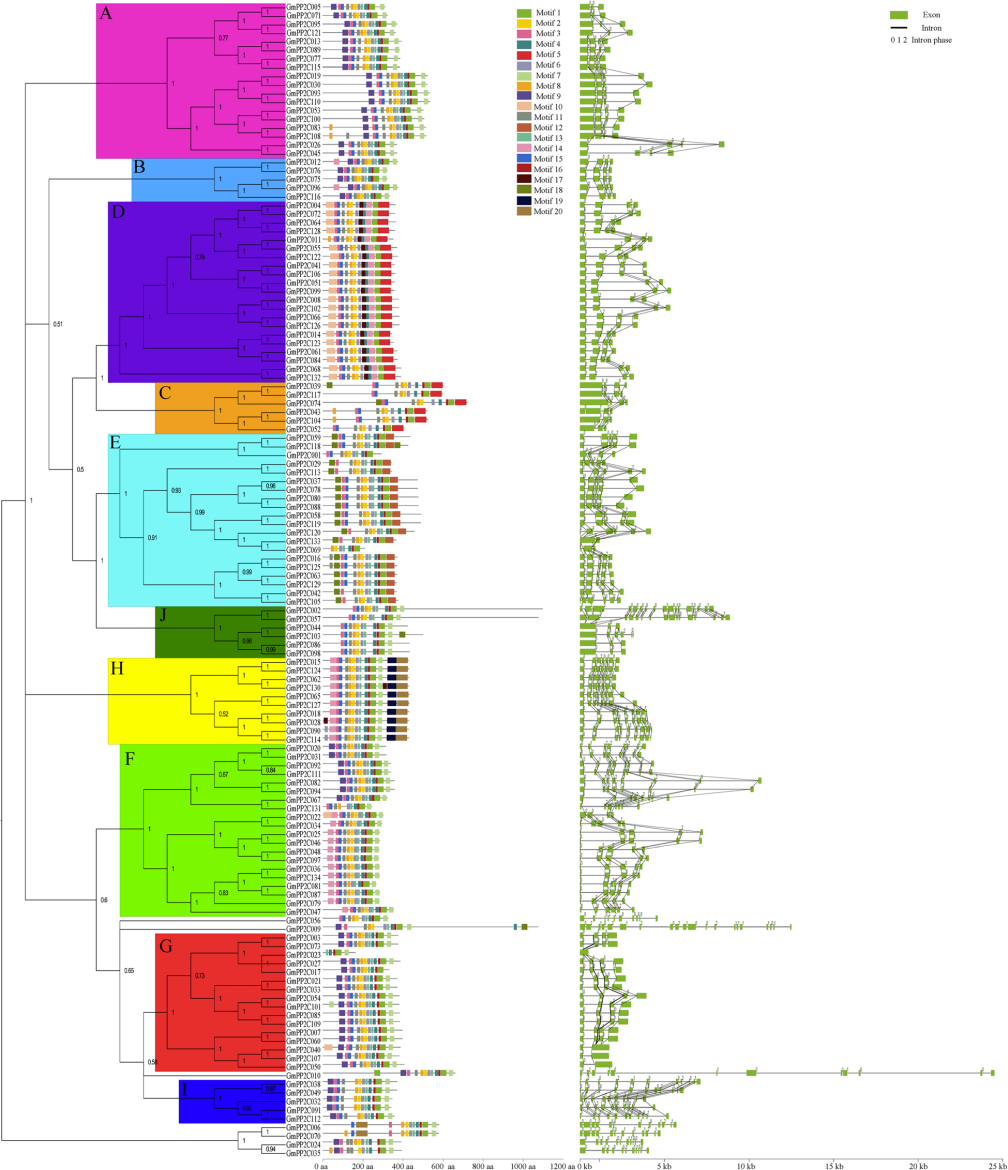
Phylogenetic relationship, putative motif distribution and gene structure of the GmPP2C family. The phylogenetic tree (left) was constructed using the Bayesian method. The numbers were posterior probability values of each clade, and different subfamilies were indicated by different letters and colors. The putative motifs (middle) were identified by the MEME program. Each motif was presented by the specific colored boxes, and the motif location can be estimated using the scale at the bottom. The gene structures (right) were drawn according to the location of exons and introns. The location of exons and introns can be estimated using the scale at the bottom

The phylogenetic analysis of the identified PP2C members was performed using FastTree, Mega, Phylip, and Bayesian (Fig. 2, Additional files 1, 7 and 8). The four different tools constructed the similar phylogenetic trees with high supporting values. The PP2C family was classified into 10 subfamilies, which were designated as A–J subfamilies based on the previous criterion [6]. Each subfamily contained different percentages of PP2C members (Fig. 1 and Additional file 9). In soybean, more than 20 GmPP2Cs were found in the D, E and F subfamilies, whereas only five GmPP2Cs were identified in the B and I subfamilies. The PP2Cs in grape, Arabidopsis, *A. trichopoda*, and *N. colorata* had the similar subfamily distributions. In addition, all of the subfamilies had more PP2C members in soybean than in other plants. The number of PP2C members in the other four eudicots was similar in the B, F, H, I and J subfamilies, but other subfamilies had more PP2Cs in grape and Arabidopsis.

### Conserved motifs and gene structure analysis of the GmPP2C members

All PP2C members had the PP2C domain through the CDD program (Additional files 1 and 10). The locations of the PP2C domain were highly conserved in the PP2C subfamily. Twenty putative motifs were found in the GmPP2C family through the MEME tool (Fig. 2 and Additional file 11). On the basis of the distribution of motifs, the GmPP2C family could be classified into 10 groups, the same classification as the phylogenetic analysis. Eight motifs (motif 1, 2, 3, 4, 5, 6, 7 and 9) were annotated as the PP2C domain through the CDD program. More than 100 GmPP2Cs had 10 motifs (motif 1, 2, 3, 4, 6, 8, 11, 13, 15 and 16), and the other motifs existed in certain specific subfamilies (Additional file 12). Motif 7 existed in all the subfamilies except the C, D and E subfamilies. GmPP2Cs from the A, B, G and I subfamily had motif 9. Moreover, the C subfamily had motif 5, and the D subfamily contained motif 5, 10, 14 and 17. Motif 12 and 18 were enriched in the E subfamily, and the H subfamily had motif 14, 19 and 20. Motif 19 and 20 were only found in the H subfamily. Furthermore, the sequence similarity of GmPP2Cs was analyzed through the Blastp tool (Additional file 13A). GmPP2Cs had a significantly higher sequence similarity in the same subfamily than in the different subfamilies. GmPP2Cs from the H, I and J subfamilies had the highest sequence similarity (~ 600 score), followed by the B, C and D subfamilies (~ 400 score). The F subfamily contained the lowest sequence similarity members (~ 205 score).

Gene structure and intron position were analyzed in the GmPP2C family (Fig. 2 and Additional file 14). Each subfamily had a highly conserved gene structure. Most GmPP2Cs had less than five exons and four introns. However, the highly fragmented members were mainly found in the F, H and I subfamilies. Most GmPP2Cs only contained intron position 0, but GmPP2Cs from the D, E, F, H and I subfamilies had other intron positions.

### Gene duplication and molecular evolution analysis of the PP2C family in soybean

Chromosomal location images of the PP2C family were generated in soybean (Additional files 9, 15 and 16). Each chromosome had GmPP2Cs, but these GmPP2Cs were unevenly distributed across the chromosomes.

**Fig. 3:**
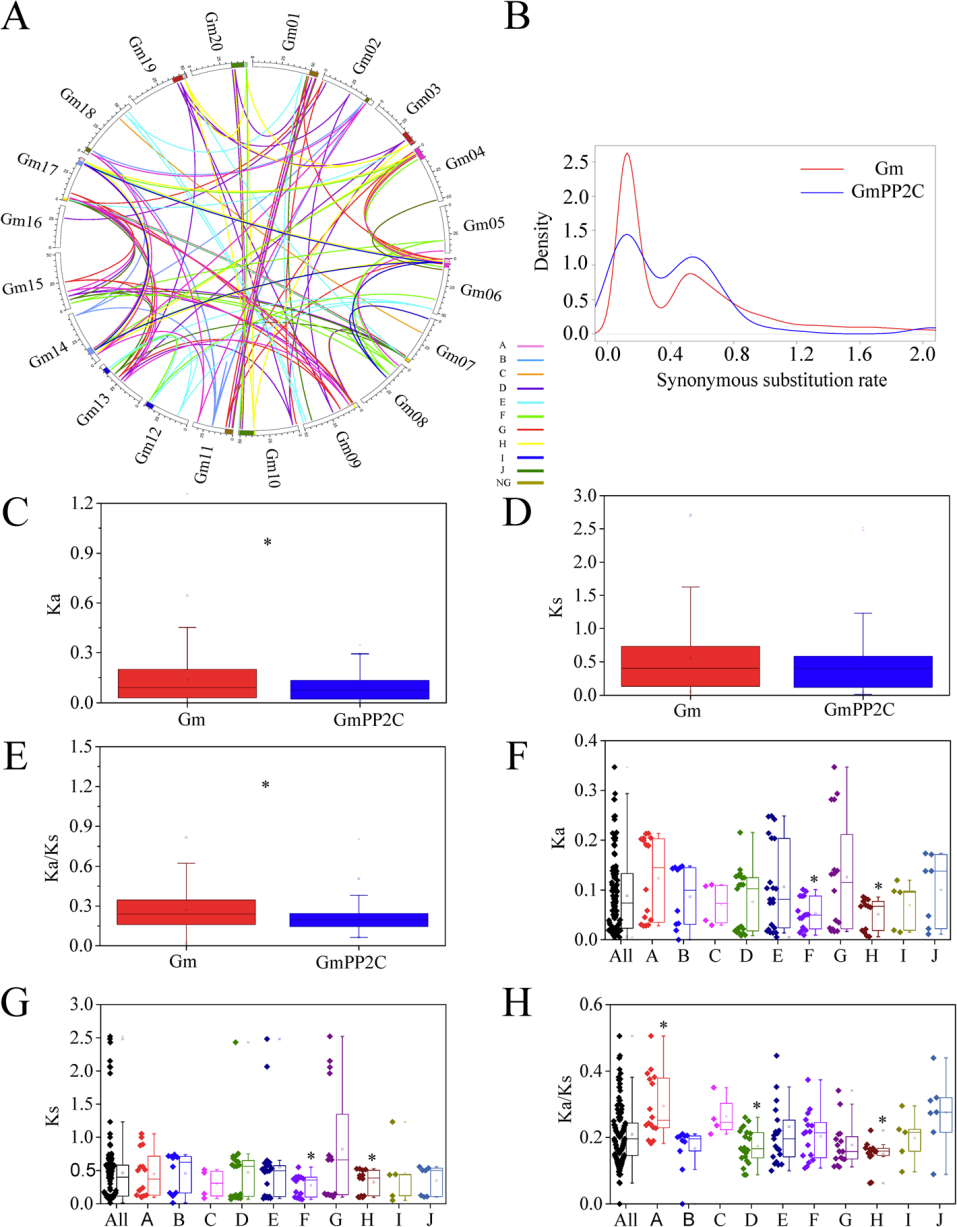
Syntenic and evolutionary analysis of GmPP2Cs. **A** Syntenic analysis of the duplicated GmPP2Cs. GmPP2Cs from the same subfamily were linked by the same colored line. The syntenic blocks, which contained more than three GmPP2Cs, were shown by the color. **B** Distribution of Ks values for all duplicated genes in soybean (red line) and the duplicated GmPP2Cs (blue line). **C-E** Comparison of Ka (**C**), Ks (**D**) and Ka/Ks (**E**) distribution between the duplicated genes and duplicated GmPP2Cs in the soybean. **F-H** Comparison of Ka (**F**), Ks (**G**) and Ka/Ks (**H**) distribution of the duplicated GmPP2Cs in the different subfamilies. Asterisks represent significant differences (*P* < 0.05)

Gene duplication events were investigated to illustrate the expansion pattern of the PP2C family in soybean. A total of 131 duplicated gene pairs were identified in the GmPP2C family (Fig. 3A and Additional file 17), whereas only 13 duplicated gene pairs were found in the VvPP2C family (Additional file 18). Duplication events occurred in each GmPP2C subfamily. The D and E subfamilies had the most duplication events (seven each), followed by the A, F and G subfamilies (more than five each) and the H subfamily (three each). The B and I subfamilies only contained one duplication event. In grape, duplication events were only detected in the A, B, D, F and G subfamilies. Meanwhile, 121 GmPP2Cs were related to duplication events. However, only 13 GmPP2Cs, including five GmPP2Cs from the F subfamily, did not find any duplication event. In addition, 101 syntenic blocks were found in the GmPP2C family (Fig. 3A). The longest syntenic block, including six duplicated gene pairs, was from chromosome 04 and 06, and the syntenic block in chromosome 10 and 20 had five duplicated gene pairs. Furthermore, the sequence similarity of duplicated GmPP2Cs was analyzed through the Blastp tool (Additional file 13B). Duplicated GmPP2Cs from the H subfamilies had the highest sequence similarity (~ 700 score), and the B and F subfamilies contained the lowest sequence similarity members (~ 400 score).

Based on the sequence analysis and chromosomal distribution, all duplicated gene pairs were involved in the segmental duplication events, and no tandem duplication events were found (Additional file 17). Synonymous substitution rate (Ks) value distribution of the GmPP2C duplicated gene pairs was calculated to predict the burst of duplication (Fig. 3B). The Ks values peaked at approximately 0.125 and 0.535, which were similar to the Ks distributions of all soybean duplicated gene pairs. Meanwhile, nonsynonymous substitution rate (Ka) value and Ka/Ks ratio were lower in the GmPP2C duplicated gene pairs than in all soybean duplicated gene pairs (Fig. 3C-E). Moreover, Ka values and Ks values were lower in the F and H subfamilies than in their averages across the entire PP2C family (Fig. 3F-G). The Ka/Ks ratio was less than 1 for the duplicated gene pairs, and it significantly declined in the D and H subfamilies (Fig. 3H). However, Ka values, Ks values, and Ka/Ks ratios of the other subfamilies were similar to one another. Furthermore, polarizing evolutionary rates were found in the GmPP2C subfamily. GmPP2C004/128 and GmPP2C017/073 had higher Ka values than other D and G duplicated gene pairs, and their corresponding sequences also had a lower similarity (Additional file 13B). The Ks values of GmPP2C004/128, GmPP2C016/105, GmPP2C042/125, and GmPP2C003/027 were more than 2, which were different from other gene pairs in the same subfamily. All of the GmPP2C subfamilies, expect for the D and H subfamilies, had a polarizing Ka/Ks ratio.

### Comparative genomic analysis of the PP2C members in soybean and grape

**Fig. 4:**
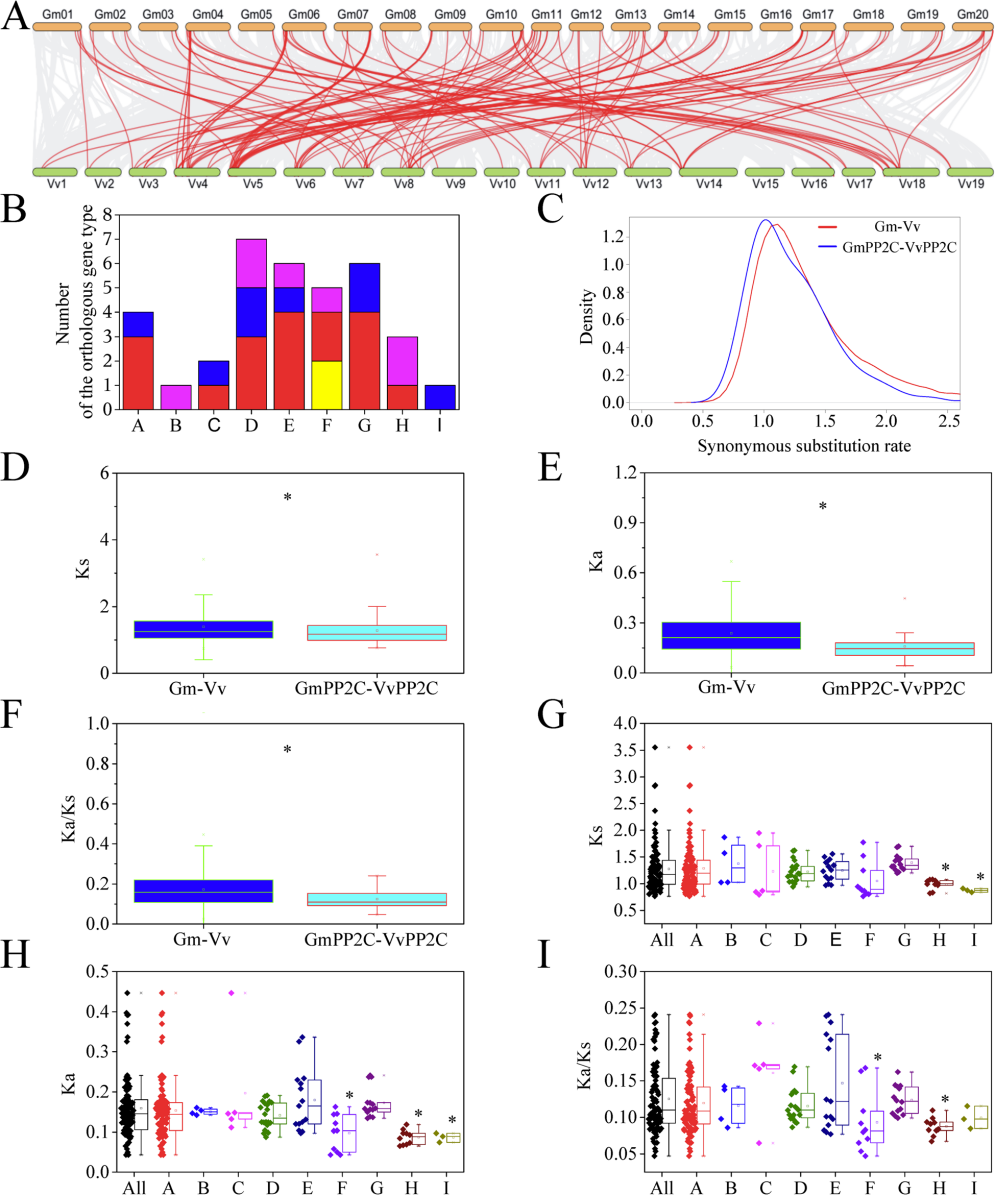
Comparative genomic analysis of the PP2C members in soybean and grape. **A** Microcollinearity patterns between the genomic regions from soybean (top) and grape (down). Gray lines connected orthologous gene pairs, and orthologous PP2Cs were marked with red lines. **B** Comparison of different orthologous gene types in the PP2C subfamily. Yellow: one VvPP2C had one orthologous GmPP2C. Red: one VvPP2C had two orthologous GmPP2Cs. Blue: one VvPP2C had three orthologous GmPP2Cs. Pink: one VvPP2C had four orthologous GmPP2Cs. **C** Distribution of Ks values for all orthologous gene pairs (red line) and PP2Cs (blue line) in soybean and grape. **D-F** Comparison of Ks (**D**), Ka (**E**) and Ka/Ks (**F**) distribution between all of the orthologous gene pairs and PP2Cs in soybean and grape. **G-I** Comparison of Ks (**G**), Ka (**H**) and Ka/Ks (**I**) distribution of the orthologous GmPP2Cs and VvPP2Cs in the different subfamilies. Asterisks represent significant differences (*P* < 0.05)

The grape genome was analyzed to deconvolute the genomic complex in soybean [29, 30]. In this study, 96 orthologous genes of VvPP2Cs were identified in soybean (Fig. 4A and Additional file 19). Twenty VvPP2Cs can find two orthologous genes in soybean (Fig. 4B and Additional file 20). Eight VvPP2Cs with three soybean orthologous genes were found, mostly from the D and G subfamilies. Seven VvPP2Cs, mostly from the D and H subfamilies, had four orthologous genes in soybean. However, two VvPP2Cs from the F subfamily only detected one orthologous gene in soybean.

Ks value distribution was calculated to estimate the divergent time of the PP2C orthologous genes in soybean and grape (Fig. 4C). The Ks values peaked at 1.015, which was significantly lower in the PP2C orthologous genes than in all orthologous genes. Compared with all orthologous genes, Ka values, Ks values, and Ka/Ks ratios significantly decreased in the PP2C orthologous genes (Fig. 4D-F). In addition, the Ks values were lower in the H and I subfamilies than in their averages across the entire PP2C family (Fig. 4G). The Ka values were low in the F, H and I subfamilies, and the Ka/Ks ratios were low in the F and H subfamilies (Fig. 4H-I). However, Ka values, Ks values, and Ka/Ks ratios of the other subfamilies were similar to one another.

### Sequence similarity analysis of the PP2C members in soybean and basal angiosperms

**Fig. 5:**
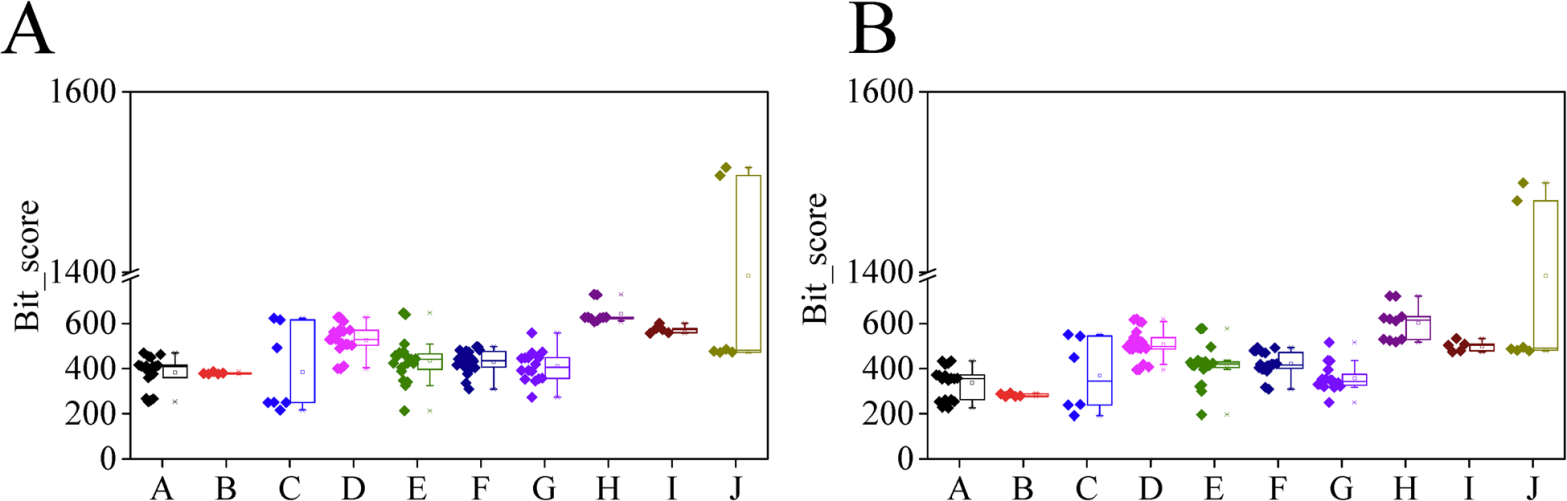
Sequence similarity of the PP2C members in soybean and two basal angiosperms through Blastp program. **A** Sequence similarity of GmPP2Cs and AtrPP2Cs. **B** Sequence similarity of GmPP2Cs and NcPP2Cs

*A. trichopoda* and *N. colorata* are sequenced basal angiosperms. The sequence similarity of PP2Cs in soybean and basal angiosperms was calculated through the Blastp tool (Additional files 21 and 22). The duplicated gene pair (GmPP2C002/057) had the highest sequence similarity to its corresponding orthologous genes in *A. trichopoda* and *N. colorata*. Meanwhile, the highest similarity in soybean and *A. trichopoda* enriched in the PP2C members from the H subfamily, followed by the I subfamily (Fig. 5A). GmPP2Cs and NcPP2Cs from the H subfamily had the highest similarity (Fig. 5B).

### Expression profiles of the GmPP2C members in different tissues

**Fig. 6:**
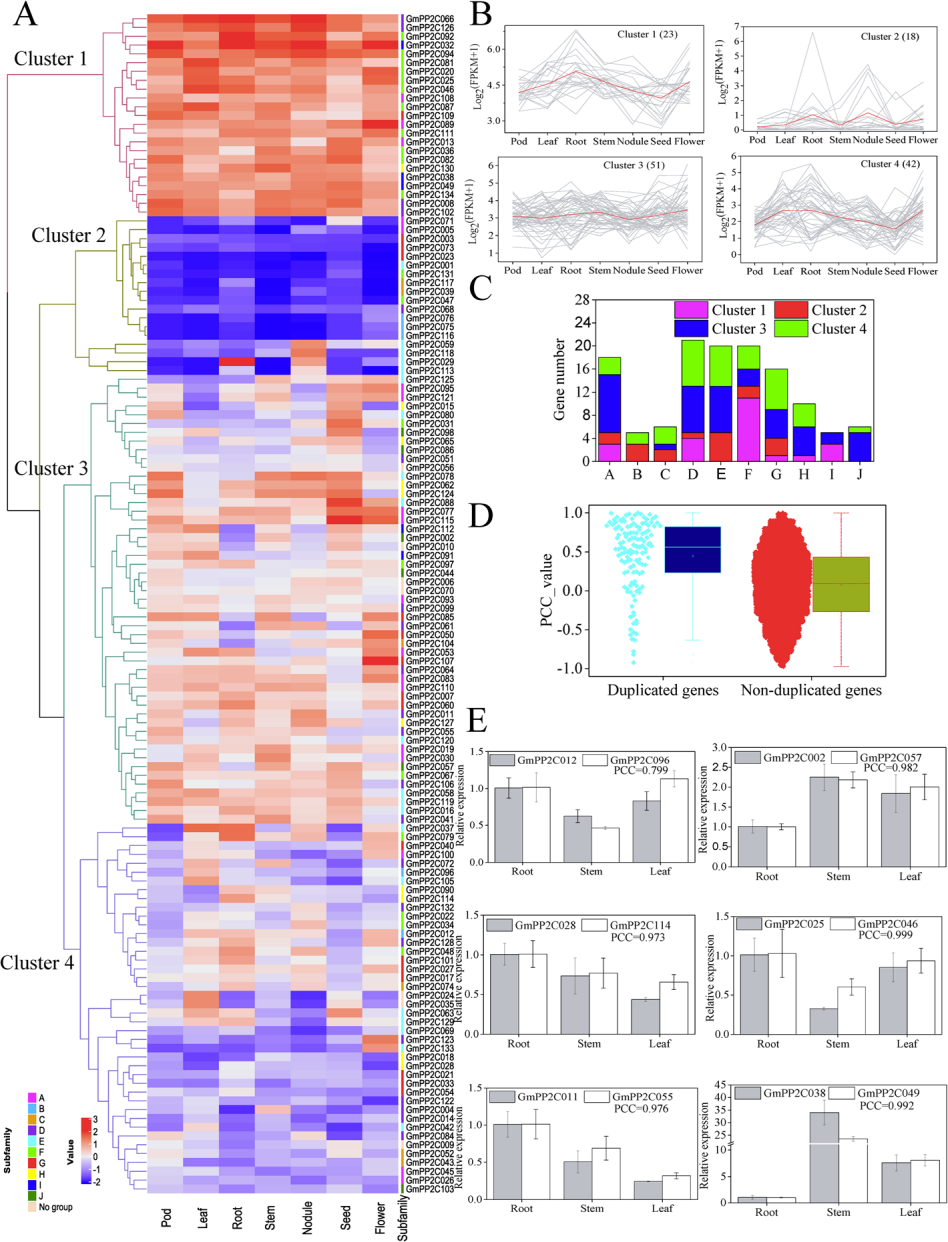
Expression profiles of GmPP2Cs in different tissues. **A** Hierarchical cluster analysis of *GmPP2C* expression in seven tissues. **B** Expression profiles of four clusters in seven tissues. The red lines represented the mean expression level of *GmPP2C*s in the corresponding cluster. **C** Gene numbers of the four clusters in different subfamilies. **D** The PCC distribution of expression levels in the duplicated and non-duplicated GmPP2Cs. **E** Expression of the duplicated *GmPP2C*s in three tissues

The RNA-seq data were analyzed to investigate the expression profiles of the GmPP2C members in different tissues (Fig. 6A and Additional file 23). Data were available for all GmPP2Cs. A total of 117 GmPP2Cs were expressed at medium and high levels (Fragments per Kilobase Million [FPKM] > 5) in one or more tissues. Based on the hierarchical clustering of the expression profiles, GmPP2Cs can be divided into four clusters (Fig. 6A-B). In cluster 1, 23 GmPP2Cs were highly expressed in almost each tissue, whereas 18 GmPP2Cs in cluster 2 had the low expression levels. Cluster 3 included 51 GmPP2Cs with the similar expression level in all tissues. Cluster 4 had 42 GmPP2Cs which were relatively highly expressed in the leaves and roots. In addition, most GmPP2Cs from the same subfamily had the similar expression level (Fig. 6C). Eleven GmPP2Cs from the F subfamily belonged to the cluster 1, and the cluster 2 mainly contained GmPP2Cs from the B, E and G subfamilies. More than half of the GmPP2Cs in cluster 3 came from the A, D and E subfamilies. The GmPP2Cs from the D, E and G subfamilies comprised most of the members in cluster 4. Furthermore, most of the duplicated genes showed the similar expression level in different tissues (Fig. 6A). A total of 102 duplicated gene pairs gathered the same expression clusters. The Pearson Correlation Coefficient (PCC) between GmPP2Cs was also calculated on the basis of the FPKM values (Fig. 6D and Additional file 24). The PCC between non-duplicated GmPP2Cs was a pear-shape distribution, whereas the expression levels of most duplicated GmPP2Cs (73 gene pairs) were highly positively related (PCC > 0.5). Then, six duplicated gene pairs with high PCC and sequence similarity were performed through qRT–PCR analysis (Fig. 6E). The PCCs were more than 0.799 in the expression level of six duplicated gene pairs.

### Gene co-expression network analysis of the GmPP2C members

**Fig. 7:**
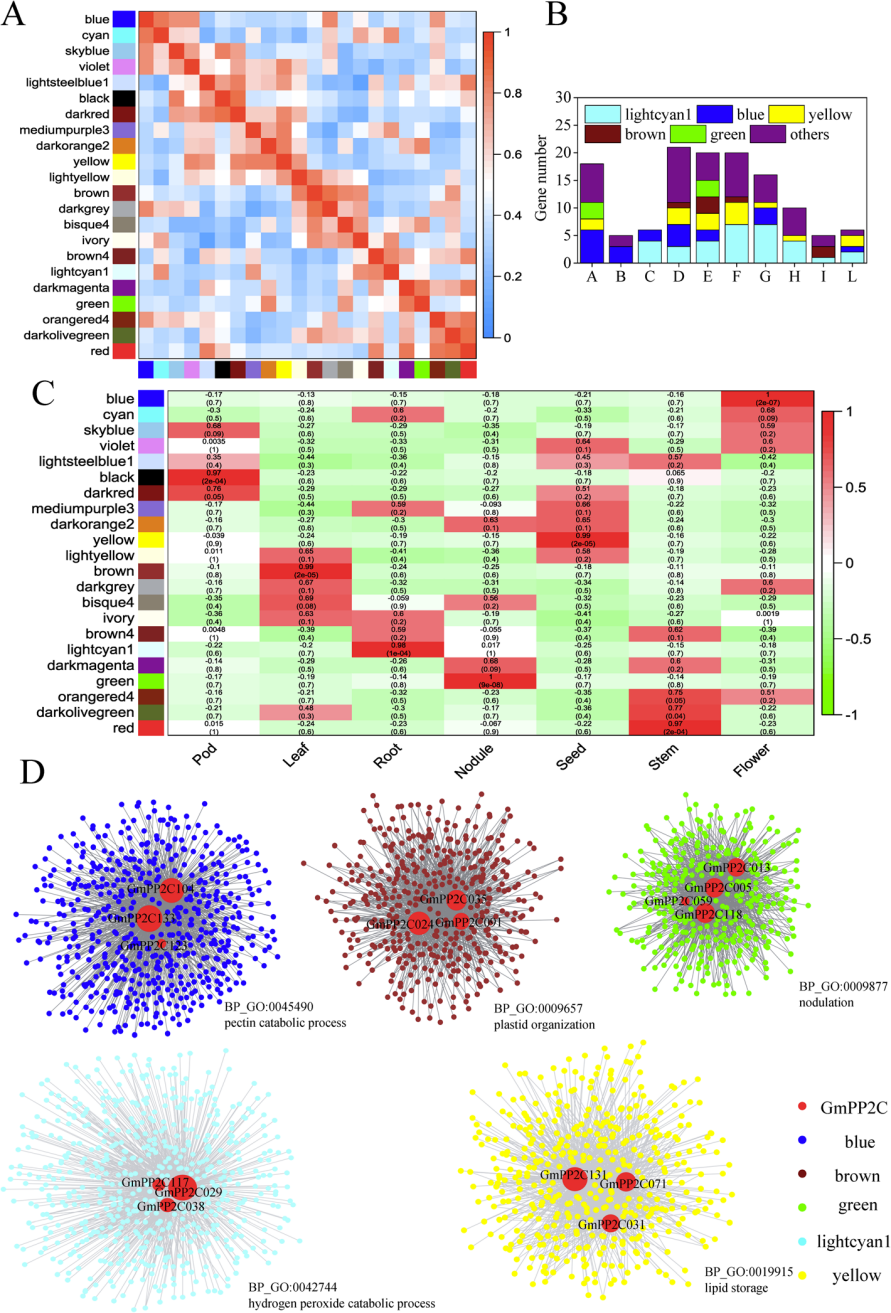
Weighted gene co-expression network analysis of soybean genes in different tissues. **A** Adjacency heatmap of eigengenes in the module. The colored box on the left and bottom side of the heatmap represented the corresponding module. **B** Gene number of different modules in GmPP2C subfamilies. **C** Module-trait heatmap of soybean genes. Columns represented the different tissues and the rows corresponded to module eigengenes. Each cell contained the correlation coefficient and its *P*-values. **D** GCNs using several GmPP2Cs as guide genes. Larger node size indicated greater connectivity within the network. The most enriched GO term of co-expressed genes was marked in the bottom right of the module

Through GCN analysis, 13,954 genes were grouped into 22 modules ranging from 234 genes (bisque4) to 610 genes (ivory) (Additional file 25). Certain modules had a high-positive correlation, such as blue and cyan, and brown and darkgray (Fig. 7A).

Most GmPP2C members were grouped into five major modules (blue, brown, green, lightcyan1 and yellow) (Fig. 7B and Additional file 26). A total of 14 GmPP2Cs from the F and G subfamilies were clustered in lightcyan1, and blue had six members from the A subfamily. Ten GmPP2Cs from the D, E and F subfamilies were clustered in yellow. Meanwhile, genes in module were closely associated with traits (Fig. 7C and Additional file 27). The correlation was 1 between blue and flower, and green and nodule. A high correlation was observed between yellow and seed, brown and leaf, and lightcyan1 and leaf.

Five subnetworks with GmPP2Cs were extracted in the major modules (Fig. 7D). In blue, GmPP2C133 and GmPP2C104 had the most co-expressed genes enriched in gene ontology (GO):0045490 (pectin catabolic process). GmPP2C024, GmPP2C035 and GmPP2C091 were hub genes in brown, and their co-expressed genes were related to plastid organization (GO:0009657). In green, GmPP2C005, GmPP2C013, and GmPP2C118 were mostly connected with many genes with nodulation (GO:0009877). In lightcyan1, a hydrogen peroxide catabolic process was enriched in the co-expressed genes with GmPP2C029 and GmPP2C038. In yellow, many genes had a co-expressed relationship with GmPP2C031, GmPP2C071, and GmPP2C131, and were associated with lipid storage (GO:0019915).

### *cis*-regulatory element analysis

**Fig. 8:**
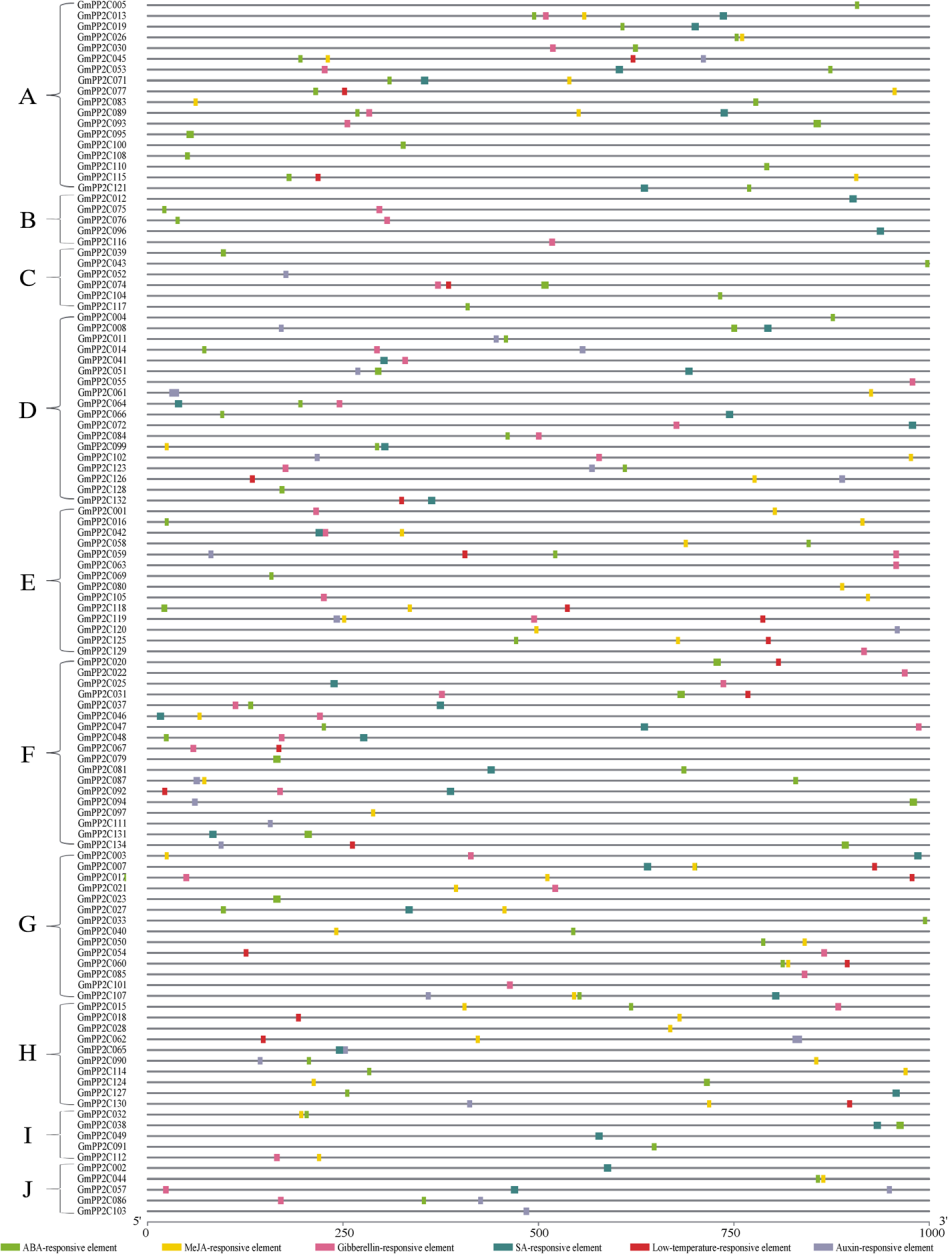
Distributions of certain responsive-regulatory elements in the promoter regions of the GmPP2C members. The PP2C subfamilies in soybean were marked by the brackets. The colored box represented the responsive regulatory elements

The *cis*-regulatory elements are important in the biological functions and regulatory networks. In this study, the promoter regions of the *GmPP2C* members included six *cis*-regulatory elements (Fig. 8 and Additional file 28). All *GmPP2C*s contained at least one *cis*-regulatory element. All *GmPP2C*s from the A subfamily had the ABA-responsive element in their promoters. ABA-, Gibberellin-, and SA-responsive elements were found in many *GmPP2C* promoters from the D and F subfamilies. More than half of the Methyl Jasmonate (MeJA)-responsive elements existed in the *GmPP2C* promoters from the E, G and H subfamilies. Most of the promoter regions of the duplicated gene pairs contained the similar *cis*-regulatory elements in the GmPP2C family.

### Gene regulatory network analysis of the GmPP2C members

**Fig. 9:**
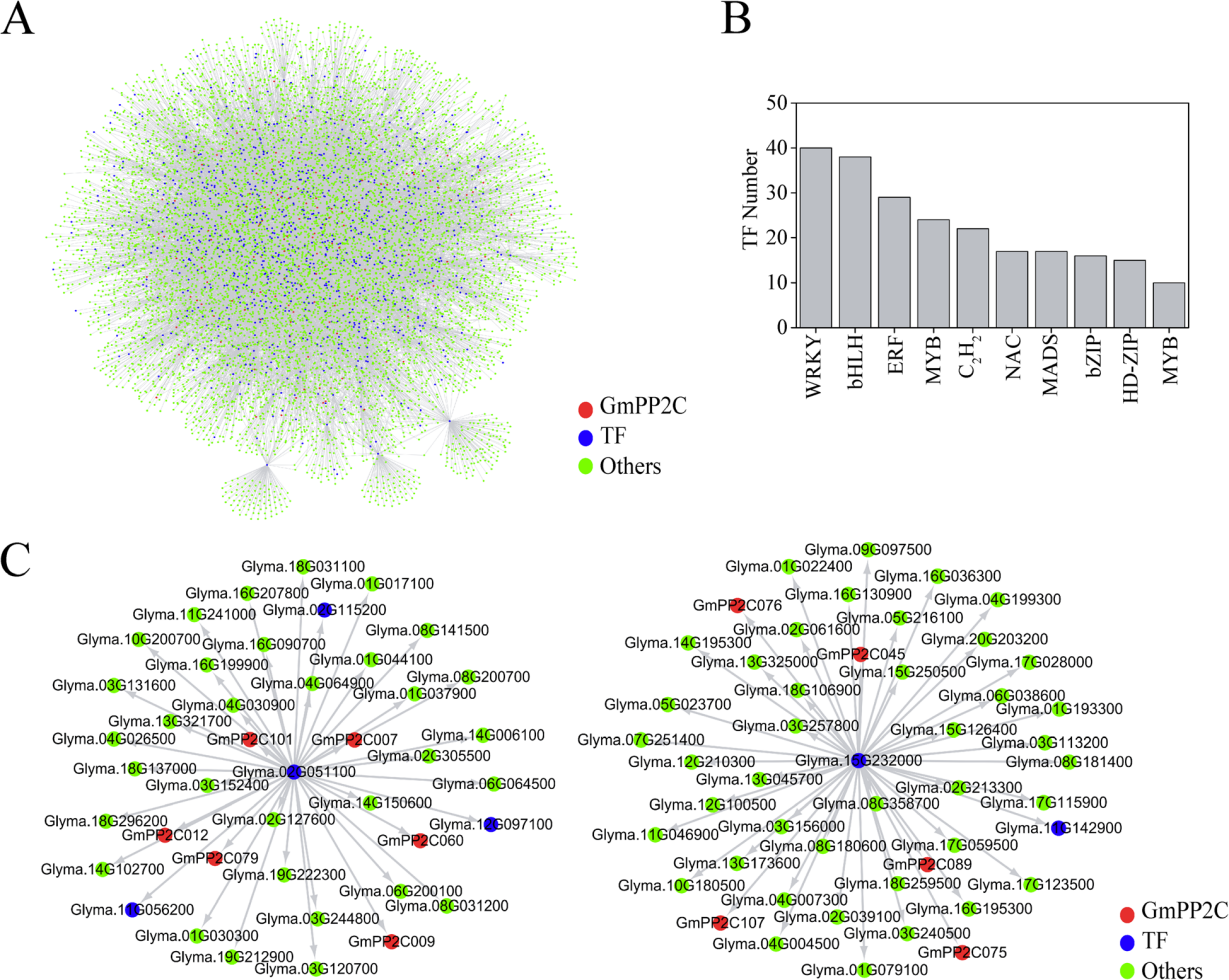
Gene regulatory network analysis of the GmPP2C members by the MERLIN+Prior method. **a** The GRN analysis of the GmPP2C members and its related genes. TFs and GmPP2Cs were marked as blue and red circles, respectively. Green circles represented other genes. **b** The number of TFs which can regulate GmPP2Cs in different subfamilies. **c** The GRN analysis of the most connected TFs in the regulatory GmPP2Cs

The GRN of the GmPP2C members was performed through the MERLIN+Prior method (Fig. 9a). The GmPP2C members can be regulated by 324 transcription factors (TFs) (Additional file 29). Forty and thirty-eight TFs belonged to the WRKY and bHLH families, respectively (Fig. 9b). More than 20 TFs belonged to the ERF, MYB and C_2_H_2_ families. Meanwhile, six and five GmPP2Cs were target genes of Glyma.02G051100 and Glyma.15G232000, respectively (Fig. 9c). Twenty-four target genes of Glyma.02G051100 (~ 59%) belonged to the lightcyan1 module, and 37 target genes of Glyma.15G232000 (~ 79%) belonged to the blue module. In addition, 14 duplicated gene pairs can be regulated by the same TF in the GmPP2C family (Additional file 30). Four duplicated gene pairs can be regulated by the same TFs in the A and H subfamilies, respectively. The related target genes contained half of the GmPP2Cs (five members) in the H subfamily.

## Discussion

In the present study, 134 GmPP2Cs were identified in soybean, whereas less than 70 members were found in grape, Arabidopsis, and two basal angiosperms (Fig. 1, Additional files 1, 2, 3, 4, 5, 6). The increased GmPP2Cs were due to the larger genome (1150 Mb) in soybean [25, 30]. The GmPP2C family could be phylogenetically classified into 10 subfamilies (Figs. 1 and 2, Additional files 1, 7 and 8). The D, E, and F subfamilies had the most GmPP2Cs, whereas the B and I subfamilies had the least members (Fig. 1 and Additional file 9). The greater number was associated with the higher number of GmPP2Cs in the D, E, and F subfamilies. The PP2C members had the similar distribution in grape, Arabidopsis, and two basal angiosperms. Similar distribution of the PP2C family has been reported in Arabidopsis, *M. truncatula* and cotton [7, 10, 12]. Thus, the PP2C family had the conserved subfamily distribution in plants. Meanwhile, the duplicated gene pairs of the PP2C members in soybean (131) were 10 times more than those in grape (13) (Fig. 3A and Additional files 17 and 18). A greater number of the duplicated gene pairs could result in the increased number of the PP2C family (Fig. 1). The duplication events of the GmPP2C family could be found in each subfamily, but were unevenly distributed across the GmPP2C subfamilies. Most duplication events in the GmPP2C family were identified in the A, D, E, F and G subfamilies, whereas each subfamily in grape contained one duplication event at most. The duplication events in the A, D, E, F and G subfamilies led to the expansion of the GmPP2C members.

All duplication events were segmental duplication events in the GmPP2C family (Fig. 3A and Additional files 15 and 17). No tandem duplication events in the GmPP2C family might be related to the genomic fractionation from transposon activities, duplicating and relocating individual genes [31–34]. Moreover, all of the duplicated genes were located in the 101 syntenic blocks (Fig. 3A). The GmPP2C expansion was primarily caused by whole genome duplications [25]. The occurrence of the GmPP2C duplicated gene pairs corresponded to the legume-common duplication event and soybean-specific tetraploid through the Ks analysis (Fig. 3B), and the duplication events occurred simultaneously in GmPP2Cs and other genes (Fig. 3D). Our Ks values were a little lower than those in the previous reports because of the absence evolutionary rate correction [30]. Meanwhile, in our study, detecting the Ks peak of the core eudicot-common hexaploidy was difficult. No identification of the corresponding peak might be due to the Ks peak decline with increasing Ks values or the widespread gene losses after recursive polyploidizations [30, 35].

Based on the Ka/Ks ratio of the duplicated genes, the GmPP2C members mainly have experienced purifying selection with limited functional divergence after whole-genome duplications (Fig. 3E). The similar function in the duplicated genes was further supported by their similar expression profiles (Fig. 6 and Additional file 24) and distribution of responsive regulatory elements (Fig. 8). However, most of the duplicated genes cannot be regulated by the same TF through gene regulatory networks (Fig. 9 and Additional files 29 and 30). Thus, sub-functionalization was the main evolutionary fate of duplicated GmPP2Cs. Most of these duplicated genes have kept the original or similar functions of the ancestral genes after duplication events. In particular, many duplicated genes are known to have similar evolutionary fates in plants [36, 37]. Moreover, the evolutionary rates of the duplicated genes were various in different GmPP2C subfamilies (Fig. 3F-H). Compared with other subfamilies, the H subfamily evolved slowest (Fig. 3F-H), had a high sequence similarity (Additional file 13) and lost less genes (Fig.4B). The slow evolutionary rates of the H subfamily resulted from low gene loss rate after polyploidization. Furthermore, some polarizing evolutionary rates were found in the same subfamily (Fig. 3F-H). The high Ka values could result from the low sequence similarity (Additional file 13B), and the high Ks values were related to the loss of the orthologous genes (Fig. 4B). Sequence similarity and gene loss affected the Ka/ Ks ratio.

The legume-common duplication event and soybean-specific tetraploid provided an expected 1:4 ratio of the orthologous regions between grape and soybean [25, 29, 30]. Only seven VvPP2Cs were in line with the expected ratio (Fig. 4A-B, and Additional files 19 and 20). Thus, the large-scale gene losses were recognized in the GmPP2C family. The widespread gene losses might be due to the transposon movements or poor assemblies and annotations [32, 33]. The gene losses could help in diploidization, including a series of chromosome breakages and fusions [34, 38, 39]. In addition, the gene losses were unevenly distributed across the GmPP2C subfamily (Fig. 4B). The H subfamily in soybean lost less GmPP2Cs, whereas the F subfamily had a higher gene loss rate. The gene loss rate was associated with the sequence similarity in the GmPP2C subfamily (Additional file 13). Natural selection might be a reason for the unbalanced gene retention rates [32].

The divergent time of GmPP2Cs and VvPP2Cs was significantly later than that of other gene families in soybean and grape (Fig. 4C-D). The evolutionary rates of the PP2C family were significantly lower than that of other genes in soybean and grape (Fig. 4D-F). Considering the slow evolutionary rate, GmPP2Cs and VvPP2Cs might have the highly conserved functions, i.e., protein dephosphorylation [2, 40]. Moreover, different PP2C subfamilies had the different evolutionary rates through the Ka, Ks and Ka/Ks analyses (Fig. 4G-I). The PP2C members from the H subfamily had the slowest evolutionary rates and most similar sequence to AtrPP2Cs and NcPP2Cs in two basal angiosperms (Figs. 4G-I, 5 and Additional files 21 and 22). Thus, the PP2C members from the H subfamily resembled their ancestral genes. This finding has been supported by more specific motifs (Fig. 2 and Additional files 11 and 12), highly fragmented gene structures (Fig. 2), slow evolutionary rates of the duplicated genes (Fig. 3), low gene loss rates (Fig. 4), high sequence similarity (Additional file 13) and the same regulatory TFs (Additional file 30).

The spatial expression analysis of the *GmPP2C* members revealed tissue-specific expression profiles (Fig. 6 and Additional file 23). Most of the *GmPP2C*s had a very broad expression spectrum, suggesting their very important roles in the regulation of plant growth and development. The PP2C family had the similar results in rice, Arabidopsis, *B.distachyon*, maize and cotton [7, 9, 11, 12]. Moreover, GCN analysis indicated certain key putative genes in growth and development (Fig. 6 and Additional files 25, 26, 27). *GmPP2C104*, *GmPP2C123* and *GmPP2C133* might participate in flower cell morphogenesis, and an orthologous gene of *GmPP2C123* (*AT5G02760*) could regulate the stamen filaments in Arabidopsis [41]. *GmPP2C024*, *GmPP2C035* and *GmPP2C091* directly co-expressed with certain plastid-related genes in chloroplast development, and *AT4G27800*, an orthologous gene of *GmPP2C024* and *GmPP2C035*, reportedly played key roles in LHCII dephosphorylation [42]. *GmPP2C029*, *GmPP2C038* and *GmPP2C117* might be very important to leaf development. The mutant of *AT2G28890* and *AT1G07630* (the *GmPP2C117* orthologous genes) had abnormally shaped leaves [43]. Meanwhile, *GmPP2C031*, *GmPP2C071* and *GmPP2C131* might be involved in seed development, and an orthologous gene of *GmPP2C071* (AT5G51760) could regulate seed germination in Arabidopsis [44]. *GmPP2C005*, *GmPP2C013*, *GmPP2C059* and *GmPP2C118* might play important roles in the nodulation development stage, and *LjNPP2C1* (a *GmPP2C005* orthologous gene) in *Lotus japonicus* was related to both early and late stages of nodule development [45]. In addition, most GmPP2Cs could respond to the hormone signaling through the *cis*-regulatory element analysis (Fig. 7 and Additional file 28). The GmPP2Cs from the A subfamily might participate in the ABA pathway. Some PP2Cs from the A subfamily reportedly regulated the ABA signaling in soybean and other plants [23, 27, 46]. Moreover, through the GRN analysis, 40 TFs from the WRKY family could regulate the *GmPP2C* expression (Fig. 8 and Additional file 29), indicating that the evolution of the GmPP2C family was directly or indirectly associated with the WRKY family.

## Conclusions

A total of 134 GmPP2C members with 10 subfamilies were found in the present study. The number of duplication events from the legume-common duplication event and soybean-specific tetraploid led to the GmPP2C expansion. All of the duplication events were the segmental duplication events. Sub-functionalization was the main evolutionary fate of duplicated PP2C members in soybean. Meanwhile, the GmPP2C family lost massive genes, especially in the F subfamily. The GmPP2C members evolved more slowly than other genes, and the PP2C members from the H subfamily resembled their ancestral genes. In addition, some genes were considered as the putative key candidates that could control plant growth and development (*GmPP2C104*, *GmPP2C123*, and *GmPP2C133* in flower cell morphogenesis; *GmPP2C024*, *GmPP2C035*, and *GmPP2C091* in chloroplast development; *GmPP2C029*, *GmPP2C038*, and *GmPP2C117* in leaf development; *GmPP2C031*, *GmPP2C071*, and *GmPP2C131* in seed development; *GmPP2C005*, *GmPP2C013*, *GmPP2C059* and *GmPP2C118* in nodulation development).

## Methods

### Sequence retrieval and sequence analysis

The genome sequences of *G. max*, *V. vinifera*, *A. thaliana* and *A. trichopoda* were downloaded from the Phytozome database (https://phytozome.jgi.doe.gov/pz/portal.html). The genome sequences of *N. colorata* were obtained from the Waterlily Pond (http://waterlily.eplant.org). The HMM profile of the PP2C domain (PF00481) from the Pfam database (https://pfam.xfam.org/) was used to identify the PP2C members in soybean, grape, Arabidopsis, *A. trichopoda* and *N. colorata* with hmmsearch tool in HMMER 3.0 program [47]. Then, all putative PP2C members were further confirmed through the CDD program (https://www.ncbi.nlm.nih.gov/cdd). The conserved motifs of the PP2C family in soybean were identified by the MEME program (http://meme-suite.org/tools/meme). The parameters were set according to the previous report [48]. The chromosomal distribution images and the exon–intron structures of the *GmPP2C* genes were visualized by the TBtools software [49]. “Intron phases” were defined through the intron–exon location. Phase 0, 1 and 2 defined introns inserted between codons, between the first and second nucleotides of the codon and between the second and third nucleotides of the codon, respectively [11]. Further, the sequence similarity of the PP2C family in soybean and two basal angiosperms were compared through the Blastp program with default parameters.

### Phylogenetic analysis

The PP2C proteins were aligned using the ClustalX software with default configurations [50]. The phylogenetic trees were constructed by four different tools. The maximum likelihood method was used with the FastTree version 2.1.3 using the Jones-Taylor-Thornton amino acid substitution model and the bootstrapping value [51]. The Bayesian analysis was performed through MrBayes version 3.1.2 with the mixed model and Bayesian posterior probabilities [52]. The neighbor-joining trees were analyzed by the PHYLIP and MEGA tool, and the reliability was assessed by a 1000 bootstrapping test [53, 54]. All trees were visualized with Figtree version 1.4.3.

### Syntenic and evolutionary rate analysis

Syntenic blocks were found by the MCScan software [55], and the syntenic relationship was visualized by the Circos program. The Ka, Ks and their ratio (Ka/Ks) were calculated with the Nei–Gojobori method by the TBtools software [49].

### Expression analysis

The RNA-seq transcriptome dataset of soybean in different tissues (pod, leaf, root, stem, nodule, seed, and flower) were downloaded from the Phytozome database, and more details about experimental materials and RNA-seq data analysis are described in the previous report [56]. The expression level was log-transformed via the log_2_(FPKM+ 1) function [57]. The expression data were hierarchically clustered through the hclust function in R.

*G. max* cv. Tianlong-1 was used to construct the expression profiles of the PP2C family in soybean. Roots, stems, and leaves were harvested from 4-week-old seedlings. RNA extraction and qRT-PCR analysis were performed on the basis of previous studies [48]. The *GmHELIC* gene was used as the reference gene, and gene-specific primers were designed on the basis of their coding sequences (Additional file 31). qRT–PCR analysis was conducted by three biological replications. Moreover, the PCCs of the RNA-seq and qRT-PCR analyses were calculated by cor function in R.

### Gene co-expression network analysis

The co-expression network was constructed by the weighted gene co-expression network analysis (WGCNA) in R [58]. The expression pattern in soybean used the same RNA-seq transcriptome dataset in the expression analysis. Genes with the low coefficient of variation (CV < 0.6) and the low maximum expression value (FPKM< 10) were discarded and all *GmPP2C* genes were added into the analysis. The remaining 13,954 genes were used for the WGCNA analysis. The co-expression networks were performed with default settings, except for those whose soft-threshold power was 12, minModuleSize was 30, and mergeCutHeight was 0.25. The node and edge information of GmPP2Cs with edge weight > 0.6 was extracted from the interested modules, and only the top 1000 weight values were selected. The sub-networks were analyzed and visualized using the Cytoscape software.

### GO and *cis*-element analysis

GO enrichment was performed using the GO Term Enrichment tool with *P* value ≤0.01 in PlantRegMap (http://plantregmap.cbi.pku.edu.cn/). Meanwhile, the 1000 bp upstream of the initiation codon (ATG) were extracted from the Phytozome database genes in the GmPP2C family. The potential responsive-regulatory elements in the *GmPP2C* promoters were identified by the PlantCARE database (http://bioinformatics.psb.ugent.be/webtools/plantcare/html/). The distributions of the responsive-regulatory elements were visualized by the TBtools software [49].

### Gene regulatory network analysis

GRN was performed by the MERLIN+Prior method [59]. The expression file used the same expression matrix in the WGCNA analysis. The soybean TFs as the regulator list were downloaded from the PlantTFDB database (http://planttfdb.cbi.pku.edu.cn/index.php). The TF binding site database in soybean as the prior networks was obtained from the PlantRegMap database. The WGCNA module was set as the initial cluster assignment. The GRN networks were analyzed and visualized by the Cytoscape software.

## Supplementary information

**Additional file 1 **Phylogenetic and sequence analysis of the PP2C members in soybean, Arabidopsis, grape, *A. trichopoda* and *N. colorata*. The inner circle was the phylogenetic tree using the FastTree method. The numbers in the clades were the FastTree bootstrap values. The PP2C subfamilies were labeled using different letters and were indicated using different colors. The outer circle was the sequence analysis of PP2Cs. The conserved PP2C domain was represented by the blue box. The location of each PP2C protein can be estimated using the scale at the end of the circle.

**Additional file 2 **GmPP2C proteins identified in *G. max*.

**Additional file 3 **VvPP2C proteins identified in *V. vinifera.*

**Additional file 4 **AtPP2C proteins identified in *A. thaliana*.

**Additional file 5 **AtrPP2C proteins identified in *A. trichopoda*.

**Additional file 6 **NcPP2C proteins identified in *N. colorata*.

**Additional file 7.** Phylogenetic relationship of the GmPP2C members. The neighbor-joining (NJ) tree was constructed through the MEGA program, and the numbers in the clades are the bootstrap values. The PP2C subfamilies are grouped by colors.

**Additional file 8.** Phylogenetic analysis of the GmPP2C members. The neighbor-joining (NJ) tree was generated through PHYLIP package, and the numbers in the clades are the bootstrap values. The PP2C subfamilies are grouped by colors.

**Additional file 9.** Percentage of GmPP2C numbers in the PP2C subfamily (upper) and its location distribution on each chromosome (bottom).

**Additional file 10.** Locations of the conserved PP2C domain in GmPP2Cs, VvPP2Cs, AtPP2Cs, AtrPP2Cs and NcPP2Cs.

**Additional file 11.** Motif information of GmPP2Cs through the MEME tool.

**Additional file 12.** Motif distributions in the GmPP2C subfamily.

**Additional file 13.** Sequence similarity of GmPP2Cs. (A) Sequence similarity of GmPP2Cs from the same and different subfamily. (B) Sequence similarity of duplicated GmPP2Cs in each subfamily.

**Additional file 14.** Structural analysis of GmPP2C in this study.

**Additional file 15.** Chromosomal locations of GmPP2Cs on all 20 chromosomes. The scale used is base pair (bp). Parentheses after the gene names show the PP2C subfamily.

**Additional file 16 **Genomic locations of the *GmPP2C* genes.

**Additional file 17.** Ka/Ks analysis for the duplicated PP2Cs in soybean.

**Additional file 18.** Ka/Ks analysis for the duplicated PP2Cs in grape.

**Additional file 19.** Ka/Ks analysis for GmPP2Cs and its corresponding orthologous genes in grape.

**Additional file 20.** Number of VvPP2C orthologous genes in soybean.

**Additional file 21.** Sequence similarity between GmPP2Cs and its corresponding AtrPP2Cs.

**Additional file 22.** Sequence similarity between GmPP2Cs and its corresponding NcPP2Cs.

**Additional file 23.** The FPKM values of GmPP2Cs in seven tissues.

**Additional file 24.** PCC of the FPKM values for the duplicated GmPP2Cs pairs.

**Additional file 25.** The constructed co-expression modules in soybean by hierarchical clustering and dynamic tree cut.

**Additional file 26.** Module information of GmPP2C through WGCNA analysis.

**Additional file 27.** Scatterplot of gene significance and module membership in certain significant modules.

**Additional file 28 **Number of the responsive-regulatory elements in the promoter regions of *GmPP2C*s.

**Additional file 29 **Regulatory relationship between TFs and *GmPP2C*s through the MERLIN+Prior method.

**Additional file 30 **Regulatory relationship between TFs and duplicated *GmPP2C*s through the MERLIN+Prior method.

**Additional file 31.** PCR primers used in the study.

## Data Availability

All data in this study are included in this article and its additional files.

## Abbreviations

ABA: Abscisic Acid
CV: Coefficient of variation
DSPTP: Dual-specificity phosphatase
FPKM: Fragments per Kilobase Million
GCN: Gene co-expression network
GO: Gene ontology
GRN: Gene regulatory network
HMM: Hidden Markov Model
Ka: Nonsynonymous substitution rate
Ks: Synonymous substitution rate
MeJA: Methyl Jasmonate
PP2C: Protein phosphatases 2C
PCC: Pearson Correlation Coefficient
PK: Protein kinase
PP: Protein phosphatase
PPM: Phosphoprotein metallophosphatase
PPP: Phosphor-protein phosphatase
PTP: Protein Tyr phosphatase
SA: Salicylic Acid
Ser: Serine
STP: Ser/Thr phosphatase
TF: Transcription factor
Thr: Threonine
Tyr: Tyrosine
WGCNA: Weighted gene co-expression network analysis

## References

[1] Hunter T (1995). Protein kinases and phosphatases: the yin and yang of protein phosphorylation and signaling. Cell.

[2] Cohen P (1989). The structure and regulation of protein phosphatases. Annu Rev Biochem.

[3] Kerk D, Templeton G, Moorhead GB (2008). Evolutionary radiation pattern of novel protein phosphatases revealed by analysis of protein data from the completely sequenced genomes of humans, green algae, and higher plants. Plant Physiol.

[4] Cohen P, Cohen PT (1989). Protein phosphatases come of age. J Biol Chem.

[5] Singh A, Pandey A, Srivastava AK, Tran LP, Pandey GK (2016). Plant protein phosphatases 2C: from genomic diversity to functional multiplicity and importance in stress management. Crit Rev Biotechnol.

[6] Schweighofer A, Hirt H, Meskiene I (2004). Plant PP2C phosphatases: emerging functions in stress signaling. Trends Plant Sci.

[7] Xue T, Wang D, Zhang S, Ehlting J, Ni F, Jakab S, Zheng C, Zhong Y (2008). Genome-wide and expression analysis of protein phosphatase 2C in rice and Arabidopsis. BMC Genomics.

[8] Singh A, Giri J, Kapoor S, Tyagi AK, Pandey GK (2010). Protein phosphatase complement in rice: genome-wide identification and transcriptional analysis under abiotic stress conditions and reproductive development. BMC Genomics.

[9] Cao J, Jiang M, Li P, Chu Z (2016). Genome-wide identification and evolutionary analyses of the PP2C gene family with their expression profiling in response to multiple stresses in *Brachypodium distachyon*. BMC Genomics.

[10] Yang Q, Liu K, Niu X, Wang Q, Wan Y, Yang F, Li G, Wang Y, Wang R (2018). Genome-wide identification of PP2C genes and their expression profiling in response to drought and cold stresses in *Medicago truncatula*. Sci Rep Uk.

[11] Fan K, Yuan S, Chen J, Chen Y, Li Z, Lin W, Zhang Y, Liu J, Lin W. Molecular evolution and lineage-specific expansion of the PP2C family in *Zea mays*. Planta. 2019;250:1521–38. 10.1007/s00425-019-03243-x 31346803

[12] Shazadee H, Khan N, Wang J, Wang C, Zeng J, Huang Z, Wang X (2019). Identification and expression profiling of protein phosphatases (PP2C) gene family in *Gossypium hirsutum* L. Int J Mol Sci.

[13] Song S, Hofhuis H, Lee MM, Clark SE (2008). Key divisions in the early Arabidopsis embryo require POL and PLL1 phosphatases to establish the root stem cell organizer and vascular axis. Dev Cell.

[14] Stone JM, Trotochaud AE, Walker JC, Clark SE (1998). Control of meristem development by CLAVATA1 receptor kinase and kinase-associated protein phosphatase interactions. Plant Physiol.

[15] Gagne JM, Clark SE (2010). The Arabidopsis stem cell factor POLTERGEIST is membrane localized and phospholipid stimulated. Plant Cell.

[16] Kim W, Lee Y, Park J, Lee N, Choi G (2013). HONSU, a protein phosphatase 2C, regulates seed dormancy by inhibiting ABA signaling in Arabidopsis. Plant Cell Physiol.

[17] Spartz AK, Ren H, Park MY, Grandt KN, Lee SH, Murphy AS, Sussman MR, Overvoorde PJ, Gray WM (2014). SAUR inhibition of PP2C-D phosphatases activates plasma membrane H^+^-ATPases to promote cell expansion in Arabidopsis. Plant Cell.

[18] Lee MW, Jelenska J, Greenberg JT (2008). Arabidopsis proteins important for modulating defense responses to *Pseudomonas syringae* that secrete HopW1-1. Plant J.

[19] Schweighofer A, Kazanaviciute V, Scheikl E, Teige M, Doczi R, Hirt H, Schwanninger M, Kant M, Schuurink R, Mauch F (2007). The PP2C-type phosphatase AP2C1, which negatively regulates MPK4 and MPK6, modulates innate immunity, jasmonic acid, and ethylene levels in Arabidopsis. Plant Cell.

[20] Bhaskara GB, Wen T, Nguyen TT, Verslues PE (2017). Protein phosphatase 2Cs and microtubule-associated stress protein 1 control microtubule stability, plant growth, and drought response. Plant Cell.

[21] Manabe Y, Bressan RA, Wang T, Li F, Koiwa H, Sokolchik I, Li X, Maggio A (2008). The Arabidopsis kinase-associated protein phosphatase regulates adaptation to Na^+^ stress. Plant Physiol.

[22] Lenka SK, Muthusamy SK, Chinnusamy V, Bansal KC. Ectopic expression of rice PYL3 enhances cold and drought tolerance in *Arabidopsis thaliana*. Mol Biotechnol. 2018;60:350–61. 10.1007/s12033-018-0076-5 29574592

[23] Ma Y, Szostkiewicz I, Korte A, Moes D, Yang Y, Christmann A, Grill E (2009). Regulators of PP2C phosphatase activity function as abscisic acid sensors. Science.

[24] Manohar M, Wang D, Manosalva PM, Choi HW, Kombrink E, Klessig DF (2017). Members of the abscisic acid co-receptor PP 2C protein family mediate salicylic acid–abscisic acid crosstalk. Plant Direct.

[25] Schmutz J, Cannon SB, Schlueter J, Ma J, Mitros T, Nelson W, Hyten DL, Song Q, Thelen JJ, Cheng J (2010). Genome sequence of the palaeopolyploid soybean. Nature.

[26] Lu X, Xiong Q, Cheng T, Li Q, Liu X, Bi Y, Li W, Zhang W, Ma B, Lai Y (2017). A PP2C-1 allele underlying a quantitative trait locus enhances soybean 100-seed weight. Mol Plant.

[27] Bai G, Yang D, Zhao Y, Ha S, Yang F, Ma J, Gao X, Wang Z, Zhu J (2013). Interactions between soybean ABA receptors and type 2C protein phosphatases. Plant Mol Biol.

[28] Bhalothia P, Lata S, Khan ZH, Kumar B, Mehrotra S, Mehrotra R (2018). Genome wide analysis of protein phosphatase 2C (PP2C) genes in *Glycine max* and *Sorghum bicolor*. Current Biotechnology.

[29] Jaillon O, Aury J, Noel B, Policriti A, Clepet C, Casagrande A, Choisne N, Aubourg S, Vitulo N, Jubin C (2007). The grapevine genome sequence suggests ancestral hexaploidization in major angiosperm phyla. Nature.

[30] Wang J, Sun P, Li Y, Liu Y, Yu J, Ma X, Sun S, Yang N, Xia R, Lei T (2017). Hierarchically aligning 10 legume genomes establishes a family-level genomics platform. Plant Physiol.

[31] Wang Y, Wang X, Tang H, Tan X, Ficklin SP, Feltus FA, Paterson AH (2011). Modes of gene duplication contribute differently to genetic novelty and redundancy, but show parallels across divergent angiosperms. PLoS One.

[32] Albalat R, Cañestro C (2016). Evolution by gene loss. Nat Rev Genet.

[33] Soltis DE, Visger CJ, Marchant DB, Soltis PS (2016). Polyploidy: pitfalls and paths to a paradigm. Am J Bot.

[34] Otto SP (2007). The evolutionary consequences of polyploidy. Cell.

[35] Blanc G, Wolfe KH (2004). Widespread paleopolyploidy in model plant species inferred from age distributions of duplicate genes. Plant Cell.

[36] Lynch M, Conery JS (2000). The evolutionary fate and consequences of duplicate genes. Science.

[37] Small RL, Wendel JF (2002). Differential evolutionary dynamics of duplicated paralogous *Adh* loci in allotetraploid cotton (Gossypium). Mol Biol Evol.

[38] Albalat R, Cañestro C (2016). Evolution by gene loss. Nat Rev Genet.

[39] Soltis PS, Soltis DE (2009). The role of hybridization in plant speciation. Annu Rev Plant Biol.

[40] MacKintosh C, Coggins J, Cohen P (1991). Plant protein phosphatases. Subcellular distribution, detection of protein phosphatase 2C and identification of protein phosphatase 2A as the major quinate dehydrogenase phosphatase. Biochem J.

[41] Wong JH, Spartz AK, Park MY, Du M, Gray WM. Mutation of a conserved motif of PP2C.D phosphatases confers SAUR immunity and constitutive activity. Plant Physiol. 2019;181:353–66. 10.1104/pp.19.00496 PMC6716246 31311832

[42] Pribil M, Pesaresi P, Hertle A, Barbato R, Leister D (2010). Role of plastid protein phosphatase TAP38 in LHCII dephosphorylation and thylakoid electron flow. PLoS Biol.

[43] Song S, Clark SE (2005). POL and related phosphatases are dosage-sensitive regulators of meristem and organ development in Arabidopsis. Dev Biol.

[44] Née G, Kramer K, Nakabayashi K, Yuan B, Xiang Y, Miatton E, Finkemeier I, Soppe W (2017). DELAY OF GERMINATION1 requires PP2C phosphatases of the ABA signalling pathway to control seed dormancy. Nat Commun.

[45] Kapranov P, Jensen T, Poulsen C, De Bruijn F, Szczyglowski K (1999). A protein phosphatase 2C gene, *LjNPP2C1*, from *Lotus japonicus* induced during root nodule development. P Natl Acad Sci USA.

[46] Xiang Y, Sun X, Gao S, Qin F, Dai M (2017). Deletion of an endoplasmic reticulum stress response element in a ZmPP2C-A gene facilitates drought tolerance of maize seedlings. Mol Plant.

[47] Finn RD, Clements J, Eddy SR (2011). HMMER web server: interactive sequence similarity searching. Nucleic Acids Res.

[48] Fan K, Shen H, Bibi N, Li F, Yuan S, Wang M, Wang X (2015). Molecular evolution and species-specific expansion of the NAP members in plants. J Integr Plant Biol.

[49] Chen C, Xia R, Chen H, He Y. TBtools, a toolkit for biologists integrating various biological data handling tools with a user-friendly interface. BioRxiv. 2018. https://www.biorxiv.org/content/10.1101/289660v1.

[50] Larkin MA, Blackshields G, Brown NP, Chenna R, McGettigan PA, McWilliam H, Valentin F, Wallace IM, Wilm A, Lopez R (2007). Clustal W and Clustal X version 2.0. Bioinformatics.

[51] Price MN, Dehal PS, Arkin AP (2010). FastTree 2–approximately maximum-likelihood trees for large alignments. PLoS One.

[52] Ronquist F, Huelsenbeck JP (2003). MrBayes 3: Bayesian phylogenetic inference under mixed models. Bioinformatics.

[53] Kumar S, Stecher G, Li M, Knyaz C, Tamura K (2018). MEGA X: molecular evolutionary genetics analysis across computing platforms. Mol Biol Evol.

[54] Plotree D, Plotgram D (1989). PHYLIP-phylogeny inference package (version 3.2). Cladistics.

[55] Tang H, Bowers JE, Wang X, Ming R, Alam M, Paterson AH (2008). Synteny and collinearity in plant genomes. Science.

[56] Wang J, Hossain MS, Lyu Z, Schmutz J, Stacey G, Xu D, Joshi T (2019). SoyCSN: soybean context-specific network analysis and prediction based on tissue-specific transcriptome data. Plant Direct.

[57] Ma S, Ding Z, Li P (2017). Maize network analysis revealed gene modules involved in development, nutrients utilization, metabolism, and stress response. BMC Plant Biol.

[58] Langfelder P, Horvath S (2008). WGCNA: an R package for weighted correlation network analysis. BMC Bioinformatics.

[59] Siahpirani AF, Roy S (2016). A prior-based integrative framework for functional transcriptional regulatory network inference. Nucleic Acids Res.

