# Supplementary material for: Pervasive duplication, biased molecular evolution and comprehensive functional analysis of the PP2C family in Glycine max
Source: BMC Genomics. 2020 Jul 6;21:465. doi: 10.1186/s12864-020-06877-4 (PMC7339511; doi:10.1186/s12864-020-06877-4)
Supplement: Supplementary file 21 — Additional file 21. Sequence similarity between GmPP2Cs and its corresponding AtrPP2Cs. [file 12864_2020_6877_MOESM21_ESM.pdf]

Additional file 21. Sequence similarity between GmPP2Cs and its corresponding AtrPP2Cs.

Gene1	Gene2	Subfamily	Identity	Bit_score
GmPP2C001	AtrPP2C12	E	52.41	325
GmPP2C002	AtrPP2C35	J	70.66	1516
GmPP2C003	AtrPP2C19	G	66.76	470
GmPP2C004	AtrPP2C01	D	55.62	404
GmPP2C005	AtrPP2C39	A	49	259
GmPP2C007	AtrPP2C03	G	59.2	448
GmPP2C008	AtrPP2C01	D	78.01	615
GmPP2C011	AtrPP2C21	D	65.36	513
GmPP2C012	AtrPP2C29	B	54.68	387
GmPP2C013	AtrPP2C07	A	56	392
GmPP2C014	AtrPP2C11	D	64.92	490
GmPP2C015	AtrPP2C32	H	73.72	613
GmPP2C016	AtrPP2C26	E	59.34	459
GmPP2C017	AtrPP2C19	G	61.7	420
GmPP2C018	AtrPP2C37	H	72.2	608
GmPP2C019	AtrPP2C39	A	46.89	451
GmPP2C020	AtrPP2C10	F	80.35	474
GmPP2C021	AtrPP2C36	G	60.2	448
GmPP2C022	AtrPP2C17	F	74.82	435
GmPP2C023	AtrPP2C19	G	79.38	273
GmPP2C025	AtrPP2C17	F	76.1	435
GmPP2C026	AtrPP2C24	A	57.62	360
GmPP2C027	AtrPP2C19	G	70.83	559
GmPP2C028	AtrPP2C37	H	73.58	625
GmPP2C029	AtrPP2C16	E	52.19	347
GmPP2C030	AtrPP2C39	A	47.8	455
GmPP2C031	AtrPP2C10	F	81.4	478

GmPP2C032	AtrPP2C38	I	83.04	602
GmPP2C033	AtrPP2C36	G	61.98	449
GmPP2C034	AtrPP2C17	F	75.82	442
GmPP2C036	AtrPP2C41	F	69.5	409
GmPP2C037	AtrPP2C40	E	51.25	475
GmPP2C038	AtrPP2C38	I	79.1	573
GmPP2C039	AtrPP2C08	C	37.5	250
GmPP2C040	AtrPP2C03	G	53.18	389
GmPP2C041	AtrPP2C04	D	71.73	573
GmPP2C042	AtrPP2C26	E	52.65	424
GmPP2C043	AtrPP2C22	C	61.02	617
GmPP2C044	AtrPP2C13	J	65.65	486
GmPP2C045	AtrPP2C24	A	60.31	376
GmPP2C046	AtrPP2C17	F	75.74	432
GmPP2C047	AtrPP2C41	F	56.27	309
GmPP2C048	AtrPP2C17	F	77.57	444
GmPP2C049	AtrPP2C38	I	79.4	578
GmPP2C050	AtrPP2C03	G	54.7	392
GmPP2C051	AtrPP2C04	D	71.47	566
GmPP2C052	AtrPP2C22	C	63.43	492
GmPP2C053	AtrPP2C39	A	61.76	409
GmPP2C054	AtrPP2C36	G	54.66	345
GmPP2C055	AtrPP2C21	D	69.01	550
GmPP2C057	AtrPP2C35	J	68.5	1507
GmPP2C058	AtrPP2C40	E	66.81	641
GmPP2C059	AtrPP2C12	E	52.73	405
GmPP2C060	AtrPP2C03	G	59.16	445
GmPP2C061	AtrPP2C11	D	65.32	508
GmPP2C062	AtrPP2C32	H	73.96	622

GmPP2C063	AtrPP2C26	E	57.26	441
GmPP2C064	AtrPP2C01	D	59.52	412
GmPP2C065	AtrPP2C32	H	81.54	728
GmPP2C066	AtrPP2C01	D	79.39	629
GmPP2C067	AtrPP2C09	F	72.14	404
GmPP2C068	AtrPP2C11	D	67.83	532
GmPP2C069	AtrPP2C28	E	72.03	213
GmPP2C071	AtrPP2C39	A	49.49	253
GmPP2C072	AtrPP2C01	D	55.91	400
GmPP2C073	AtrPP2C19	G	67.05	474
GmPP2C074	AtrPP2C08	C	39.85	216
GmPP2C075	AtrPP2C29	B	62.67	376
GmPP2C076	AtrPP2C29	B	62.67	376
GmPP2C077	AtrPP2C07	A	57.63	412
GmPP2C078	AtrPP2C40	E	50.63	471
GmPP2C079	AtrPP2C41	F	70.57	422
GmPP2C080	AtrPP2C40	E	49.17	442
GmPP2C081	AtrPP2C41	F	64.89	377
GmPP2C082	AtrPP2C42	F	68.73	498
GmPP2C083	AtrPP2C39	A	60.18	410
GmPP2C084	AtrPP2C11	D	65.05	508
GmPP2C085	AtrPP2C36	G	52.82	353
GmPP2C086	AtrPP2C13	J	65.07	472
GmPP2C087	AtrPP2C41	F	65.96	399
GmPP2C088	AtrPP2C40	E	50.5	456
GmPP2C089	AtrPP2C07	A	57.07	398
GmPP2C090	AtrPP2C37	H	74.29	627
GmPP2C091	AtrPP2C38	I	77.98	560
GmPP2C092	AtrPP2C10	F	81.53	482

GmPP2C093	AtPP2C39	A	55.97	470
GmPP2C094	AtPP2C42	F	68.17	496
GmPP2C095	AtPP2C07	A	52.74	266
GmPP2C096	AtPP2C29	B	53.3	379
GmPP2C097	AtPP2C17	F	78.07	443
GmPP2C098	AtPP2C13	J	65.07	473
GmPP2C099	AtPP2C04	D	71.85	563
GmPP2C100	AtPP2C39	A	62.15	414
GmPP2C101	AtPP2C36	G	55.28	355
GmPP2C102	AtPP2C01	D	77.32	610
GmPP2C103	AtPP2C13	J	63.89	478
GmPP2C104	AtPP2C22	C	62.22	624
GmPP2C105	AtPP2C26	E	52.94	423
GmPP2C106	AtPP2C04	D	71.54	571
GmPP2C107	AtPP2C03	G	53.75	392
GmPP2C108	AtPP2C39	A	60.49	411
GmPP2C109	AtPP2C36	G	52.68	358
GmPP2C110	AtPP2C39	A	55.53	463
GmPP2C111	AtPP2C10	F	81.53	483
GmPP2C112	AtPP2C38	I	77.68	557
GmPP2C113	AtPP2C16	E	52.46	341
GmPP2C114	AtPP2C37	H	74.76	629
GmPP2C115	AtPP2C07	A	59.51	416
GmPP2C116	AtPP2C29	B	62.63	380
GmPP2C117	AtPP2C22	C	37.04	249
GmPP2C117	AtPP2C08	C	37.95	249
GmPP2C118	AtPP2C12	E	57.14	388
GmPP2C119	AtPP2C40	E	67.49	648
GmPP2C120	AtPP2C40	E	66.15	509

GmPP2C121	AtrPP2C07	A	53.42	267
GmPP2C122	AtrPP2C21	D	64.18	530
GmPP2C123	AtrPP2C11	D	63.93	504
GmPP2C124	AtrPP2C32	H	74.15	629
GmPP2C125	AtrPP2C26	E	57.37	458
GmPP2C126	AtrPP2C01	D	78.88	628
GmPP2C127	AtrPP2C32	H	82.01	730
GmPP2C128	AtrPP2C01	D	55.12	399
GmPP2C129	AtrPP2C26	E	57.26	444
GmPP2C130	AtrPP2C32	H	74.45	627
GmPP2C131	AtrPP2C09	F	66.67	336
GmPP2C132	AtrPP2C11	D	67.56	530
GmPP2C133	AtrPP2C40	E	56.35	424
GmPP2C134	AtrPP2C41	F	70.57	416
