# Supplementary material for: Pervasive duplication, biased molecular evolution and comprehensive functional analysis of the PP2C family in Glycine max
Source: BMC Genomics. 2020 Jul 6;21:465. doi: 10.1186/s12864-020-06877-4 (PMC7339511; doi:10.1186/s12864-020-06877-4)
Supplement: Supplementary file 22 — Additional file 22. Sequence similarity between GmPP2Cs and its corresponding NcPP2Cs. [file 12864_2020_6877_MOESM22_ESM.pdf]

Additional file 22. Sequence similarity between GmPP2Cs and its corresponding NcPP2Cs.

Gene1	Gene2	Subfamily	Identity	Bit_score
GmPP2C001	NcPP2C27	E	53.29	328
GmPP2C002	NcPP2C06	J	69.92	1499
GmPP2C003	NcPP2C36	G	62.87	434
GmPP2C004	NcPP2C39	D	55.85	395
GmPP2C005	NcPP2C30	A	50.4	226
GmPP2C007	NcPP2C36	G	50.83	349
GmPP2C008	NcPP2C39	D	72.77	609
GmPP2C011	NcPP2C17	D	66.58	523
GmPP2C012	NcPP2C31	B	45.15	292
GmPP2C013	NcPP2C30	A	47.7	348
GmPP2C014	NcPP2C26	D	65.47	492
GmPP2C015	NcPP2C12	H	72.66	612
GmPP2C016	NcPP2C10	E	58.5	428
GmPP2C017	NcPP2C36	G	59.82	395
GmPP2C018	NcPP2C32	H	76.27	518
GmPP2C019	NcPP2C28	A	45.24	424
GmPP2C020	NcPP2C23	F	80.69	470
GmPP2C021	NcPP2C36	G	52.07	348
GmPP2C022	NcPP2C33	F	74.22	404
GmPP2C023	NcPP2C18	G	77.5	250
GmPP2C025	NcPP2C33	F	78.26	411
GmPP2C026	NcPP2C03	A	48.38	262
GmPP2C027	NcPP2C36	G	67.36	517
GmPP2C028	NcPP2C32	H	76.27	525
GmPP2C029	NcPP2C27	E	51.27	321
GmPP2C030	NcPP2C28	A	44.8	419
GmPP2C031	NcPP2C23	F	81.94	474

GmPP2C032	NcPP2C25	I	67.51	534
GmPP2C033	NcPP2C36	G	52.05	354
GmPP2C034	NcPP2C33	F	75.29	413
GmPP2C036	NcPP2C05	F	71.73	419
GmPP2C037	NcPP2C21	E	50.33	426
GmPP2C038	NcPP2C25	I	61.94	504
GmPP2C039	NcPP2C19	C	37.96	241
GmPP2C040	NcPP2C36	G	47.44	317
GmPP2C041	NcPP2C09	D	62.98	497
GmPP2C042	NcPP2C10	E	52.78	417
GmPP2C043	NcPP2C19	C	58.25	545
GmPP2C044	NcPP2C38	J	66.94	495
GmPP2C045	NcPP2C28	A	45.57	262
GmPP2C046	NcPP2C33	F	77.87	409
GmPP2C047	NcPP2C11	F	57.98	317
GmPP2C048	NcPP2C33	F	77.47	404
GmPP2C049	NcPP2C25	I	63.18	509
GmPP2C050	NcPP2C36	G	51.85	320
GmPP2C051	NcPP2C09	D	62.47	490
GmPP2C052	NcPP2C19	C	59	449
GmPP2C053	NcPP2C28	A	57.62	369
GmPP2C054	NcPP2C36	G	54.46	333
GmPP2C055	NcPP2C35	D	70.57	563
GmPP2C057	NcPP2C06	J	67.61	1479
GmPP2C058	NcPP2C21	E	59.88	578
GmPP2C059	NcPP2C27	E	54.01	437
GmPP2C060	NcPP2C36	G	55.45	354
GmPP2C061	NcPP2C16	D	63.1	486
GmPP2C062	NcPP2C12	H	73.71	620

GmPP2C063	NcPP2C10	E	58.38	431
GmPP2C064	NcPP2C39	D	59.21	419
GmPP2C065	NcPP2C12	H	82.35	722
GmPP2C066	NcPP2C39	D	74.94	620
GmPP2C067	NcPP2C01	F	73.56	399
GmPP2C068	NcPP2C16	D	65.45	519
GmPP2C069	NcPP2C21	E	69.78	196
GmPP2C071	NcPP2C30	A	45.19	228
GmPP2C072	NcPP2C39	D	56.64	395
GmPP2C073	NcPP2C36	G	62.87	436
GmPP2C074	NcPP2C19	C	38.75	191
GmPP2C075	NcPP2C31	B	47.25	276
GmPP2C076	NcPP2C31	B	47.25	276
GmPP2C077	NcPP2C30	A	49.07	357
GmPP2C078	NcPP2C21	E	50.54	429
GmPP2C079	NcPP2C05	F	72.44	420
GmPP2C080	NcPP2C21	E	47.21	415
GmPP2C081	NcPP2C11	F	66.9	389
GmPP2C082	NcPP2C08	F	69.48	494
GmPP2C083	NcPP2C28	A	53.01	354
GmPP2C084	NcPP2C16	D	63.37	487
GmPP2C085	NcPP2C36	G	55.26	337
GmPP2C086	NcPP2C38	J	65.63	482
GmPP2C087	NcPP2C11	F	69.72	419
GmPP2C088	NcPP2C21	E	47.27	413
GmPP2C089	NcPP2C30	A	50	355
GmPP2C090	NcPP2C12	H	60.38	530
GmPP2C091	NcPP2C25	I	61.3	479
GmPP2C092	NcPP2C23	F	82.41	479

GmPP2C093	NcPP2C28	A	46.58	434
GmPP2C094	NcPP2C08	F	68.9	492
GmPP2C095	NcPP2C03	A	48.73	254
GmPP2C096	NcPP2C31	B	45.65	288
GmPP2C097	NcPP2C33	F	76.49	398
GmPP2C098	NcPP2C38	J	65.34	479
GmPP2C099	NcPP2C09	D	62.27	490
GmPP2C100	NcPP2C28	A	51.27	371
GmPP2C101	NcPP2C36	G	54.13	332
GmPP2C102	NcPP2C39	D	72.77	607
GmPP2C103	NcPP2C38	J	66.2	487
GmPP2C104	NcPP2C19	C	58.9	551
GmPP2C105	NcPP2C10	E	52.78	414
GmPP2C106	NcPP2C09	D	63.59	498
GmPP2C107	NcPP2C36	G	48.52	322
GmPP2C108	NcPP2C28	A	53.15	357
GmPP2C109	NcPP2C36	G	53.95	330
GmPP2C110	NcPP2C28	A	45.7	434
GmPP2C111	NcPP2C23	F	82.41	480
GmPP2C112	NcPP2C25	I	60.52	474
GmPP2C113	NcPP2C27	E	49.52	299
GmPP2C114	NcPP2C12	H	60.38	530
GmPP2C115	NcPP2C30	A	51.16	372
GmPP2C116	NcPP2C31	B	45.78	279
GmPP2C117	NcPP2C19	C	37.96	239
GmPP2C118	NcPP2C27	E	60.44	416
GmPP2C119	NcPP2C21	E	59.96	577
GmPP2C120	NcPP2C21	E	64.15	496
GmPP2C121	NcPP2C30	A	49.82	253

GmPP2C122	NcPP2C35	D	66.15	538
GmPP2C123	NcPP2C16	D	64.71	501
GmPP2C124	NcPP2C12	H	73.56	631
GmPP2C125	NcPP2C10	E	57.71	428
GmPP2C126	NcPP2C39	D	74.44	618
GmPP2C127	NcPP2C12	H	82.59	723
GmPP2C128	NcPP2C39	D	56.52	407
GmPP2C129	NcPP2C10	E	57.23	425
GmPP2C130	NcPP2C12	H	74.2	625
GmPP2C131	NcPP2C01	F	64.23	309
GmPP2C132	NcPP2C16	D	65.45	518
GmPP2C133	NcPP2C21	E	55.26	397
GmPP2C134	NcPP2C11	F	71.48	422
