# Supplementary material for: Pervasive duplication, biased molecular evolution and comprehensive functional analysis of the PP2C family in Glycine max
Source: BMC Genomics. 2020 Jul 6;21:465. doi: 10.1186/s12864-020-06877-4 (PMC7339511; doi:10.1186/s12864-020-06877-4)
Supplement: Supplementary file 23 — Additional file 23. The FPKM values of GmPP2Cs in seven tissues. [file 12864_2020_6877_MOESM23_ESM.pdf]

Additional file 23. The FPKM values of GmPP2Cs in seven tissues.

Name	Subfamily	Pod	Leaf	Root	Stem	Nodule	Seed	Flower
GmPP2C001	E	0.136752	0	0.039916	0.042031	0.106734	0.171735	0.089241
GmPP2C002	J	6.87149	8.04084	1.68318	11.232	3.72505	7.91198	7.08524
GmPP2C003	G	0.711729	0.795954	0.509789	0.445384	0.437926	0.087573	0.445023
GmPP2C004	D	2.3279	4.83638	0.142473	11.1392	1.26684	0.296531	2.42068
GmPP2C005	A	0.060646	0.027193	0.203645	0	1.81538	0.397755	0.181407
GmPP2C006	No group	6.58432	5.3018	6.19943	6.02496	7.52953	7.31755	7.57337
GmPP2C007	G	5.78866	9.43503	21.7249	8.97185	7.36644	4.4519	9.41067
GmPP2C008	D	25.4537	23.0304	21.9801	26.3492	18.4218	23.7799	17.5316
GmPP2C009	No group	4.62973	4.09545	1.29378	3.48654	3.64402	5.26204	4.72839
GmPP2C010	No group	7.27342	10.4455	2.88362	7.23571	3.9706	9.41141	5.59424
GmPP2C011	D	9.05041	5.03707	15.3248	7.91925	16.3148	3.97799	6.02923
GmPP2C012	B	2.06904	11.7445	15.2713	7.70475	6.51314	1.3623	15.8528
GmPP2C013	A	16.9122	17.78	21.232	19.5695	6.2287	22.3783	20.7574
GmPP2C014	D	1.04963	3.95481	0.987752	3.41565	0.789929	1.3015	4.90985
GmPP2C015	H	8.9687	0.658988	3.71221	8.07151	4.32871	13.3065	1.37341
GmPP2C016	E	7.54665	7.30553	22.8016	12.2766	6.68668	9.23399	6.75823
GmPP2C017	G	4.98291	7.15703	10.9231	5.33972	6.99552	2.56745	8.40728
GmPP2C018	H	1.8582	0.429641	0.920871	5.13965	3.04255	1.8959	1.06399
GmPP2C019	A	4.18357	10.2316	11.378	18.2663	6.69528	8.49502	10.168
GmPP2C020	F	15.5222	35.0969	23.4336	22.8883	14.6259	11.1264	44.3835
GmPP2C021	G	1.84889	2.48414	7.09188	3.23496	3.29544	2.41891	2.19514
GmPP2C022	F	1.88738	7.15051	7.88897	3.23367	6.40755	1.9732	5.82457
GmPP2C023	G	0.001266	0.000312	0.002073	0.061419	0.0009	0.000482	0.00073
GmPP2C024	No group	2.11087	22.6277	0.839585	3.59029	0.243557	4.693	1.60515
GmPP2C025	F	9.68698	35.7505	42.8344	15.2678	16.3721	6.48166	41.4714
GmPP2C026	A	3.27106	2.38636	3.40968	2.81738	0.980353	1.71228	2.51733
GmPP2C027	G	3.92912	6.08205	12.8341	7.63486	8.07054	2.9112	15.4662

GmPP2C028	H	1.60778	0.608587	7.30703	3.55322	3.13687	2.14026	0.698486
GmPP2C029	E	0	0	97.123	0.014645	14.017	0	2.21643
GmPP2C030	A	4.15345	11.959	7.6519	22.1755	5.71383	4.46541	5.68511
GmPP2C031	F	2.96147	2.85925	4.05606	2.94847	6.59752	22.4966	9.70819
GmPP2C032	I	34.6938	28.1626	105.433	42.2493	38.3029	21.2314	75.4017
GmPP2C033	G	1.10478	1.23664	4.67355	1.20281	2.57876	2.38048	2.97775
GmPP2C034	F	1.71239	6.77604	6.4036	4.04103	9.79527	2.59951	6.56355
GmPP2C035	No group	2.98111	22.7265	0.96659	3.97051	0.384092	5.91057	2.21971
GmPP2C036	F	13.7498	17.2558	8.69881	26.3654	13.1793	22.4951	12.8139
GmPP2C037	E	0.336741	31.4086	45.4811	3.12768	9.22142	0.191388	10.2544
GmPP2C038	I	17.0215	15.9758	29.0475	14.2587	17.7539	16.9363	16.1798
GmPP2C039	C	0.042897	0.032116	0.714996	0.015646	0.806264	0.035004	0.059455
GmPP2C040	G	2.75709	7.86998	5.23771	3.14807	3.06112	3.11202	15.5855
GmPP2C041	D	13.1743	7.14796	12.5065	20.1274	6.76079	8.71382	5.92945
GmPP2C042	E	0.914569	8.596	0.790758	3.28407	1.34791	0	3.09691
GmPP2C043	C	4.51959	1.24418	1.01842	3.13255	3.04849	1.53819	5.17967
GmPP2C044	J	6.81589	5.30349	6.62091	5.57722	5.3937	4.6901	9.31075
GmPP2C045	A	3.50896	5.08402	2.58906	1.30165	1.88783	1.4527	5.13686
GmPP2C046	F	11.6143	42.2909	53.1279	21.2688	19.963	7.37162	24.1296
GmPP2C047	F	0.102127	0.17626	0.629612	0.058574	0.249164	0.093141	0.166369
GmPP2C048	F	4.10915	9.61465	29.1991	6.48446	11.0706	2.26138	7.19356
GmPP2C049	I	14.2222	20.0656	24.4795	16.0883	18.9055	21.8864	22.933
GmPP2C050	G	8.39168	8.21873	4.10605	7.1373	4.365	4.4673	38.3282
GmPP2C051	D	4.27735	5.67252	4.94917	5.72346	4.69837	4.74586	5.23366
GmPP2C052	C	5.55399	4.87809	1.9284	3.06479	1.87347	5.09436	3.29878
GmPP2C053	A	5.49387	20.7764	22.7099	4.22748	3.9243	4.40983	27.9862
GmPP2C054	G	3.7373	3.26964	8.36182	2.20625	2.038	0.869379	2.01329
GmPP2C055	D	11.0481	5.14855	12.76	8.60253	8.98553	2.82423	5.07569
GmPP2C056	No group	5.31186	4.60463	5.40646	5.14427	5.80698	4.78624	5.18583

GmPP2C057	J	13.417	10.3958	4.80619	15.9379	6.34813	12.1759	10.1955
GmPP2C058	E	10.8553	18.553	13.4111	11.7999	8.74916	8.71761	6.42363
GmPP2C059	E	0.683274	1.69864	1.00042	1.85257	16.8921	3.14969	8.55167
GmPP2C060	G	7.33142	13.5124	33.0301	9.42696	8.75407	4.11459	14.4014
GmPP2C061	D	5.5364	6.63352	1.28671	14.7733	11.2215	1.90563	14.0888
GmPP2C062	H	13.9027	5.34847	18.7561	16.273	13.3008	16.5025	7.86095
GmPP2C063	E	5.41427	15.1156	11.8527	2.53845	1.35788	15.8356	6.41188
GmPP2C064	D	8.03918	12.5889	17.9378	9.38691	5.72123	3.44857	20.5447
GmPP2C065	H	5.94301	3.1588	9.2418	5.07426	9.3461	9.54725	3.10199
GmPP2C066	D	25.3677	46.7829	111.869	39.6222	51.5607	22.0294	23.1623
GmPP2C067	F	8.96745	7.75337	10.0715	7.91674	8.40847	11.3772	7.52167
GmPP2C068	D	0.398323	1.29032	0.710724	0.413717	0.896883	0.325627	2.08506
GmPP2C069	E	1.73372	2.98363	5.02632	1.34425	0.306224	0.764292	5.19891
GmPP2C070	No group	5.57229	5.90454	8.36984	6.21028	7.10041	6.15707	7.0587
GmPP2C071	A	0.059405	0.482642	2.00781	0.327312	0.092462	5.11676	0.482815
GmPP2C072	D	2.9448	13.2754	4.58698	12.9052	2.48459	0.355471	4.82278
GmPP2C073	G	0.363555	0.399942	0.751902	0.509299	0.41317	0.052927	0.10925
GmPP2C074	C	2.5626	5.45899	14.9298	4.06058	7.62828	2.86033	8.46818
GmPP2C075	B	0	0	0.126318	0	0.002665	0	1.35871
GmPP2C076	B	0	0	0.427982	0	0.041307	0	2.28163
GmPP2C077	A	7.27962	6.82833	23.5322	15.2035	7.4764	22.7689	35.3038
GmPP2C078	E	20.2438	5.25407	5.47005	22.3487	21.4799	18.2329	9.29986
GmPP2C079	F	0.457536	7.267	44.7926	8.18455	10.2001	0.868182	16.493
GmPP2C080	E	11.7271	2.40195	2.59205	5.42566	3.12683	16.6178	6.61564
GmPP2C081	F	14.4632	43.2504	40.5954	16.501	20.3497	12.9506	12.1595
GmPP2C082	F	21.8712	12.0786	18.7616	22.9055	18.3061	26.015	13.6723
GmPP2C083	A	8.31128	12.0773	17.9831	11.6516	10.4465	3.462	29.2903
GmPP2C084	D	6.68346	4.9357	1.45335	2.54319	6.75555	0.177946	4.07529
GmPP2C085	G	14.8195	19.2892	4.50232	19.832	2.23154	4.37569	28.3189

GmPP2C086	J	3.74477	3.6151	4.60199	3.36803	4.80455	6.84429	3.45294
GmPP2C087	F	19.4058	39.7751	26.7989	25.9298	12.9967	6.15006	20.1985
GmPP2C088	E	8.63009	5.14981	8.98832	8.1826	7.96101	33.5783	20.7444
GmPP2C089	A	13.5075	15.5451	31.6989	19.675	12.4713	18.3216	66.3628
GmPP2C090	H	2.87528	1.43947	14.655	8.82041	4.24204	3.04565	3.84501
GmPP2C091	I	8.94242	20.3547	4.94514	4.11811	4.16514	5.20776	7.20739
GmPP2C092	F	15.3274	19.0293	86.3578	34.9097	27.275	8.78449	35.3714
GmPP2C093	A	6.03906	9.62304	6.85169	9.98326	6.77689	4.4372	6.2534
GmPP2C094	F	27.9906	20.5052	42.1229	35.2503	30.6531	25.6103	35.9818
GmPP2C095	A	5.9971	1.85997	8.33853	8.92693	1.89466	12.9718	27.8711
GmPP2C096	B	1.80737	11.7698	5.91659	3.27936	2.95929	1.13444	7.4077
GmPP2C097	F	5.10817	10.7973	16.8928	5.53405	4.51454	6.97198	9.71655
GmPP2C098	J	4.82879	10.2073	4.8302	4.28025	4.14154	18.1917	2.28263
GmPP2C099	D	6.35508	7.74898	7.34724	8.68952	5.61931	5.66949	10.5217
GmPP2C100	A	2.09004	7.75268	8.7081	2.32888	0.852356	1.13524	14.5235
GmPP2C101	G	3.76461	9.0079	24.2828	4.89276	5.34477	2.34396	7.48574
GmPP2C102	D	23.0072	24.8728	22.9804	29.4603	20.8774	14.1596	18.1555
GmPP2C103	J	2.87362	3.03597	1.55697	2.96562	2.32916	3.04368	1.90772
GmPP2C104	C	6.66128	4.77821	1.77009	4.64336	7.30841	7.50817	25.5613
GmPP2C105	E	3.30055	18.8532	5.82164	4.56324	1.54166	0.175966	6.70727
GmPP2C106	D	15.1962	6.06386	7.18593	10.4262	7.45141	10.8783	4.64173
GmPP2C107	G	4.35796	6.5522	10.6761	5.26021	7.48366	3.9194	66.8078
GmPP2C108	A	15.8269	11.0162	23.4155	14.1048	15.826	8.21398	20.3015
GmPP2C109	G	22.7849	24.5066	21.5609	24.9453	7.11224	5.41254	22.1601
GmPP2C110	A	8.33707	15.7607	14.0585	16.6149	12.5731	4.87124	9.92106
GmPP2C111	F	9.63187	10.8271	37.0363	24.2564	13.3354	11.6489	35.7059
GmPP2C112	I	10.5483	22.3351	1.27266	7.84477	2.75868	10.8327	10.2794
GmPP2C113	E	0.051987	0	4.72903	0	6.83992	0	0.033086
GmPP2C114	H	2.8938	1.82801	26.6344	7.30446	4.03814	2.99203	2.65659

GmPP2C115	A	14.1429	9.75892	16.5947	13.5675	5.50374	41.9837	42.3918
GmPP2C116	B	0.007562	0	0.105033	0	0.01384	0	1.15436
GmPP2C117	C	0.192727	0	1.82566	0.009434	0.286976	0.067194	0.017876
GmPP2C118	E	0.375129	1.36466	0.498815	3.10588	20.6924	0.10936	0.65743
GmPP2C119	E	13.7049	13.4524	16.2054	10.3476	6.47028	11.2164	11.2142
GmPP2C120	E	8.70022	5.49485	10.7095	8.94023	5.24655	3.11008	8.65419
GmPP2C121	A	5.64381	1.05666	10.4314	12.4469	2.74642	11.4019	21.0782
GmPP2C122	D	2.42547	5.08305	2.55927	2.54831	3.36951	1.35525	0.518922
GmPP2C123	D	0.961906	2.86967	1.01224	1.64651	0.853364	2.44626	33.5543
GmPP2C124	H	21.7119	5.44848	15.093	21.2821	16.741	22.0539	4.09656
GmPP2C125	E	1.52657	1.39327	1.82138	12.3045	5.99011	6.92464	15.1677
GmPP2C126	D	18.1001	34.5328	63.6727	24.9056	33.5089	22.0072	17.5446
GmPP2C127	H	7.56413	3.63965	17.9945	7.56284	14.8362	7.55891	5.39592
GmPP2C128	D	2.93798	7.08123	19.7581	7.25531	4.00481	1.59698	16.2859
GmPP2C129	E	3.77884	9.99751	17.7505	3.67126	0.782989	5.92384	7.90044
GmPP2C130	H	15.9638	10.3596	33.4723	34.0987	16.8944	15.6473	12.3077
GmPP2C131	F	0.058514	0.087645	0.080355	0.085185	0.128782	0.403913	0.105683
GmPP2C132	D	2.36627	3.73664	6.19653	4.62117	6.84939	1.56565	5.55506
GmPP2C133	E	0.384329	0.792357	0.564038	1.44706	0.633013	0.928011	25.3048
GmPP2C134	F	17.1951	20.2161	14.2824	27.2176	20.2406	20.9835	23.2755
