# Supplementary material for: Pervasive duplication, biased molecular evolution and comprehensive functional analysis of the PP2C family in Glycine max
Source: BMC Genomics. 2020 Jul 6;21:465. doi: 10.1186/s12864-020-06877-4 (PMC7339511; doi:10.1186/s12864-020-06877-4)
Supplement: Supplementary file 24 — Additional file 24. PCC of the FPKM values for the duplicated GmPP2Cs pairs. [file 12864_2020_6877_MOESM24_ESM.pdf]

Additional file 24. PCC of the FPKM values for the duplicated GmPP2Cs pairs.

Duplicated gene 1	Duplicated gene 2	Subfamily	PCC_value
GmPP2C001	GmPP2C059	E	0.246013595
GmPP2C001	GmPP2C118	E	-0.123001367
GmPP2C002	GmPP2C057	J	0.951784174
GmPP2C003	GmPP2C073	G	0.537580187
GmPP2C003	GmPP2C027	G	0.23069972
GmPP2C004	GmPP2C072	D	0.782371229
GmPP2C004	GmPP2C128	D	0.066045135
GmPP2C005	GmPP2C071	A	-0.061005154
GmPP2C006	GmPP2C070	No group	0.239317902
GmPP2C007	GmPP2C060	G	0.978458527
GmPP2C007	GmPP2C107	G	0.363686957
GmPP2C008	GmPP2C102	D	0.389350679
GmPP2C011	GmPP2C055	D	0.863824563
GmPP2C011	GmPP2C122	D	0.299415798
GmPP2C012	GmPP2C096	B	0.891405894
GmPP2C012	GmPP2C076	B	0.569824548
GmPP2C012	GmPP2C075	B	0.50325206
GmPP2C012	GmPP2C116	B	0.496364968
GmPP2C013	GmPP2C115	A	0.74360231
GmPP2C013	GmPP2C077	A	0.58767729
GmPP2C013	GmPP2C089	A	0.499446407
GmPP2C014	GmPP2C123	D	0.733743517
GmPP2C014	GmPP2C061	D	0.562018018
GmPP2C014	GmPP2C084	D	0.119697302
GmPP2C015	GmPP2C124	H	0.925029085
GmPP2C015	GmPP2C062	H	0.834142855
GmPP2C015	GmPP2C130	H	0.455400126

GmPP2C016	GmPP2C129	E	0.560593557
GmPP2C016	GmPP2C063	E	0.246687656
GmPP2C016	GmPP2C105	E	0.047358554
GmPP2C016	GmPP2C125	E	-0.198831969
GmPP2C017	GmPP2C027	G	0.87666163
GmPP2C017	GmPP2C073	G	0.6074642
GmPP2C018	GmPP2C028	H	0.389372622
GmPP2C018	GmPP2C090	H	0.333948644
GmPP2C018	GmPP2C114	H	0.105793919
GmPP2C019	GmPP2C030	A	0.830098653
GmPP2C020	GmPP2C111	F	0.545294029
GmPP2C020	GmPP2C092	F	0.479491753
GmPP2C020	GmPP2C031	F	-0.33684171
GmPP2C021	GmPP2C033	G	0.68063265
GmPP2C022	GmPP2C034	F	0.923404787
GmPP2C024	GmPP2C035	No group	0.996083981
GmPP2C025	GmPP2C046	F	0.918574782
GmPP2C025	GmPP2C048	F	0.786325823
GmPP2C025	GmPP2C097	F	0.747459733
GmPP2C026	GmPP2C045	A	0.326587427
GmPP2C027	GmPP2C073	G	0.337712507
GmPP2C028	GmPP2C114	H	0.909996512
GmPP2C028	GmPP2C090	H	0.880367275
GmPP2C029	GmPP2C113	E	0.891738877
GmPP2C031	GmPP2C111	F	-0.007666585
GmPP2C031	GmPP2C092	F	-0.416401169
GmPP2C032	GmPP2C049	I	0.428792172
GmPP2C032	GmPP2C091	I	-0.281571977
GmPP2C032	GmPP2C112	I	-0.632380135

GmPP2C036	GmPP2C081	F	-0.384184692
GmPP2C037	GmPP2C088	E	-0.495748588
GmPP2C037	GmPP2C078	E	-0.818105728
GmPP2C037	GmPP2C080	E	-0.92253114
GmPP2C038	GmPP2C049	I	0.537634501
GmPP2C039	GmPP2C117	C	0.740400934
GmPP2C040	GmPP2C107	G	0.878859116
GmPP2C041	GmPP2C106	D	0.657824767
GmPP2C041	GmPP2C051	D	0.067274989
GmPP2C041	GmPP2C099	D	0.020091007
GmPP2C042	GmPP2C105	E	0.868847161
GmPP2C042	GmPP2C125	E	-0.017761626
GmPP2C043	GmPP2C104	C	0.72953058
GmPP2C043	GmPP2C052	C	0.091728635
GmPP2C044	GmPP2C086	J	-0.550789572
GmPP2C044	GmPP2C103	J	-0.620335944
GmPP2C044	GmPP2C098	J	-0.778316783
GmPP2C046	GmPP2C048	F	0.902624184
GmPP2C046	GmPP2C097	F	0.715611413
GmPP2C048	GmPP2C097	F	0.622128933
GmPP2C050	GmPP2C107	G	0.827318766
GmPP2C051	GmPP2C099	D	0.673425886
GmPP2C051	GmPP2C106	D	-0.534124888
GmPP2C052	GmPP2C104	C	0.236310132
GmPP2C053	GmPP2C100	A	0.97243342
GmPP2C053	GmPP2C083	A	0.744732506
GmPP2C053	GmPP2C108	A	0.478083446
GmPP2C054	GmPP2C101	G	0.846762495
GmPP2C054	GmPP2C109	G	0.608288536

GmPP2C054	GmPP2C085	G	-0.010712931
GmPP2C055	GmPP2C122	D	0.359349501
GmPP2C058	GmPP2C119	E	0.485855577
GmPP2C059	GmPP2C118	E	0.667726256
GmPP2C061	GmPP2C084	D	0.647338444
GmPP2C061	GmPP2C123	D	0.387132093
GmPP2C062	GmPP2C124	H	0.87170086
GmPP2C062	GmPP2C130	H	0.794905766
GmPP2C063	GmPP2C129	E	0.823188704
GmPP2C063	GmPP2C125	E	-0.419755814
GmPP2C064	GmPP2C128	D	0.943167185
GmPP2C065	GmPP2C127	H	0.833454816
GmPP2C066	GmPP2C126	D	0.968956731
GmPP2C067	GmPP2C131	F	0.690968468
GmPP2C068	GmPP2C132	D	0.541443074
GmPP2C069	GmPP2C133	E	0.494958538
GmPP2C075	GmPP2C116	B	0.999798034
GmPP2C075	GmPP2C076	B	0.987030172
GmPP2C075	GmPP2C096	B	0.403741734
GmPP2C076	GmPP2C116	B	0.985155116
GmPP2C076	GmPP2C096	B	0.435364971
GmPP2C077	GmPP2C089	A	0.862636119
GmPP2C077	GmPP2C115	A	0.833046566
GmPP2C078	GmPP2C080	E	0.578342931
GmPP2C078	GmPP2C088	E	0.219768302
GmPP2C080	GmPP2C088	E	0.730398616
GmPP2C081	GmPP2C087	F	0.635246441
GmPP2C083	GmPP2C108	A	0.820050534
GmPP2C083	GmPP2C100	A	0.813617099

GmPP2C084	GmPP2C123	D	0.024542575
GmPP2C085	GmPP2C109	G	0.77115895
GmPP2C085	GmPP2C101	G	-0.02411674
GmPP2C086	GmPP2C098	J	0.675982771
GmPP2C086	GmPP2C103	J	0.049618448
GmPP2C089	GmPP2C115	A	0.67606144
GmPP2C090	GmPP2C114	H	0.947054897
GmPP2C091	GmPP2C112	I	0.712534491
GmPP2C092	GmPP2C111	F	0.839150027
GmPP2C093	GmPP2C110	A	0.924316386
GmPP2C096	GmPP2C116	B	0.397097779
GmPP2C098	GmPP2C103	J	0.576585823
GmPP2C099	GmPP2C106	D	-0.566805667
GmPP2C100	GmPP2C108	A	0.522550535
GmPP2C101	GmPP2C109	G	0.499224908
GmPP2C124	GmPP2C130	H	0.595643293
GmPP2C125	GmPP2C129	E	-0.317347028
