# Supplementary material for: Pervasive duplication, biased molecular evolution and comprehensive functional analysis of the PP2C family in Glycine max
Source: BMC Genomics. 2020 Jul 6;21:465. doi: 10.1186/s12864-020-06877-4 (PMC7339511; doi:10.1186/s12864-020-06877-4)
Supplement: Supplementary file 25 — Additional file 25. The constructed co-expression modules in soybean by hierarchical clustering and dynamic tree cut. [file 12864_2020_6877_MOESM25_ESM.pdf]

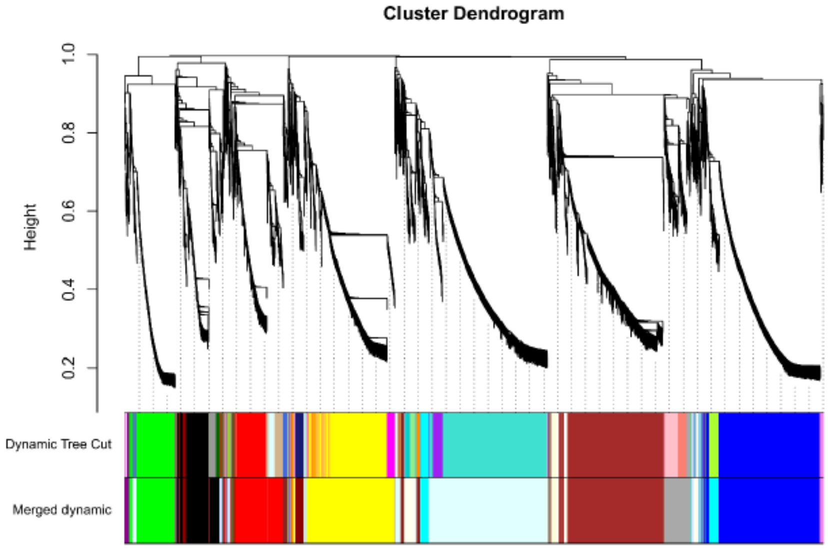

Additional file 25. The constructed co-expression modules in soybean by hierarchical clustering and dynamic tree cut.
