# Supplementary material for: Pervasive duplication, biased molecular evolution and comprehensive functional analysis of the PP2C family in Glycine max
Source: BMC Genomics. 2020 Jul 6;21:465. doi: 10.1186/s12864-020-06877-4 (PMC7339511; doi:10.1186/s12864-020-06877-4)
Supplement: Supplementary file 26 — Additional file 26. Module information of GmPP2C through WGCNA analysis. [file 12864_2020_6877_MOESM26_ESM.pdf]

Additional file 26. Module information of GmPP2C through WGCNA analysis.

Name	Subfamily	Module
GmPP2C001	E	yellow
GmPP2C002	J	lightcyan1
GmPP2C003	G	yellow
GmPP2C004	D	red
GmPP2C005	A	green
GmPP2C006	No group	brown
GmPP2C007	G	lightcyan1
GmPP2C008	D	blue
GmPP2C009	No group	lightcyan1
GmPP2C010	No group	lightyellow
GmPP2C011	D	lightcyan1
GmPP2C012	B	ivory
GmPP2C013	A	green
GmPP2C014	D	darkgrey
GmPP2C015	H	yellow
GmPP2C016	E	lightcyan1
GmPP2C017	G	ivory
GmPP2C018	H	red
GmPP2C019	A	red
GmPP2C020	F	darkgrey
GmPP2C021	G	lightcyan1
GmPP2C022	F	yellow
GmPP2C023	G	red
GmPP2C024	No group	brown
GmPP2C025	F	ivory
GmPP2C026	A	green
GmPP2C027	G	cyan

GmPP2C028	H	lightcyan1
GmPP2C029	E	lightcyan1
GmPP2C030	A	red
GmPP2C031	F	yellow
GmPP2C032	I	cyan
GmPP2C033	G	lightcyan1
GmPP2C034	F	darkred
GmPP2C035	No group	brown
GmPP2C036	F	lightsteelblue1
GmPP2C037	E	ivory
GmPP2C038	I	lightcyan1
GmPP2C039	C	lightcyan1
GmPP2C040	G	blue
GmPP2C041	D	red
GmPP2C042	E	brown
GmPP2C043	C	blue
GmPP2C044	J	blue
GmPP2C045	A	darkgrey
GmPP2C046	F	ivory
GmPP2C047	F	lightcyan1
GmPP2C048	F	lightcyan1
GmPP2C049	I	black
GmPP2C050	G	blue
GmPP2C051	D	darkred
GmPP2C052	C	lightcyan1
GmPP2C053	A	ivory
GmPP2C054	G	lightcyan1
GmPP2C055	D	brown4
GmPP2C056	No group	lightyellow

GmPP2C057	J	lightcyan1
GmPP2C058	E	brown
GmPP2C059	E	green
GmPP2C060	G	lightcyan1
GmPP2C061	D	orangered4
GmPP2C062	H	darkgrey
GmPP2C063	E	darkmagenta
GmPP2C064	D	ivory
GmPP2C065	H	darkgrey
GmPP2C066	D	lightcyan1
GmPP2C067	F	yellow
GmPP2C068	D	blue
GmPP2C069	E	ivory
GmPP2C070	No group	lightcyan1
GmPP2C071	A	yellow
GmPP2C072	D	darkolivegreen
GmPP2C073	G	lightcyan1
GmPP2C074	C	lightcyan1
GmPP2C075	B	blue
GmPP2C076	B	blue
GmPP2C077	A	blue
GmPP2C078	E	ivory
GmPP2C079	F	lightcyan1
GmPP2C080	E	darkred
GmPP2C081	F	ivory
GmPP2C082	F	lightsteelblue1
GmPP2C083	A	blue
GmPP2C084	D	yellow
GmPP2C085	G	darkgrey

GmPP2C086	J	yellow
GmPP2C087	F	brown
GmPP2C088	E	yellow
GmPP2C089	A	blue
GmPP2C090	H	lightcyan1
GmPP2C091	I	brown
GmPP2C092	F	lightcyan1
GmPP2C093	A	darkolivegreen
GmPP2C094	F	lightcyan1
GmPP2C095	A	blue
GmPP2C096	B	ivory
GmPP2C097	F	lightcyan1
GmPP2C098	J	yellow
GmPP2C099	D	blue
GmPP2C100	A	blue
GmPP2C101	G	lightcyan1
GmPP2C102	D	yellow
GmPP2C103	J	cyan
GmPP2C104	C	blue
GmPP2C105	E	brown
GmPP2C106	D	darkred
GmPP2C107	G	blue
GmPP2C108	A	cyan
GmPP2C109	G	darkorange2
GmPP2C110	A	yellow
GmPP2C111	F	cyan
GmPP2C112	I	brown
GmPP2C113	E	lightcyan1
GmPP2C114	H	lightcyan1

GmPP2C115	A	violet
GmPP2C116	B	blue
GmPP2C117	C	lightcyan1
GmPP2C118	E	green
GmPP2C119	E	green
GmPP2C120	E	yellow
GmPP2C121	A	blue
GmPP2C122	D	brown
GmPP2C123	D	blue
GmPP2C124	H	darkgrey
GmPP2C125	E	blue
GmPP2C126	D	lightcyan1
GmPP2C127	H	lightcyan1
GmPP2C128	D	cyan
GmPP2C129	E	lightcyan1
GmPP2C130	H	brown4
GmPP2C131	F	yellow
GmPP2C132	D	yellow
GmPP2C133	E	blue
GmPP2C134	F	lightcyan1
