# Supplementary material for: Pervasive duplication, biased molecular evolution and comprehensive functional analysis of the PP2C family in Glycine max
Source: BMC Genomics. 2020 Jul 6;21:465. doi: 10.1186/s12864-020-06877-4 (PMC7339511; doi:10.1186/s12864-020-06877-4)
Supplement: Supplementary file 28 — Additional file 28 Number of the responsive-regulatory elements in the promoter regions of GmPP2Cs. [file 12864_2020_6877_MOESM28_ESM.pdf]

Additional file 28. Number of the responsive-regulatory elements in the promoter regions of *GmPP2Cs*.

Name	Subfamily	ABA	Auxin	Gibberellin	Low-temperature	MeJA	SA
GmPP2C005	A	1	0	0	0	0	0
GmPP2C013	A	1	0	1	0	1	1
GmPP2C019	A	1	0	0	0	0	1
GmPP2C026	A	1	0	0	0	1	0
GmPP2C030	A	1	0	1	0	0	0
GmPP2C045	A	1	1	0	1	1	0
GmPP2C053	A	1	0	1	0	0	1
GmPP2C071	A	1	0	0	0	1	1
GmPP2C077	A	1	0	0	1	1	0
GmPP2C083	A	1	0	0	0	1	0
GmPP2C089	A	1	0	1	0	1	1
GmPP2C093	A	1	0	1	0	0	0
GmPP2C095	A	1	0	0	0	0	0
GmPP2C100	A	1	0	0	0	0	0
GmPP2C108	A	1	0	0	0	0	0
GmPP2C110	A	1	0	0	0	0	0
GmPP2C115	A	1	0	0	1	1	0
GmPP2C121	A	1	0	0	0	0	1
GmPP2C012	B	0	0	0	0	0	1
GmPP2C075	B	1	0	1	0	0	0
GmPP2C076	B	1	0	1	0	0	0
GmPP2C096	B	0	0	0	0	0	1
GmPP2C116	B	0	0	1	0	0	0
GmPP2C039	C	1	0	0	0	0	0
GmPP2C043	C	1	0	0	0	0	0
GmPP2C052	C	0	1	0	0	0	0
GmPP2C074	C	1	0	1	1	0	0
GmPP2C104	C	1	0	0	0	0	0
GmPP2C117	C	1	0	0	0	0	0
GmPP2C004	D	1	0	0	0	0	0
GmPP2C008	D	1	0	0	0	0	1
GmPP2C011	D	1	0	0	0	0	0

GmPP2C014	D	1	0	1	0	0	0
GmPP2C041	D	0	0	1	0	0	1
GmPP2C051	D	1	1	0	0	0	1
GmPP2C055	D	0	0	1	0	0	0
GmPP2C061	D	0	1	0	0	1	0
GmPP2C064	D	1	0	1	0	0	1
GmPP2C066	D	1	0	0	0	0	1
GmPP2C072	D	0	0	1	0	0	1
GmPP2C084	D	1	0	1	0	0	0
GmPP2C099	D	1	0	0	0	1	1
GmPP2C102	D	0	1	1	0	1	0
GmPP2C123	D	1	1	1	0	0	0
GmPP2C126	D	0	1	0	1	1	0
GmPP2C128	D	1	0	0	0	0	0
GmPP2C132	D	0	0	0	1	0	1
GmPP2C001	E	0	0	1	0	1	0
GmPP2C016	E	1	0	0	0	1	0
GmPP2C042	E	0	0	1	0	1	1
GmPP2C058	E	1	0	0	0	1	0
GmPP2C059	E	1	1	1	1	0	0
GmPP2C063	E	0	0	1	0	0	0
GmPP2C069	E	1	0	0	0	0	0
GmPP2C080	E	0	0	0	0	1	0
GmPP2C105	E	0	0	1	0	1	0
GmPP2C118	E	1	0	0	1	1	0
GmPP2C119	E	0	1	1	1	1	0
GmPP2C120	E	0	1	0	0	1	0
GmPP2C125	E	1	0	0	1	1	0
GmPP2C129	E	0	0	1	0	0	0
GmPP2C020	F	1	0	0	1	0	0
GmPP2C022	F	0	0	1	0	0	0
GmPP2C025	F	0	0	1	0	0	1
GmPP2C031	F	1	0	1	1	0	0
GmPP2C037	F	1	0	1	0	0	1

GmPP2C046	F	0	0	1	0	1	1
GmPP2C047	F	1	0	1	0	0	1
GmPP2C048	F	1	0	1	0	0	1
GmPP2C067	F	0	0	1	1	0	0
GmPP2C079	F	1	0	0	0	0	0
GmPP2C081	F	1	0	0	0	0	1
GmPP2C087	F	1	1	0	0	1	0
GmPP2C092	F	0	0	1	1	0	1
GmPP2C094	F	1	1	0	0	0	0
GmPP2C097	F	0	0	0	0	1	0
GmPP2C111	F	0	1	0	0	0	0
GmPP2C131	F	1	0	0	0	0	1
GmPP2C134	F	1	1	0	1	0	0
GmPP2C003	G	0	0	1	0	1	1
GmPP2C007	G	1	0	0	1	1	1
GmPP2C017	G	1	0	1	1	1	0
GmPP2C021	G	0	0	1	0	1	0
GmPP2C023	G	1	0	0	0	0	0
GmPP2C027	G	1	0	0	0	1	1
GmPP2C033	G	1	0	0	0	0	0
GmPP2C040	G	1	1	0	0	1	0
GmPP2C050	G	1	0	0	0	1	0
GmPP2C054	G	0	0	1	1	0	0
GmPP2C060	G	1	0	0	1	1	0
GmPP2C085	G	0	0	1	0	0	0
GmPP2C101	G	0	0	1	0	0	0
GmPP2C107	G	1	1	0	0	1	1
GmPP2C015	H	1	0	1	0	1	0
GmPP2C018	H	0	0	0	1	1	0
GmPP2C028	H	0	0	0	0	1	0
GmPP2C062	H	0	1	0	1	1	0
GmPP2C065	H	0	1	0	0	0	1
GmPP2C090	H	1	1	0	0	1	0
GmPP2C114	H	1	0	0	0	1	0

GmPP2C124	H	1	0	0	0	1	0
GmPP2C127	H	1	0	0	0	0	1
GmPP2C130	H	0	1	0	1	1	0
GmPP2C032	I	1	0	0	0	1	0
GmPP2C038	I	1	0	0	0	0	1
GmPP2C049	I	0	0	0	0	0	1
GmPP2C091	I	1	0	0	0	0	0
GmPP2C112	I	0	0	1	0	1	0
GmPP2C002	J	0	0	0	0	0	1
GmPP2C044	J	1	0	0	0	1	0
GmPP2C057	J	0	1	1	0	0	1
GmPP2C086	J	1	1	1	0	0	0
GmPP2C103	J	0	1	0	0	0	0
