# Supplementary material for: Pervasive duplication, biased molecular evolution and comprehensive functional analysis of the PP2C family in Glycine max
Source: BMC Genomics. 2020 Jul 6;21:465. doi: 10.1186/s12864-020-06877-4 (PMC7339511; doi:10.1186/s12864-020-06877-4)
Supplement: Supplementary file 29 — Additional file 29 Regulatory relationship between TFs and GmPP2Cs through the MERLIN+Prior method. [file 12864_2020_6877_MOESM29_ESM.pdf]

Additional file 29. Regulatory relationship between TFs and *GmPP2Cs* through the MERLIN+Prior method.

TF	Family	Target	WGCNA_module	Regression coefficient
Glyma.01G014600	HD-ZIP	GmPP2C094	lightcyan1	0.088713
Glyma.01G038600	MYB_related	GmPP2C035	brown	0.19447
Glyma.01G043300	WRKY	GmPP2C070	lightcyan1	0.100563
Glyma.01G043300	WRKY	GmPP2C126	lightcyan1	0.035813
Glyma.01G051300	NAC	GmPP2C053	ivory	0.026439
Glyma.01G051300	NAC	GmPP2C069	ivory	0.004902
Glyma.01G053800	WRKY	GmPP2C037	ivory	-0.04832
Glyma.01G053800	WRKY	GmPP2C044	blue	-0.00942
Glyma.01G053800	WRKY	GmPP2C074	lightcyan1	-0.16116
Glyma.01G064200	MIKC_MADS	GmPP2C089	blue	0.717687
Glyma.01G177200	GRAS	GmPP2C042	brown	-0.00872
Glyma.01G177200	GRAS	GmPP2C065	darkgrey	-0.44924
Glyma.01G211500	MYB	GmPP2C082	lightsteelblue1	-0.03188
Glyma.01G222200	MYB	GmPP2C041	red	0.109579
Glyma.01G224800	WRKY	GmPP2C081	ivory	-0.00713
Glyma.02G010900	WRKY	GmPP2C067	yellow	-0.03205
Glyma.02G029400	C2H2	GmPP2C039	lightcyan1	-0.04719
Glyma.02G041500	MIKC_MADS	GmPP2C096	ivory	0.073446
Glyma.02G051100	GATA	GmPP2C007	lightcyan1	-0.62673
Glyma.02G051100	GATA	GmPP2C009	lightcyan1	0.502771
Glyma.02G051100	GATA	GmPP2C012	ivory	0.101922
Glyma.02G051100	GATA	GmPP2C060	lightcyan1	-0.71149
Glyma.02G051100	GATA	GmPP2C079	lightcyan1	0.155737
Glyma.02G051100	GATA	GmPP2C101	lightcyan1	-0.6169
Glyma.02G073900	GATA	GmPP2C041	red	0.438158
Glyma.02G082000	Trihelix	GmPP2C065	darkgrey	-0.15754
Glyma.02G105900	TCP	GmPP2C004	red	0.914194

Glyma.02G105900	TCP	GmPP2C030	red	1.04358
Glyma.02G105900	TCP	GmPP2C055	brown4	0.00099
Glyma.02G109800	NAC	GmPP2C027	cyan	0.017652
Glyma.02G109800	NAC	GmPP2C092	lightcyan1	-0.00079
Glyma.02G112100	WRKY	GmPP2C025	ivory	0.785246
Glyma.02G112100	WRKY	GmPP2C100	blue	-0.13788
Glyma.02G121600	MIKC_MADS	GmPP2C133	blue	-0.11693
Glyma.02G139700	bHLH	GmPP2C092	lightcyan1	-0.09445
Glyma.02G161100	bZIP	GmPP2C030	red	0.629215
Glyma.02G234600	LBD	GmPP2C029	lightcyan1	1.68403
Glyma.02G234600	LBD	GmPP2C057	lightcyan1	-0.19745
Glyma.02G274600	EIL	GmPP2C123	blue	0.713333
Glyma.02G280000	SRS	GmPP2C061	orangered4	0.799937
Glyma.02G285900	WRKY	GmPP2C020	darkgrey	0.007495
Glyma.02G285900	WRKY	GmPP2C032	cyan	-0.05463
Glyma.02G285900	WRKY	GmPP2C033	lightcyan1	0.028871
Glyma.02G285900	WRKY	GmPP2C121	blue	-0.01092
Glyma.02G287700	MIKC_MADS	GmPP2C027	cyan	-0.0862
Glyma.02G296600	C3H	GmPP2C097	lightcyan1	-0.01398
Glyma.03G018800	TCP	GmPP2C077	blue	0.027527
Glyma.03G018800	TCP	GmPP2C115	violet	-0.00173
Glyma.03G029800	YABBY	GmPP2C083	blue	-0.09244
Glyma.03G029800	YABBY	GmPP2C107	blue	-0.10446
Glyma.03G034000	bHLH	GmPP2C086	yellow	-0.08575
Glyma.03G035200	B3	GmPP2C025	ivory	0.169673
Glyma.03G035200	B3	GmPP2C111	cyan	0.94381
Glyma.03G035200	B3	GmPP2C128	cyan	0.848724
Glyma.03G081900	MYB	GmPP2C052	lightcyan1	0.476517
Glyma.03G081900	MYB	GmPP2C112	brown	0.218655

Glyma.03G105000	bHLH	GmPP2C084	yellow	-0.34392
Glyma.03G112100	ERF	GmPP2C109	darkorange2	-0.5331
Glyma.03G135800	HSF	GmPP2C022	yellow	-0.18549
Glyma.03G164200	NAC	GmPP2C108	cyan	-0.13322
Glyma.03G170300	bHLH	GmPP2C013	green	-0.09764
Glyma.03G173300	C2H2	GmPP2C118	green	0.14442
Glyma.03G183900	MYB	GmPP2C121	blue	-0.15812
Glyma.03G203000	NF-YA	GmPP2C072	darkolivegreen	-0.3652
Glyma.03G240000	bHLH	GmPP2C045	darkgrey	-0.35897
Glyma.03G247100	bZIP	GmPP2C068	blue	0.15286
Glyma.03G250000	G2-like	GmPP2C072	darkolivegreen	-0.21329
Glyma.03G256700	WRKY	GmPP2C124	darkgrey	-0.47957
Glyma.04G027000	DBB	GmPP2C055	brown4	0.320904
Glyma.04G029600	bZIP	GmPP2C112	brown	-0.11342
Glyma.04G039300	bZIP	GmPP2C133	blue	-0.17786
Glyma.04G062900	ERF	GmPP2C013	green	0.00916
Glyma.04G062900	ERF	GmPP2C077	blue	0.036204
Glyma.04G062900	ERF	GmPP2C115	violet	-0.35139
Glyma.04G078300	bZIP	GmPP2C058	brown	-0.13289
Glyma.04G084000	ERF	GmPP2C110	yellow	0.188907
Glyma.04G182600	bHLH	GmPP2C117	lightcyan1	-0.0491
Glyma.04G205100	MYB	GmPP2C130	brown4	0.140746
Glyma.04G233300	Dof	GmPP2C055	brown4	-0.05867
Glyma.05G006100	MYB	GmPP2C089	blue	0.00912
Glyma.05G006100	MYB	GmPP2C095	blue	0.016167
Glyma.05G006100	MYB	GmPP2C099	blue	0.018663
Glyma.05G019900	TCP	GmPP2C098	yellow	1.02671
Glyma.05G019900	TCP	GmPP2C120	yellow	-0.52369
Glyma.05G050700	MIKC_MADS	GmPP2C034	darkred	0.131546

Glyma.05G051700	MYB	GmPP2C106	darkred	-0.15783
Glyma.05G063500	ERF	GmPP2C081	ivory	0.490053
Glyma.05G105600	GRAS	GmPP2C047	lightcyan1	-0.22762
Glyma.05G110600	bHLH	GmPP2C115	violet	0.06787
Glyma.05G110900	bHLH	GmPP2C112	brown	-0.5361
Glyma.05G112200	LSD	GmPP2C084	yellow	0.862669
Glyma.05G123000	WRKY	GmPP2C070	lightcyan1	-0.12597
Glyma.05G148700	MIKC_MADS	GmPP2C069	ivory	0.257247
Glyma.05G157400	ERF	GmPP2C038	lightcyan1	0.138466
Glyma.05G157400	ERF	GmPP2C047	lightcyan1	0.133098
Glyma.05G157400	ERF	GmPP2C090	lightcyan1	-0.01601
Glyma.05G160800	WRKY	GmPP2C007	lightcyan1	-0.04158
Glyma.05G160800	WRKY	GmPP2C073	lightcyan1	-0.10377
Glyma.05G162300	HSF	GmPP2C074	lightcyan1	0.390778
Glyma.05G166400	HD-ZIP	GmPP2C015	yellow	0.047292
Glyma.05G166400	HD-ZIP	GmPP2C080	darkred	0.000575
Glyma.05G166400	HD-ZIP	GmPP2C106	darkred	-0.00436
Glyma.05G166400	HD-ZIP	GmPP2C124	darkgrey	0.01206
Glyma.05G210300	TALE	GmPP2C037	ivory	-0.14467
Glyma.05G224400	C3H	GmPP2C066	lightcyan1	-0.68253
Glyma.05G224400	C3H	GmPP2C073	lightcyan1	-0.46829
Glyma.06G000400	Nin-like	GmPP2C013	green	-0.39198
Glyma.06G000400	Nin-like	GmPP2C059	green	0.601726
Glyma.06G027200	MIKC_MADS	GmPP2C075	blue	0.11618
Glyma.06G027200	MIKC_MADS	GmPP2C116	blue	0.218099
Glyma.06G040400	bZIP	GmPP2C012	ivory	-0.06424
Glyma.06G040400	bZIP	GmPP2C083	blue	0.750096
Glyma.06G042100	ERF	GmPP2C106	darkred	-0.44857
Glyma.06G045400	C2H2	GmPP2C017	ivory	-0.00823

Glyma.06G061900	WRKY	GmPP2C068	blue	-0.06324
Glyma.06G085700	ERF	GmPP2C033	lightcyan1	0.062278
Glyma.06G086600	HD-ZIP	GmPP2C078	ivory	0.180971
Glyma.06G092000	bHLH	GmPP2C118	green	0.600961
Glyma.06G160500	MYB	GmPP2C132	yellow	-0.39583
Glyma.06G289300	G2-like	GmPP2C071	yellow	0.187548
Glyma.06G290000	ERF	GmPP2C020	darkgrey	0.513258
Glyma.06G290100	C3H	GmPP2C131	yellow	0.010762
Glyma.07G016500	HD-ZIP	GmPP2C124	darkgrey	-0.21327
Glyma.07G016700	HD-ZIP	GmPP2C049	black	-0.00685
Glyma.07G018500	bHLH	GmPP2C125	blue	-1.05038
Glyma.07G038400	GRF	GmPP2C088	yellow	0.691957
Glyma.07G039400	GRAS	GmPP2C038	lightcyan1	-0.26841
Glyma.07G057400	WRKY	GmPP2C128	cyan	0.289455
Glyma.07G081300	MIKC_MADS	GmPP2C095	blue	-1.48779
Glyma.07G091600	LBD	GmPP2C012	ivory	-0.56745
Glyma.07G107500	C2H2	GmPP2C020	darkgrey	0.208651
Glyma.07G126800	C3H	GmPP2C095	blue	-0.85484
Glyma.07G135800	C2H2	GmPP2C011	lightcyan1	-0.10862
Glyma.07G135800	C2H2	GmPP2C034	darkred	-0.01139
Glyma.07G135800	C2H2	GmPP2C118	green	-0.00674
Glyma.07G249000	NF-YB	GmPP2C005	green	-0.00894
Glyma.07G262700	WRKY	GmPP2C066	lightcyan1	-0.0558
Glyma.08G017600	MYB	GmPP2C041	red	0.384311
Glyma.08G021900	WRKY	GmPP2C014	darkgrey	-0.02107
Glyma.08G029400	MYB_related	GmPP2C100	blue	-0.11982
Glyma.08G041100	CO-like	GmPP2C024	brown	-0.13725
Glyma.08G041100	CO-like	GmPP2C036	lightsteelblue1	-0.07781
Glyma.08G059900	MYB	GmPP2C012	ivory	0.141822

Glyma.08G097900	TCP	GmPP2C077	blue	-1.12698
Glyma.08G097900	TCP	GmPP2C115	violet	-0.5531
Glyma.08G118200	WRKY	GmPP2C002	lightcyan1	-0.29748
Glyma.08G137600	ERF	GmPP2C048	lightcyan1	0.662541
Glyma.08G137600	ERF	GmPP2C054	lightcyan1	1.43428
Glyma.08G149600	MYB_related	GmPP2C015	yellow	-0.34497
Glyma.08G161300	NAC	GmPP2C029	lightcyan1	-0.18006
Glyma.08G161300	NAC	GmPP2C127	lightcyan1	0.1833
Glyma.08G173400	NAC	GmPP2C022	yellow	0.399392
Glyma.08G215500	bHLH	GmPP2C106	darkred	-0.13578
Glyma.08G227700	AP2	GmPP2C067	yellow	0.103037
Glyma.08G240800	WRKY	GmPP2C012	ivory	-0.09167
Glyma.08G269800	MIKC_MADS	GmPP2C116	blue	-0.1094
Glyma.08G271000	bHLH	GmPP2C088	yellow	-0.45736
Glyma.08G303900	bHLH	GmPP2C006	brown	-0.07623
Glyma.08G303900	bHLH	GmPP2C013	green	0.004444
Glyma.08G346300	bHLH	GmPP2C013	green	0.008156
Glyma.08G365100	LBD	GmPP2C105	brown	-0.35434
Glyma.09G023600	HD-ZIP	GmPP2C018	red	0.284972
Glyma.09G029800	WRKY	GmPP2C070	lightcyan1	0.201492
Glyma.09G032100	MYB	GmPP2C083	blue	0.005493
Glyma.09G041500	ERF	GmPP2C036	lightsteelblue1	0.727956
Glyma.09G046200	NF-YB	GmPP2C113	lightcyan1	0.356125
Glyma.09G062800	GATA	GmPP2C091	brown	0.5625
Glyma.09G068700	GRF	GmPP2C072	darkolivegreen	-1.05395
Glyma.09G068700	GRF	GmPP2C093	darkolivegreen	-0.23267
Glyma.09G080000	WRKY	GmPP2C126	lightcyan1	-0.05402
Glyma.09G098300	bHLH	GmPP2C013	green	-0.0149

Glyma.09G149900	bHLH	GmPP2C021	lightcyan1	0.442406
Glyma.09G170400	BBR-BPC	GmPP2C031	yellow	1.39217
Glyma.09G170400	BBR-BPC	GmPP2C102	yellow	-0.37103
Glyma.09G170500	ZF-HD	GmPP2C088	yellow	0.188855
Glyma.09G184300	bZIP	GmPP2C003	yellow	-0.07269
Glyma.09G203000	bHLH	GmPP2C042	brown	-0.11137
Glyma.09G233800	ERF	GmPP2C125	blue	0.53002
Glyma.09G235700	NAC	GmPP2C120	yellow	-0.07276
Glyma.09G244000	WRKY	GmPP2C023	red	0.025731
Glyma.09G254800	WRKY	GmPP2C022	yellow	0.282921
Glyma.09G254800	WRKY	GmPP2C066	lightcyan1	0.366538
Glyma.09G265200	HD-ZIP	GmPP2C099	blue	-0.1274
Glyma.09G266200	MIKC_MADS	GmPP2C002	lightcyan1	0.163742
Glyma.09G274000	WRKY	GmPP2C003	yellow	-0.02742
Glyma.09G280200	WRKY	GmPP2C037	ivory	1.09591
Glyma.10G016500	ERF	GmPP2C011	lightcyan1	0.161786
Glyma.10G036600	ERF	GmPP2C074	lightcyan1	-0.05102
Glyma.10G059700	C2H2	GmPP2C016	lightcyan1	-0.08174
Glyma.10G059700	C2H2	GmPP2C114	lightcyan1	0.146853
Glyma.10G071700	bZIP	GmPP2C121	blue	0.226997
Glyma.10G161200	Trihelix	GmPP2C034	darkred	-0.01051
Glyma.10G162300	bHLH	GmPP2C134	lightcyan1	0.146078
Glyma.10G223200	ERF	GmPP2C077	blue	0.068102
Glyma.10G225100	Trihelix	GmPP2C047	lightcyan1	0.069923
Glyma.10G232000	GRAS	GmPP2C089	blue	-1.61425
Glyma.10G241300	bHLH	GmPP2C113	lightcyan1	-0.0573
Glyma.10G241400	bHLH	GmPP2C021	lightcyan1	-0.24526
Glyma.10G241400	bHLH	GmPP2C038	lightcyan1	0.409901
Glyma.10G257400	bHLH	GmPP2C114	lightcyan1	0.190171

Glyma.10G257400	bHLH	GmPP2C117	lightcyan1	0.321977
Glyma.10G269300	C3H	GmPP2C124	darkgrey	0.332695
Glyma.10G274600	ERF	GmPP2C064	ivory	-0.585
Glyma.10G280000	C2H2	GmPP2C032	cyan	-0.44372
Glyma.10G280000	C2H2	GmPP2C108	cyan	-0.43467
Glyma.10G298700	Trihelix	GmPP2C005	green	0.465499
Glyma.10G298700	Trihelix	GmPP2C026	green	-0.3097
Glyma.11G006900	CPP	GmPP2C017	ivory	-0.32538
Glyma.11G006900	CPP	GmPP2C046	ivory	-0.32388
Glyma.11G030200	MYB	GmPP2C028	lightcyan1	-0.01827
Glyma.11G045100	HD-ZIP	GmPP2C033	lightcyan1	-0.43416
Glyma.11G053100	WRKY	GmPP2C031	yellow	0.242893
Glyma.11G065000	bZIP	GmPP2C009	lightcyan1	0.157032
Glyma.11G069100	MYB_related	GmPP2C018	red	-0.06358
Glyma.11G073700	MIKC_MADS	GmPP2C089	blue	-0.21062
Glyma.11G096000	GRAS	GmPP2C028	lightcyan1	-0.84017
Glyma.11G138700	MYB_related	GmPP2C050	blue	1.23333
Glyma.11G138700	MYB_related	GmPP2C104	blue	1.68582
Glyma.11G176500	MYB	GmPP2C103	cyan	-0.06104
Glyma.11G192600	C2H2	GmPP2C032	cyan	0.020542
Glyma.11G196300	TALE	GmPP2C004	red	0.245637
Glyma.11G197900	B3	GmPP2C071	yellow	-0.44667
Glyma.11G197900	B3	GmPP2C084	yellow	0.197463
Glyma.11G236300	bZIP	GmPP2C132	yellow	0.595271
Glyma.12G016400	C3H	GmPP2C060	lightcyan1	0.343918
Glyma.12G032200	MYB	GmPP2C005	green	0.170217
Glyma.12G040600	bZIP	GmPP2C037	ivory	-0.87481
Glyma.12G040600	bZIP	GmPP2C054	lightcyan1	0.379067
Glyma.12G048500	B3	GmPP2C030	red	-0.08293

Glyma.12G048500	B3	GmPP2C088	yellow	0.315239
Glyma.12G051700	DBB	GmPP2C072	darkolivegreen	-0.00266
Glyma.12G051700	DBB	GmPP2C087	brown	0.618603
Glyma.12G051700	DBB	GmPP2C105	brown	0.097949
Glyma.12G055400	bHLH	GmPP2C012	ivory	1.25479
Glyma.12G057900	MYB	GmPP2C049	black	-0.15178
Glyma.12G066000	MYB	GmPP2C042	brown	0.324235
Glyma.12G070300	C2H2	GmPP2C010	lightyellow	0.196395
Glyma.12G079800	MYB	GmPP2C048	lightcyan1	0.139397
Glyma.12G079800	MYB	GmPP2C126	lightcyan1	-0.15706
Glyma.12G081400	C2H2	GmPP2C097	lightcyan1	0.623939
Glyma.12G088400	C2H2	GmPP2C077	blue	-0.62094
Glyma.12G089100	G2-like	GmPP2C087	brown	-0.22348
Glyma.12G100100	HD-ZIP	GmPP2C058	brown	0.000738
Glyma.12G117000	ERF	GmPP2C001	yellow	-0.04248
Glyma.12G117000	ERF	GmPP2C025	ivory	0.127725
Glyma.12G117000	ERF	GmPP2C046	ivory	0.456018
Glyma.12G171000	ARF	GmPP2C103	cyan	0.385911
Glyma.12G171600	NAC	GmPP2C050	blue	-0.17879
Glyma.12G174100	ARF	GmPP2C014	darkgrey	-0.19157
Glyma.12G184400	bZIP	GmPP2C115	violet	0.397335
Glyma.12G195200	MYB_related	GmPP2C057	lightcyan1	0.210076
Glyma.12G206900	NAC	GmPP2C041	red	-0.6243
Glyma.12G206900	NAC	GmPP2C124	darkgrey	-0.00845
Glyma.12G221500	NAC	GmPP2C025	ivory	-0.00445
Glyma.12G221500	NAC	GmPP2C033	lightcyan1	-0.01196
Glyma.12G233900	DBB	GmPP2C065	darkgrey	0.06305
Glyma.12G236800	NF-YA	GmPP2C006	brown	0.021148
Glyma.13G030900	NAC	GmPP2C114	lightcyan1	-0.15152

Glyma.13G030900	NAC	GmPP2C130	brown4	-0.68408
Glyma.13G034100	MIKC_MADS	GmPP2C123	blue	0.303117
Glyma.13G034100	MIKC_MADS	GmPP2C133	blue	0.211234
Glyma.13G052700	MIKC_MADS	GmPP2C080	darkred	0.013069
Glyma.13G096900	AP2	GmPP2C003	yellow	-0.22333
Glyma.13G103900	GATA	GmPP2C036	lightsteelblue1	0.048922
Glyma.13G103900	GATA	GmPP2C052	lightcyan1	0.013222
Glyma.13G107900	NF-YA	GmPP2C049	black	-0.0472
Glyma.13G112600	ARF	GmPP2C125	blue	1.83939
Glyma.13G117600	WRKY	GmPP2C048	lightcyan1	0.009197
Glyma.13G122900	ERF	GmPP2C008	blue	-0.092
Glyma.13G130100	bHLH	GmPP2C014	darkgrey	0.653168
Glyma.13G133100	C2H2	GmPP2C046	ivory	0.014593
Glyma.13G139000	C2H2	GmPP2C063	darkmagenta	-0.57611
Glyma.13G139000	C2H2	GmPP2C129	lightcyan1	-0.17844
Glyma.13G177500	Dof	GmPP2C051	darkred	0.169762
Glyma.13G182700	Dof	GmPP2C023	red	-0.00691
Glyma.13G197300	SRS	GmPP2C002	lightcyan1	0.025826
Glyma.13G233900	ERF	GmPP2C052	lightcyan1	-0.10894
Glyma.13G236600	ERF	GmPP2C008	blue	-0.20591
Glyma.13G249800	bHLH	GmPP2C085	darkgrey	-0.26945
Glyma.13G265000	DBB	GmPP2C091	brown	-0.30516
Glyma.13G266500	BES1	GmPP2C062	darkgrey	0.019632
Glyma.13G280000	NAC	GmPP2C011	lightcyan1	-0.47776
Glyma.13G280000	NAC	GmPP2C056	lightyellow	-0.07596
Glyma.13G289400	WRKY	GmPP2C054	lightcyan1	-0.04814
Glyma.13G291400	bHLH	GmPP2C010	lightyellow	-0.27066
Glyma.13G294300	G2-like	GmPP2C035	brown	0.573476
Glyma.13G310100	WRKY	GmPP2C046	ivory	0.086211

Glyma.13G321900	C2H2	GmPP2C022	yellow	0.026631
Glyma.13G322100	bHLH	GmPP2C038	lightcyan1	-0.24523
Glyma.13G341700	bHLH	GmPP2C099	blue	0.477646
Glyma.13G341700	bHLH	GmPP2C104	blue	-0.81863
Glyma.13G349500	C2H2	GmPP2C019	red	0.317235
Glyma.14G028900	WRKY	GmPP2C121	blue	0.807262
Glyma.14G062200	MYB	GmPP2C043	blue	0.18791
Glyma.14G062200	MYB	GmPP2C125	blue	-0.09457
Glyma.14G071400	bZIP	GmPP2C018	red	0.428676
Glyma.14G071400	bZIP	GmPP2C053	ivory	-0.35778
Glyma.14G076900	BES1	GmPP2C111	cyan	-0.32183
Glyma.14G076900	BES1	GmPP2C120	yellow	0.013255
Glyma.14G079100	B3	GmPP2C110	yellow	-0.24495
Glyma.14G088400	bHLH	GmPP2C016	lightcyan1	0.026859
Glyma.14G088400	bHLH	GmPP2C047	lightcyan1	-0.01136
Glyma.14G088400	bHLH	GmPP2C126	lightcyan1	0.057018
Glyma.14G094800	GATA	GmPP2C112	brown	0.091557
Glyma.14G102900	WRKY	GmPP2C091	brown	0.128634
Glyma.14G103100	WRKY	GmPP2C046	ivory	-0.13531
Glyma.14G135400	WRKY	GmPP2C054	lightcyan1	-0.59804
Glyma.14G135400	WRKY	GmPP2C126	lightcyan1	0.426679
Glyma.14G178000	bHLH	GmPP2C085	darkgrey	-0.98276
Glyma.14G202300	LBD	GmPP2C036	lightsteelblue1	-0.56495
Glyma.14G205600	ERF	GmPP2C029	lightcyan1	0.480075
Glyma.14G205600	ERF	GmPP2C070	lightcyan1	0.093058
Glyma.14G217200	bZIP	GmPP2C129	lightcyan1	0.399633
Glyma.15G003300	WRKY	GmPP2C106	darkred	-0.06832
Glyma.15G078300	NAC	GmPP2C027	cyan	0.474998
Glyma.15G086400	HSF	GmPP2C096	ivory	0.974147

Glyma.15G092500	TCP	GmPP2C090	lightcyan1	-0.39754
Glyma.15G110300	WRKY	GmPP2C015	yellow	-0.09901
Glyma.15G152000	ERF	GmPP2C105	brown	-0.11585
Glyma.15G166800	bHLH	GmPP2C017	ivory	0.084059
Glyma.15G176500	GRF	GmPP2C086	yellow	0.175855
Glyma.15G202200	TALE	GmPP2C109	darkorange2	-0.22069
Glyma.15G232000	bZIP	GmPP2C045	darkgrey	0.175246
Glyma.15G232000	bZIP	GmPP2C075	blue	0.213913
Glyma.15G232000	bZIP	GmPP2C076	blue	0.891291
Glyma.15G232000	bZIP	GmPP2C089	blue	0.707674
Glyma.15G232000	bZIP	GmPP2C107	blue	1.55469
Glyma.15G235000	SRS	GmPP2C024	brown	-0.21684
Glyma.15G235000	SRS	GmPP2C129	lightcyan1	-0.64168
Glyma.16G017400	MYB_related	GmPP2C042	brown	0.497338
Glyma.16G017400	MYB_related	GmPP2C087	brown	0.011307
Glyma.16G017400	MYB_related	GmPP2C105	brown	0.473484
Glyma.16G017400	MYB_related	GmPP2C109	darkorange2	0.009556
Glyma.16G017700	bHLH	GmPP2C092	lightcyan1	-0.44387
Glyma.16G021000	HD-ZIP	GmPP2C092	lightcyan1	0.405564
Glyma.16G031900	WRKY	GmPP2C074	lightcyan1	0.029246
Glyma.16G042300	GATA	GmPP2C124	darkgrey	0.071768
Glyma.16G042900	NAC	GmPP2C054	lightcyan1	-0.12185
Glyma.16G043200	NAC	GmPP2C068	blue	0.08703
Glyma.16G043200	NAC	GmPP2C069	ivory	-0.03464
Glyma.16G047600	ERF	GmPP2C040	blue	0.388023
Glyma.16G087300	bHLH	GmPP2C013	green	-0.03648
Glyma.16G154100	ERF	GmPP2C113	lightcyan1	-0.00704
Glyma.16G167500	Trihelix	GmPP2C064	ivory	0.177323
Glyma.16G201300	bHLH	GmPP2C028	lightcyan1	-0.00053

Glyma.16G217700	MYB_related	GmPP2C016	lightcyan1	0.198412
Glyma.17G010200	TALE	GmPP2C043	blue	-0.06976
Glyma.17G011400	WRKY	GmPP2C117	lightcyan1	-0.04786
Glyma.17G062600	AP2	GmPP2C030	red	-0.00246
Glyma.17G081200	MIKC_MADS	GmPP2C080	darkred	-0.11137
Glyma.17G094400	MYB_related	GmPP2C131	yellow	-0.05183
Glyma.17G096700	HD-ZIP	GmPP2C007	lightcyan1	0.131405
Glyma.17G104800	TALE	GmPP2C080	darkred	-0.7787
Glyma.17G145300	ERF	GmPP2C097	lightcyan1	-0.15321
Glyma.17G152800	ARR-B	GmPP2C058	brown	0.044456
Glyma.17G155900	bHLH	GmPP2C127	lightcyan1	0.430283
Glyma.17G157600	HD-ZIP	GmPP2C041	red	0.207791
Glyma.17G174900	HSF	GmPP2C094	lightcyan1	0.02709
Glyma.17G224800	WRKY	GmPP2C112	brown	-0.1314
Glyma.17G232600	GRF	GmPP2C071	yellow	1.40387
Glyma.17G232700	GRF	GmPP2C055	brown4	-0.27634
Glyma.17G236100	bHLH	GmPP2C066	lightcyan1	-0.0129
Glyma.18G005600	SBP	GmPP2C028	lightcyan1	-0.19492
Glyma.18G065200	MYB	GmPP2C036	lightsteelblue1	0.292424
Glyma.18G066100	C2H2	GmPP2C078	ivory	0.481339
Glyma.18G095600	MYB	GmPP2C040	blue	0.066006
Glyma.18G159900	ERF	GmPP2C081	ivory	0.288316
Glyma.18G208800	WRKY	GmPP2C060	lightcyan1	-0.00348
Glyma.18G224500	MIKC_MADS	GmPP2C001	yellow	-0.01987
Glyma.18G256500	WRKY	GmPP2C039	lightcyan1	0.242104
Glyma.18G256500	WRKY	GmPP2C113	lightcyan1	0.493678
Glyma.18G263400	WRKY	GmPP2C053	ivory	0.9969
Glyma.18G263400	WRKY	GmPP2C069	ivory	0.92336
Glyma.18G263400	WRKY	GmPP2C100	blue	1.63606

Glyma.18G278100	CO-like	GmPP2C024	brown	-0.6772
Glyma.18G281400	ERF	GmPP2C044	blue	-0.12673
Glyma.18G287200	C2H2	GmPP2C074	lightcyan1	0.379459
Glyma.18G287200	C2H2	GmPP2C079	lightcyan1	-0.10327
Glyma.18G301500	NAC	GmPP2C044	blue	0.24418
Glyma.19G021100	HD-ZIP	GmPP2C090	lightcyan1	0.225543
Glyma.19G021400	bHLH	GmPP2C034	darkred	-0.50182
Glyma.19G024500	NAC	GmPP2C095	blue	1.94726
Glyma.19G034500	MIKC_MADS	GmPP2C006	brown	0.000294
Glyma.19G047800	G2-like	GmPP2C043	blue	-0.00556
Glyma.19G047800	G2-like	GmPP2C061	orangered4	0.253953
Glyma.19G061300	MYB	GmPP2C068	blue	-0.01632
Glyma.19G061300	MYB	GmPP2C107	blue	0.021682
Glyma.19G061300	MYB	GmPP2C133	blue	0.059919
Glyma.19G061600	MYB	GmPP2C045	darkgrey	-0.00743
Glyma.19G061600	MYB	GmPP2C133	blue	0.321267
Glyma.19G108800	NAC	GmPP2C050	blue	-0.01367
Glyma.19G108800	NAC	GmPP2C076	blue	-0.05086
Glyma.19G140400	C2H2	GmPP2C032	cyan	0.372429
Glyma.19G142600	C2H2	GmPP2C043	blue	0.368549
Glyma.19G146600	G2-like	GmPP2C118	green	0.427748
Glyma.19G146600	G2-like	GmPP2C119	green	-0.23202
Glyma.19G146600	G2-like	GmPP2C120	yellow	-0.31395
Glyma.19G160900	bHLH	GmPP2C051	darkred	-0.08707
Glyma.19G217000	WRKY	GmPP2C062	darkgrey	0.06326
Glyma.19G221700	WRKY	GmPP2C029	lightcyan1	0.164247
Glyma.19G221700	WRKY	GmPP2C127	lightcyan1	-0.18679
Glyma.19G224600	MYB	GmPP2C015	yellow	0.48925
Glyma.19G224600	MYB	GmPP2C082	lightsteelblue1	0.296785

Glyma.19G234500	C2H2	GmPP2C134	lightcyan1	0.09543
Glyma.19G248900	ERF	GmPP2C091	brown	-0.02137
Glyma.19G260900	MYB_related	GmPP2C024	brown	1.6808
Glyma.19G264200	MYB	GmPP2C132	yellow	0.053472
Glyma.20G028000	WRKY	GmPP2C079	lightcyan1	2.03038
Glyma.20G070000	ERF	GmPP2C096	ivory	0.001521
Glyma.20G070100	ERF	GmPP2C062	darkgrey	-0.26427
Glyma.20G109400	C2H2	GmPP2C094	lightcyan1	-0.20812
Glyma.20G142200	HD-ZIP	GmPP2C131	yellow	-0.08283
Glyma.20G149100	CPP	GmPP2C058	brown	-0.2693
Glyma.20G149100	CPP	GmPP2C122	brown	-0.35284
Glyma.20G156800	HSF	GmPP2C016	lightcyan1	-0.68018
Glyma.20G156800	HSF	GmPP2C028	lightcyan1	-0.0485
Glyma.20G156800	HSF	GmPP2C090	lightcyan1	-0.28477
Glyma.20G156800	HSF	GmPP2C114	lightcyan1	-0.47709
Glyma.20G180100	GATA	GmPP2C006	brown	-0.11177
Glyma.20G193000	C2H2	GmPP2C094	lightcyan1	0.020004
Glyma.20G193000	C2H2	GmPP2C128	cyan	0.006885
Glyma.20G198500	NF-YB	GmPP2C057	lightcyan1	-0.09757
Glyma.20G198500	NF-YB	GmPP2C121	blue	0.532345
Glyma.20G200500	GRAS	GmPP2C098	yellow	-0.74744
Glyma.20G224000	Trihelix	GmPP2C088	yellow	-0.27537
Glyma.20G224700	bHLH	GmPP2C079	lightcyan1	0.576358
Glyma.U018000	MIKC_MADS	GmPP2C087	brown	-0.23779
Glyma.U018600	bZIP	GmPP2C056	lightyellow	-0.01959
Glyma.U028600	GRF	GmPP2C131	yellow	0.150886
Glyma.U039100	HD-ZIP	GmPP2C063	darkmagenta	-0.40235
