# Supplementary material for: Pervasive duplication, biased molecular evolution and comprehensive functional analysis of the PP2C family in Glycine max
Source: BMC Genomics. 2020 Jul 6;21:465. doi: 10.1186/s12864-020-06877-4 (PMC7339511; doi:10.1186/s12864-020-06877-4)
Supplement: Supplementary file 30 — Additional file 30 Regulatory relationship between TFs and duplicated GmPP2Cs through the MERLIN+Prior method. [file 12864_2020_6877_MOESM30_ESM.pdf]

Additional file 30. Regulatory relationship between TFs and duplicated *GmPP2Cs* through the MERLIN+Prior method.

TF	Family	Duplicated gene 1	Duplicated gene 2	Subfamily
Glyma.04G062900	ERF	GmPP2C007	GmPP2C077	A
Glyma.04G062900	ERF	GmPP2C013	GmPP2C115	A
Glyma.08G097900, Glyma.03G018800, Glyma.04G062900	TCP,TCP, ERF	GmPP2C013	GmPP2C115	A
Glyma.18G263400	WRKY	GmPP2C015	GmPP2C100	A
Glyma.06G027200	MIKC_MADS	GmPP2C075	GmPP2C116	B
Glyma.15G232000	bZIP	GmPP2C077	GmPP2C076	B
Glyma.13G139000	C2H2	GmPP2C025	GmPP2C129	E
Glyma.16G017400	MYB_related	GmPP2C063	GmPP2C105	E
Glyma.12G117000	ERF	GmPP2C075	GmPP2C046	F
Glyma.02G051100	GATA	GmPP2C042	GmPP2C060	G
Glyma.05G166400	HD-ZIP	GmPP2C053	GmPP2C124	H
Glyma.20G156800	HSF	GmPP2C028	GmPP2C090	H
Glyma.20G156800	HSF	GmPP2C028	GmPP2C114	H
Glyma.20G156800	HSF	GmPP2C090	GmPP2C114	H
