# Supplementary material for: Pervasive duplication, biased molecular evolution and comprehensive functional analysis of the PP2C family in Glycine max
Source: BMC Genomics. 2020 Jul 6;21:465. doi: 10.1186/s12864-020-06877-4 (PMC7339511; doi:10.1186/s12864-020-06877-4)
Supplement: Supplementary file 31 — Additional file 31. PCR primers used in the study. [file 12864_2020_6877_MOESM31_ESM.pdf]

Additional file 31. PCR primers used in the study.

Gene name	Forward primer (5'-3')	Reverse primer (5'-3')
GmPP2C002	ACTTGTGCTGACCGCATGTA	AGAAGCAGCACAAAACTGCG
GmPP2C057	CTGGTGAGAGGGAGGACGAT	ATCAACGGCCACAACTTCTTC
GmPP2C012	AGGTTGGACATACCCGTTTCC	GCCTCGACCAAGTCACG
GmPP2C096	TTCTGCTGCTTCTGCTTCGT	ACACCCCAAACCCATCTTCC
GmPP2C011	CACCTGCCTGAACCTATCCG	GCCTCATTTAAAGCCGCCCT
GmPP2C055	GCACAACGCTAGCAAAGAGG	GATGCCTTTAATGCGCCAGG
GmPP2C028	GGAGCCAGATGTGGTACAGG	CATCCCAGGGACGCAATCTT
GmPP2C114	CAGGAGTGATCACCAAACGG	TTCCGTCTTGAGCAGCGTC
GmPP2C025	AGAGCAGTCAGTCCTCAGTCA	GCCCTTTGAACTGCACAAAT
GmPP2C046	AGCCAATGTTGGAGATTCACG	AGATCGCAAGTGCGTTTTG
GmPP2C038	TGGACGGGTCAATGGAAGTTT	ACCGCAGAAAGTTTATTCTCAGTC
GmPP2C049	TGAGAACGACAAGCTTCGCT	TTTGCCTCCATGGCCATCAT
