# Supplementary material for: Pervasive duplication, biased molecular evolution and comprehensive functional analysis of the PP2C family in Glycine max
Source: BMC Genomics. 2020 Jul 6;21:465. doi: 10.1186/s12864-020-06877-4 (PMC7339511; doi:10.1186/s12864-020-06877-4)
Supplement: Supplementary file 12 [file 12864_2020_6877_MOESM10_ESM.docx]

Additional file 10. Locations of the conserved PP2C domain in GmPP2Cs, VvPP2Cs, AtPP2Cs, AtrPP2Cs and NcPP2Cs.

Name	Start(aa)	End (aa)	E-Value	Bit_score
AT1G03590	90	322	1.07E-41	148.648
AT1G07160	122	369	2.29E-64	205.272
AT1G07430	120	359	1.57E-70	223.377
AT1G07630	383	601	1.95E-24	102.809
AT1G09160	60	282	1.09E-34	129.003
AT1G16220	71	355	1.07E-35	132.855
AT1G17550	190	494	1.24E-64	209.895
AT1G18030	82	340	5.89E-58	187.938
AT1G22280	34	273	1.60E-56	181.775
AT1G34750	35	274	4.50E-56	181.005
AT1G43900	122	361	1.50E-68	216.058
AT1G47380	62	277	9.38E-27	107.431
AT1G48040	86	342	1.28E-67	213.747
AT1G67820	119	345	1.34E-61	200.265
AT1G68410	70	288	7.99E-38	137.477
AT1G72770	194	494	6.19E-65	210.665
AT1G78200	33	275	1.88E-59	189.479
AT1G79630	74	379	2.64E-35	132.084
AT2G20050	135	375	1.29E-32	127.077
AT2G20630	62	271	1.51E-57	184.471

AT2G25070	22	322	4.11E-80	244.948
AT2G25620	97	349	2.80E-74	231.466
AT2G28890	371	593	2.47E-24	102.424
AT2G29380	77	353	4.85E-70	219.525
AT2G30020	139	385	6.16E-59	191.79
AT2G33700	83	336	3.38E-91	274.223
AT2G34740	99	327	3.88E-58	187.938
AT2G40180	129	378	9.73E-63	201.42
AT2G40860	391	641	3.27E-82	259.585
AT3G02750	187	353	2.59E-37	137.477
AT3G05640	86	328	3.66E-41	144.796
AT3G06270	80	304	6.53E-42	146.337
AT3G09400	378	613	4.64E-24	101.653
AT3G11410	106	346	2.98E-68	216.058
AT3G12620	83	326	6.75E-37	134.01
AT3G15260	51	281	1.83E-59	189.864
AT3G16560	173	451	2.15E-35	132.084
AT3G16800	83	326	3.66E-45	154.811
AT3G17090	84	322	5.18E-35	129.003
AT3G17250	127	386	1.05E-63	204.887
AT3G51370	79	323	8.20E-39	139.018
AT3G51470	82	319	3.48E-77	237.629

AT3G55050	84	322	1.50E-35	130.543
AT3G62260	87	340	4.99E-74	230.31
AT3G63320	51	373	2.38E-36	133.625
AT3G63340	76	438	1.97E-37	140.944
AT4G03415	74	332	2.24E-39	142.485
AT4G26080	129	415	5.33E-81	249.955
AT4G27800	61	341	1.60E-50	169.834
AT4G28400	36	275	3.87E-63	199.109
AT4G31750	32	272	7.72E-69	214.517
AT4G31860	22	322	6.00E-77	236.859
AT4G32950	74	291	3.96E-44	151.344
AT4G33920	61	307	1.56E-39	140.944
AT4G38520	64	319	1.82E-41	146.337
AT5G01700	19	265	3.93E-37	133.24
AT5G02400	398	633	8.74E-23	97.8015
AT5G02760	30	292	9.85E-41	143.64
AT5G06750	82	328	2.05E-36	132.855
AT5G10740	34	272	9.59E-68	213.361
AT5G19280	309	570	2.27E-65	213.747
AT5G24940	34	272	3.41E-69	219.91
AT5G26010	75	283	2.71E-45	154.426
AT5G27930	82	333	1.25E-40	143.64

AT5G36250	68	361	6.13E-33	124.765
AT5G51760	109	372	5.75E-67	213.361
AT5G53140	102	340	1.16E-67	215.287
AT5G57050	113	404	4.60E-81	249.955
AT5G59220	112	346	6.89E-66	210.28
AT5G66080	82	326	9.84E-37	133.625
GmPP2C001	3	268	2.21E-39	137.862
GmPP2C002	147	383	2.37E-31	123.61
GmPP2C003	93	332	3.36E-73	227.999
GmPP2C004	61	306	1.48E-36	132.855
GmPP2C005	47	276	5.43E-62	197.568
GmPP2C006	299	559	1.70E-45	160.974
GmPP2C007	95	347	4.89E-72	225.688
GmPP2C008	81	319	2.45E-33	124.765
GmPP2C009	73	434	2.96E-39	146.337
GmPP2C010	393	642	7.78E-64	211.435
GmPP2C011	36	300	7.51E-39	138.633
GmPP2C012	133	378	2.55E-66	210.665
GmPP2C013	105	348	3.06E-67	213.747
GmPP2C014	71	314	5.50E-34	125.536
GmPP2C015	65	290	1.71E-32	123.225
GmPP2C016	83	314	4.08E-37	134.395

GmPP2C017	94	313	1.89E-66	209.124
GmPP2C018	58	288	3.51E-27	108.587
GmPP2C019	258	527	1.58E-69	223.762
GmPP2C020	33	273	1.93E-69	216.058
GmPP2C021	70	326	8.64E-75	231.851
GmPP2C022	53	293	6.22E-64	201.805
GmPP2C023	1	120	2.21E-43	143.64
GmPP2C024	59	342	3.21E-49	166.367
GmPP2C025	34	273	1.08E-62	197.953
GmPP2C026	93	363	6.48E-65	206.813
GmPP2C027	91	344	2.24E-84	256.889
GmPP2C028	58	288	3.12E-28	111.669
GmPP2C029	65	313	2.46E-41	144.796
GmPP2C030	258	527	1.36E-67	218.754
GmPP2C031	33	273	4.99E-71	220.295
GmPP2C032	22	322	1.77E-78	240.325
GmPP2C033	79	326	5.07E-76	234.933
GmPP2C034	46	285	3.43E-64	202.191
GmPP2C035	61	344	8.51E-48	162.9
GmPP2C036	35	274	6.39E-54	175.227
GmPP2C037	73	347	1.04E-36	135.166
GmPP2C038	27	326	2.66E-81	248.415

GmPP2C039	361	562	8.51E-25	103.579
GmPP2C040	89	341	2.49E-70	221.065
GmPP2C041	76	315	7.61E-35	128.232
GmPP2C042	91	330	2.09E-44	153.27
GmPP2C043	261	475	3.59E-25	103.965
GmPP2C044	82	323	8.87E-40	142.485
GmPP2C045	94	364	5.96E-67	212.206
GmPP2C046	34	273	6.36E-63	198.339
GmPP2C047	117	344	3.20E-67	211.821
GmPP2C048	32	271	2.28E-60	191.79
GmPP2C049	27	326	1.69E-79	243.792
GmPP2C050	108	360	5.51E-71	223.377
GmPP2C051	75	315	1.28E-33	125.151
GmPP2C052	132	364	2.60E-28	111.669
GmPP2C053	208	505	1.83E-74	235.703
GmPP2C054	96	340	1.76E-74	231.466
GmPP2C055	73	319	7.66E-38	136.707
GmPP2C056	57	314	7.07E-55	179.079
GmPP2C057	126	365	7.71E-32	124.765
GmPP2C058	70	342	4.56E-38	139.403
GmPP2C059	74	329	3.90E-42	149.033
GmPP2C060	95	347	1.02E-72	227.614

GmPP2C061	48	315	6.26E-39	139.018
GmPP2C062	66	290	1.48E-34	128.617
GmPP2C063	83	333	7.84E-43	149.418
GmPP2C064	11	300	5.49E-40	142.099
GmPP2C065	60	290	5.64E-34	127.077
GmPP2C066	81	325	6.22E-37	134.395
GmPP2C067	71	310	1.02E-68	214.517
GmPP2C068	67	321	9.06E-38	136.321
GmPP2C069	45	183	8.82E-31	112.824
GmPP2C070	299	557	5.12E-50	172.915
GmPP2C071	60	288	9.99E-58	186.783
GmPP2C072	61	304	1.95E-38	137.862
GmPP2C073	79	332	6.16E-74	229.925
GmPP2C074	442	680	2.31E-24	102.424
GmPP2C075	80	324	9.50E-61	194.487
GmPP2C076	81	325	7.16E-61	194.872
GmPP2C077	108	340	2.78E-67	213.747
GmPP2C078	73	347	4.46E-38	139.018
GmPP2C079	34	273	1.12E-61	195.257
GmPP2C080	92	347	1.07E-35	132.469
GmPP2C081	61	257	1.82E-42	145.181
GmPP2C082	90	328	4.40E-69	216.828

GmPP2C083	217	519	6.97E-67	216.443
GmPP2C084	48	315	7.87E-38	136.321
GmPP2C085	95	342	2.66E-71	223.377
GmPP2C086	82	321	5.37E-40	143.255
GmPP2C087	61	275	1.46E-55	179.464
GmPP2C088	73	347	1.25E-36	135.166
GmPP2C089	105	347	3.57E-66	210.665
GmPP2C090	56	286	2.83E-27	108.972
GmPP2C091	22	311	4.44E-74	229.155
GmPP2C092	78	318	6.07E-70	218.369
GmPP2C093	243	540	6.49E-73	233.007
GmPP2C094	90	328	7.00E-69	216.443
GmPP2C095	117	372	6.33E-50	168.293
GmPP2C096	134	379	3.60E-68	215.673
GmPP2C097	30	269	1.43E-58	187.168
GmPP2C098	82	321	3.89E-40	143.64
GmPP2C099	74	313	3.31E-33	123.995
GmPP2C100	215	510	6.68E-75	237.244
GmPP2C101	94	338	1.36E-71	224.147
GmPP2C102	81	319	3.27E-35	129.773
GmPP2C103	82	323	1.48E-37	138.247
GmPP2C104	270	479	4.65E-25	103.965

GmPP2C105	99	338	2.75E-43	150.574
GmPP2C106	77	316	2.98E-34	126.691
GmPP2C107	83	335	1.50E-69	218.754
GmPP2C108	219	521	3.66E-67	217.213
GmPP2C109	95	342	4.40E-70	220.295
GmPP2C110	244	541	2.86E-71	228.384
GmPP2C111	78	318	7.21E-70	217.984
GmPP2C112	39	328	9.51E-76	233.777
GmPP2C113	62	292	9.25E-39	137.862
GmPP2C114	56	286	1.70E-25	103.965
GmPP2C115	107	343	1.19E-67	214.517
GmPP2C116	92	336	1.54E-64	204.887
GmPP2C117	343	557	2.07E-28	113.98
GmPP2C118	74	329	8.27E-42	147.877
GmPP2C119	70	342	3.60E-40	144.796
GmPP2C120	102	390	7.70E-40	143.255
GmPP2C121	107	363	2.31E-50	169.063
GmPP2C122	79	324	2.69E-40	143.255
GmPP2C123	71	314	5.20E-34	125.921
GmPP2C124	65	290	4.69E-36	132.855
GmPP2C125	83	331	7.36E-40	141.329
GmPP2C126	81	325	1.16E-36	133.625

GmPP2C127	60	290	3.04E-33	125.151
GmPP2C128	63	303	2.30E-40	142.87
GmPP2C129	83	333	1.24E-39	140.944
GmPP2C130	66	290	6.94E-34	127.077
GmPP2C131	1	233	1.14E-54	175.612
GmPP2C132	66	320	5.52E-39	139.788
GmPP2C133	55	308	4.01E-42	147.492
GmPP2C134	35	274	1.38E-55	179.464
VvPP2C01	63	311	1.18E-35	130.543
VvPP2C02	61	274	1.56E-58	191.405
VvPP2C03	93	352	4.00E-28	110.898
VvPP2C04	64	354	9.95E-49	164.441
VvPP2C05	70	429	1.17E-42	155.967
VvPP2C06	101	340	2.91E-59	192.946
VvPP2C07	8	185	1.30E-30	113.98
VvPP2C08	109	367	5.80E-61	196.798
VvPP2C09	35	274	1.67E-59	189.479
VvPP2C10	60	307	1.10E-39	141.329
VvPP2C11	56	277	3.46E-26	105.891
VvPP2C12	27	266	5.11E-66	206.043
VvPP2C13	59	289	1.51E-25	104.35
VvPP2C14	70	314	3.95E-69	216.828

VvPP2C15	64	317	1.42E-82	251.496
VvPP2C16	66	275	7.91E-57	182.931
VvPP2C17	30	307	2.34E-38	139.018
VvPP2C18	55	349	1.05E-71	223.762
VvPP2C19	38	313	8.38E-69	215.287
VvPP2C20	125	362	2.35E-33	129.388
VvPP2C21	1	292	1.99E-74	229.54
VvPP2C22	23	259	3.65E-66	206.043
VvPP2C23	202	488	2.00E-69	222.221
VvPP2C24	84	321	6.40E-61	196.413
VvPP2C25	84	321	6.47E-62	198.724
VvPP2C26	56	309	5.78E-61	195.642
VvPP2C27	178	435	1.07E-27	110.898
VvPP2C28	304	564	2.25E-37	139.018
VvPP2C29	60	310	3.93E-59	191.79
VvPP2C30	84	341	2.78E-41	147.492
VvPP2C31	102	367	2.38E-66	211.05
VvPP2C32	145	307	1.30E-52	173.686
VvPP2C33	47	275	1.19E-61	195.257
VvPP2C34	79	318	6.21E-80	245.718
VvPP2C35	81	325	2.13E-34	127.847
VvPP2C36	1	232	1.16E-63	198.724

VvPP2C37	114	382	1.19E-68	216.828
VvPP2C38	350	571	1.73E-24	102.809
VvPP2C39	76	314	1.18E-36	133.24
VvPP2C40	394	642	9.52E-71	229.54
VvPP2C41	95	347	2.02E-63	203.346
VvPP2C42	123	278	1.62E-48	161.745
VvPP2C43	91	329	2.32E-68	214.902
VvPP2C44	29	236	5.45E-41	149.803
VvPP2C45	73	348	1.59E-37	137.092
VvPP2C46	91	332	1.84E-42	148.263
VvPP2C47	65	290	5.72E-33	124.38
VvPP2C48	60	320	1.23E-34	128.232
VvPP2C49	1	232	1.81E-63	198.339
VvPP2C50	77	320	4.98E-39	139.788
VvPP2C51	49	277	1.42E-59	189.864
VvPP2C52	72	317	2.26E-33	124.38
VvPP2C53	162	400	8.63E-46	159.048
VvPP2C54	63	270	9.38E-43	147.877
VvPP2C55	192	488	5.33E-72	229.155
VvPP2C56	88	335	3.62E-76	235.703
VvPP2C57	23	323	5.97E-76	234.547
VvPP2C58	32	272	1.34E-71	221.065

VvPP2C59	60	303	1.36E-57	186.012
VvPP2C60	61	301	1.33E-39	140.944
VvPP2C61	178	457	3.36E-54	182.545
VvPP2C62	1	227	1.03E-64	201.035
AtrPP2C01	82	326	2.11E-39	140.944
AtrPP2C02	39	388	1.50E-41	152.885
AtrPP2C03	99	353	3.67E-59	192.561
AtrPP2C04	67	305	7.18E-35	128.232
AtrPP2C05	119	322	2.06E-53	175.612
AtrPP2C06	118	403	4.07E-65	208.354
AtrPP2C07	116	359	1.50E-63	204.887
AtrPP2C08	270	482	4.61E-27	109.357
AtrPP2C09	27	264	8.88E-67	207.969
AtrPP2C10	31	271	7.79E-70	216.058
AtrPP2C11	79	322	4.99E-41	145.181
AtrPP2C12	38	301	8.24E-41	144.796
AtrPP2C13	91	330	2.65E-38	137.092
AtrPP2C14	56	284	2.59E-58	186.783
AtrPP2C15	247	508	1.01E-38	141.714
AtrPP2C16	48	297	8.20E-48	161.359
AtrPP2C17	35	265	1.14E-60	192.175
AtrPP2C18	123	405	3.10E-67	214.132

AtrPP2C19	104	344	2.81E-78	241.481
AtrPP2C20	130	386	3.82E-58	189.864
AtrPP2C21	72	309	1.86E-40	143.64
AtrPP2C22	247	470	5.42E-24	100.498
AtrPP2C23	388	637	1.43E-69	226.458
AtrPP2C24	221	521	4.99E-59	196.027
AtrPP2C25	74	302	7.56E-39	139.788
AtrPP2C26	87	338	4.82E-42	147.877
AtrPP2C27	105	382	8.47E-51	171.76
AtrPP2C28	84	374	3.90E-44	156.352
AtrPP2C29	115	360	1.02E-63	203.346
AtrPP2C30	1	135	2.21E-36	125.151
AtrPP2C31	120	334	2.60E-58	189.094
AtrPP2C32	64	294	7.61E-37	134.781
AtrPP2C33	31	271	2.76E-68	212.206
AtrPP2C34	104	344	5.39E-61	196.027
AtrPP2C35	124	361	3.51E-33	128.617
AtrPP2C36	89	356	3.82E-68	216.058
AtrPP2C37	54	270	8.37E-32	121.299
AtrPP2C38	24	322	2.84E-76	234.547
AtrPP2C39	225	508	7.50E-72	229.155
AtrPP2C40	66	356	6.12E-43	152.115

AtrPP2C41	66	275	3.87E-59	188.709
AtrPP2C42	96	334	7.23E-68	213.747
AtrPP2C43	2	148	1.29E-48	158.278
NcPP2C01	69	303	1.41E-60	194.872
NcPP2C02	161	374	8.56E-48	162.515
NcPP2C03	48	324	2.99E-63	201.42
NcPP2C04	34	151	7.38E-36	124.765
NcPP2C05	62	275	2.49E-57	184.086
NcPP2C06	116	369	4.69E-32	125.536
NcPP2C07	97	336	2.09E-38	139.403
NcPP2C08	92	332	4.68E-67	211.435
NcPP2C09	65	322	1.22E-30	117.061
NcPP2C10	92	324	9.25E-37	133.625
NcPP2C11	47	276	2.00E-57	184.471
NcPP2C12	64	285	2.26E-32	122.839
NcPP2C13	263	511	6.71E-80	249.955
NcPP2C14	68	306	1.75E-43	151.344
NcPP2C15	57	285	3.08E-55	179.079
NcPP2C16	70	324	4.44E-38	137.477
NcPP2C17	72	314	2.06E-39	140.944
NcPP2C18	202	441	9.81E-77	240.711
NcPP2C19	235	470	1.46E-26	108.202

NcPP2C20	87	326	3.47E-40	143.255
NcPP2C21	120	391	1.47E-37	139.403
NcPP2C22	108	348	1.04E-50	171.76
NcPP2C23	31	265	8.88E-70	215.673
NcPP2C24	36	242	6.62E-63	197.953
NcPP2C25	24	375	1.06E-56	186.397
NcPP2C26	74	329	2.62E-36	132.469
NcPP2C27	60	316	1.42E-39	142.099
NcPP2C28	261	546	4.21E-59	196.798
NcPP2C29	815	1021	4.17E-42	154.426
NcPP2C29	542	589	1.81E-11	65.4447
NcPP2C30	121	369	1.95E-62	201.805
NcPP2C31	126	367	8.85E-57	185.627
NcPP2C32	133	353	8.66E-26	105.505
NcPP2C33	67	300	4.70E-55	191.02
NcPP2C34	57	307	1.42E-47	160.204
NcPP2C35	72	314	9.96E-41	144.025
NcPP2C36	104	343	3.53E-79	243.792
NcPP2C37	335	596	1.80E-40	147.877
NcPP2C38	86	327	9.94E-38	135.936
NcPP2C39	90	328	2.50E-35	130.158
NcPP2C40	23	143	1.58E-40	136.707
