# Supplementary material for: Pervasive duplication, biased molecular evolution and comprehensive functional analysis of the PP2C family in Glycine max
Source: BMC Genomics. 2020 Jul 6;21:465. doi: 10.1186/s12864-020-06877-4 (PMC7339511; doi:10.1186/s12864-020-06877-4)
Supplement: Supplementary file 13 [file 12864_2020_6877_MOESM11_ESM.docx]

Additional file 11. Motif information of GmPP2Cs through the MEME tool.

Motif_ID	Motif_sequence	Length	Number_sites	CDD_annotation
MEME-1	LTPDDEFLILASDGLWDVLSNZEAVDIVR	29	134	PP2C
MEME-2	LYVANVGDSRAVLCR	15	131	PP2C
MEME-3	TFFGVFDGHGGPGAA	15	132	PP2C
MEME-4	GGLAVSRAIGDRYLK	15	134	PP2C
MEME-5	NNPRNGIAKRLVKAALQEAAKKREMRYS<br>DLQKIDKGVRRHFHDDITVIVVFLBHNLI	57	27	PP2C
MEME-6	AIQLSVDHKPNREDE	15	132	PP2C
MEME-7	LVEEALSRGSKDBITVIVVDF	21	86	PP2C
MEME-8	GSSSGSTAVTAIIQG	15	133	-
MEME-9	PTLRSGSSSVIGRRREMEDAHIVIPDLVQ	29	54	PP2C
MEME-10	GRKDGLLWYKDLGKHLSGEF<br>SMAVVQANNSLEDQSQIESGP	41	22	-
MEME-11	AJKKAFLKTDEEFLS	15	130	-
MEME-12	PTRSSAARRLVESAVRAWKR<br>KYPGSKVDDCAVVCLFLHSKS	41	18	-
MEME-13	RIEAAGGRVIN	11	107	-
MEME-14	GZFNREPLGAKFRLPEPFCQPVLSDEPSI	29	45	-
MEME-15	RFVRDHLFSNLLEEI	15	122	-
MEME-16	GVIAEPEVTVV	11	113	-
MEME-17	RSLHPBDPQIVVLKHGVWRVK	21	23	-

MEME-18	GSSRFASVFTQQGQKGVNQDAMIVWEEFG	29	22	-
MEME-19	RKKSSESSSKIDKKLSAVDVVEE<br>LFEEGSAMLSERLGKDYPLNNN	45	10	-
MEME-20	FGIFRCAVCQVDIPPGEGISIHEGSPFSPK<br>SKPWEGPFLCSSCQEKKDAMEGKRSSG	57	12	-
