# Supplementary material for: Pervasive duplication, biased molecular evolution and comprehensive functional analysis of the PP2C family in Glycine max
Source: BMC Genomics. 2020 Jul 6;21:465. doi: 10.1186/s12864-020-06877-4 (PMC7339511; doi:10.1186/s12864-020-06877-4)
Supplement: Supplementary file 14 [file 12864_2020_6877_MOESM12_ESM.docx]

Additional file 12. Motif distributions in the GmPP2C subfamily.

Subfamily	A	B	C	D	E	F	G	H	I	J
Total_Member	18	5	6	21	20	20	16	10	5	6
Motif-1	18	5	6	21	20	20	16	10	5	6
Motif-2	18	5	6	21	20	18	15	10	5	6
Motif-3	18	5	6	21	19	20	15	10	5	6
Motif-4	18	5	6	21	20	20	16	10	5	6
Motif-5	0	0	6	21	0	0	0	0	0	0
Motif-6	18	5	6	21	20	20	14	10	5	6
Motif-7	18	5	0	0	0	20	15	10	5	6
Motif-8	18	5	6	21	20	20	15	10	5	6
Motif-9	18	5	0	0	0	7	15	0	5	0
Motif-10	0	0	0	20	0	1	1	0	0	0
Motif-11	18	5	6	21	19	20	15	10	5	6
Motif-12	0	0	0	0	18	0	0	0	0	0
Motif-13	18	5	0	0	20	20	16	10	5	6
Motif-14	0	2	0	21	0	12	0	10	0	0
Motif-15	18	5	6	21	13	20	15	10	5	6
Motif-16	18	5	6	0	20	20	16	10	5	6
Motif-17	0	0	0	21	0	0	0	2	0	0
Motif-18	0	0	2	0	18	0	0	0	0	1
Motif-19	0	0	0	0	0	0	0	10	0	0
Motif-20	0	0	0	0	0	0	0	10	0	0
