# Supplementary material for: Pervasive duplication, biased molecular evolution and comprehensive functional analysis of the PP2C family in Glycine max
Source: BMC Genomics. 2020 Jul 6;21:465. doi: 10.1186/s12864-020-06877-4 (PMC7339511; doi:10.1186/s12864-020-06877-4)
Supplement: Supplementary file 15 [file 12864_2020_6877_MOESM13_ESM.docx]

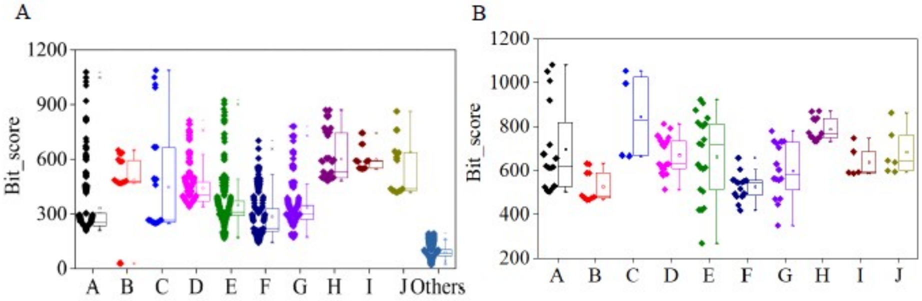

Additional file 13. Sequence similarity of GmPP2Cs. (A) Sequence similarity of GmPP2Cs from the same and different subfamily. (B) Sequence similarity of duplicated GmPP2Cs in each subfamily.
