# Supplementary material for: Pervasive duplication, biased molecular evolution and comprehensive functional analysis of the PP2C family in Glycine max
Source: BMC Genomics. 2020 Jul 6;21:465. doi: 10.1186/s12864-020-06877-4 (PMC7339511; doi:10.1186/s12864-020-06877-4)
Supplement: Supplementary file 16 [file 12864_2020_6877_MOESM14_ESM.docx]

Additional file 14. Structural analysis of GmPP2C in this study.

Name	Subfamily	Extron number	Intron number	Molecular weight	Theoretical pI	Protein length (aa)	α-helix	Extended strand	β-turn	Random coil
GmPP2C001	E	4	3	32192.77	6.65	290	101	60	33	96
GmPP2C002	J	17	16	121815.63	5.26	1096	437	213	88	358
GmPP2C003	G	4	3	40365.95	5.42	374	118	76	40	140
GmPP2C004	D	4	3	41796.69	6.43	377	142	85	26	124
GmPP2C005	A	3	2	35093.05	6.75	324	102	78	26	118
GmPP2C006	No group	12	11	64859.91	6.15	578	197	123	41	217
GmPP2C007	G	4	3	43461.91	4.78	396	169	62	24	141
GmPP2C008	D	4	3	43795.09	8.79	394	147	85	40	122
GmPP2C009	No group	20	19	121060.67	5.77	1073	388	200	82	403
GmPP2C010	No group	12	11	73123.78	6.08	659	238	117	69	235
GmPP2C011	D	5	4	41134.22	9.25	366	142	82	36	106
GmPP2C012	B	4	3	41812.92	6.28	389	104	89	44	152
GmPP2C013	A	4	3	44933.13	6.67	407	144	88	29	146
GmPP2C014	D	4	3	39901.23	6.84	357	126	63	48	120
GmPP2C015	H	7	4	45561.23	7.98	426	116	113	39	158
GmPP2C016	E	5	4	41097.8	6.64	371	129	93	48	101
GmPP2C017	G	5	4	36106.99	5.21	330	86	78	40	126
GmPP2C018	H	9	8	46556.83	5.28	429	143	92	44	150
GmPP2C019	A	4	3	59216.9	4.86	544	163	125	49	207
GmPP2C020	F	8	7	33540.32	5.02	312	113	67	35	97
GmPP2C021	G	4	3	40592.64	5.29	370	143	56	35	136
GmPP2C022	F	4	3	33228.71	7.7	301	96	66	28	111
GmPP2C023	G	1	0	18046.72	5.64	161	62	29	13	57
GmPP2C024	No group	11	10	42857.71	5.76	390	146	86	38	120
GmPP2C025	F	5	4	30673.49	6.66	282	85	60	36	101
GmPP2C026	A	4	3	42155.3	5.36	384	139	82	32	131
GmPP2C027	G	4	3	41908.8	5.28	385	113	85	41	146
GmPP2C028	H	9	8	46694.27	5.29	429	133	97	44	155
GmPP2C029	E	5	4	37342.22	5.35	343	104	86	41	112
GmPP2C030	A	4	3	58934.53	4.83	544	153	141	47	203
GmPP2C031	F	8	7	33766.48	5.18	314	124	59	32	99
GmPP2C032	I	10	9	38079.8	5.07	345	147	65	26	107

GmPP2C033	G	4	3	40595.79	5.3	370	158	50	36	126
GmPP2C034	F	5	4	32415.53	7.09	293	102	58	25	108
GmPP2C035	No group	11	10	43150.91	5.84	392	142	90	44	116
GmPP2C036	F	5	4	30849.97	5.87	282	107	59	33	83
GmPP2C037	E	7	6	33066.14	6.57	294	120	74	32	68
GmPP2C038	I	11	10	40468.59	5.24	368	150	68	27	123
GmPP2C039	C	4	3	66123.01	5.13	602	184	148	66	204
GmPP2C040	G	2	1	42549.56	5.04	386	160	76	38	112
GmPP2C041	D	4	3	41517.38	7.81	374	119	96	38	121
GmPP2C042	E	5	4	41031.05	8.29	367	123	98	40	106
GmPP2C043	C	4	3	56834.23	4.99	520	177	112	51	180
GmPP2C044	J	3	2	46088.2	5.05	423	133	98	42	150
GmPP2C045	A	4	3	42285.55	5.26	385	127	83	33	142
GmPP2C046	F	5	4	30694.42	6.17	282	86	61	31	104
GmPP2C047	F	6	5	39455.77	4.86	351	145	62	37	107
GmPP2C048	F	5	4	30455.45	6.25	280	82	73	37	88
GmPP2C049	I	11	10	40417.57	5.17	368	155	68	27	118

GmPP2C050	G	2	1	44584.63	5.01	405	171	68	29	137
GmPP2C051	D	4	3	41362.15	7.25	374	143	83	32	116
GmPP2C052	C	3	2	45167.96	4.84	409	148	88	41	132
GmPP2C053	A	4	3	57118.04	5.42	522	156	94	55	217
GmPP2C054	G	4	3	42730.21	5.04	381	147	77	32	125
GmPP2C055	D	4	3	43080.32	8.71	385	145	85	45	110
GmPP2C056	No group	10	9	35477.22	6.76	325	135	67	34	89
GmPP2C057	J	16	15	119303.73	5.02	1075	424	204	100	347
GmPP2C058	E	5	4	54209.69	5.53	489	168	94	39	188
GmPP2C059	E	5	4	48642.41	5.11	434	147	98	47	142
GmPP2C060	G	4	3	43572.14	4.76	396	181	57	29	129
GmPP2C061	D	4	3	40953.34	6.43	371	146	64	28	133
GmPP2C062	H	7	6	45996.65	7.49	428	109	111	39	169
GmPP2C063	E	5	4	41126.43	5.78	368	119	94	47	108
GmPP2C064	D	4	3	42452.47	8.13	378	128	94	35	121
GmPP2C065	H	8	7	46614.22	7.99	431	131	106	48	146
GmPP2C066	D	4	3	43516.33	7.28	397	156	82	35	124
GmPP2C067	F	8	7	34490.27	5.02	320	100	82	25	113
GmPP2C068	D	4	3	43106.25	8.1	389	160	81	40	108
GmPP2C069	E	3	2	22659.85	5.57	208	43	68	28	69
GmPP2C070	No group	12	11	64564.45	5.56	576	205	125	45	201
GmPP2C071	A	3	2	36484.61	7.89	336	116	75	37	108
GmPP2C072	D	4	3	41826.79	5.83	375	142	83	29	121
GmPP2C073	G	4	3	40313.84	5.51	374	133	67	43	131
GmPP2C074	C	4	3	79617.51	5.53	720	257	138	61	264
GmPP2C075	B	4	3	36549.43	6.71	335	87	86	43	119
GmPP2C076	B	4	3	36663.53	6.71	336	87	86	41	122
GmPP2C077	A	4	3	43834.33	5.51	401	100	110	38	153
GmPP2C078	E	5	4	51711.72	5.3	471	118	103	43	207
GmPP2C079	F	5	4	31172.5	6.07	281	107	67	35	72
GmPP2C080	E	5	4	51766.93	5.04	474	109	117	39	209
GmPP2C081	F	6	5	29350.15	6.51	265	106	51	29	79
GmPP2C082	F	8	7	39260.01	5.17	356	120	85	44	107
GmPP2C083	A	4	3	58563.88	5.07	536	221	95	31	189

GmPP2C084	D	4	3	40947.29	6.47	371	147	64	29	131
GmPP2C085	G	4	3	42454.17	5.18	383	158	73	38	114
GmPP2C086	J	3	2	46931.35	5.12	431	112	117	33	169
GmPP2C087	F	5	4	31107.25	7.7	283	108	61	27	87
GmPP2C088	E	5	4	52153.36	5.31	475	127	107	43	198
GmPP2C089	A	4	3	44150.19	6.05	400	126	90	25	159
GmPP2C090	H	9	8	46492.9	5.29	428	113	94	40	181
GmPP2C091	I	10	9	37413.39	5.37	339	134	77	35	93
GmPP2C092	F	8	7	36587.85	5.15	338	144	59	28	107
GmPP2C093	A	4	3	60815.6	4.62	557	196	112	47	202
GmPP2C094	F	8	7	39293.99	5.47	356	138	76	43	99
GmPP2C095	A	3	2	42074.51	6.73	386	140	104	35	107
GmPP2C096	B	4	3	41811.95	6.11	390	99	93	43	155
GmPP2C097	F	5	4	30487.53	6.31	278	99	65	36	78
GmPP2C098	J	3	2	46720.17	5.18	431	109	118	36	168
GmPP2C099	D	4	3	41084.92	7.69	372	134	85	37	116
GmPP2C100	A	4	3	57849.7	5.22	527	176	102	54	195
GmPP2C101	G	4	3	42328	5.46	379	136	91	34	118
GmPP2C102	D	4	3	43876.13	8.79	394	144	92	39	119
GmPP2C103	J	6	5	54896.25	4.84	498	136	121	52	189
GmPP2C104	C	3	2	57138.86	5.52	524	176	119	62	167
GmPP2C105	E	6	5	41908.18	7.99	376	128	97	43	108
GmPP2C106	D	4	3	41437.23	7.8	375	129	93	41	112
GmPP2C107	G	2	1	41708.68	5.09	380	153	75	37	115
GmPP2C108	A	4	3	58548.81	4.97	538	213	98	34	193
GmPP2C109	G	4	3	42290.77	5.11	383	170	65	41	107
GmPP2C110	A	4	3	60792.54	4.62	558	169	129	49	211
GmPP2C111	F	8	7	36655.97	5.09	338	145	58	31	104
GmPP2C112	I	11	10	39528.9	5.35	356	135	90	40	91
GmPP2C113	E	5	4	37627.48	5.07	344	138	73	43	90
GmPP2C114	H	9	8	46575.99	5.23	428	123	87	38	180
GmPP2C115	A	4	3	44067.66	5.17	400	104	106	33	157
GmPP2C116	B	4	3	37640.64	5.81	347	77	94	38	138
GmPP2C117	C	4	3	65974.13	5.37	597	182	157	67	191

GmPP2C118	E	5	4	47458.29	5.24	424	127	102	56	139
GmPP2C119	E	5	4	54026.4	6.1	487	150	101	44	192
GmPP2C120	E	5	4	50115.64	5.3	455	138	86	51	180
GmPP2C121	A	3	2	40769.73	5.68	377	132	89	36	120
GmPP2C122	D	4	3	44150.56	7.69	391	167	81	41	102
GmPP2C123	D	4	3	41129.64	8.22	370	120	74	47	129
GmPP2C124	H	7	6	46091	7.97	429	117	116	35	161
GmPP2C125	E	5	4	40812.38	6.09	369	143	93	47	86
GmPP2C126	D	4	3	43657.42	7.28	397	150	80	38	129
GmPP2C127	H	8	7	46513.07	7.56	431	130	105	47	149
GmPP2C128	D	4	3	41357.11	6.93	373	110	107	36	120
GmPP2C129	E	5	4	41147.49	6.14	367	112	96	49	110
GmPP2C130	H	7	6	46223.91	6.91	428	115	108	31	174
GmPP2C131	F	7	6	26675.13	4.7	243	96	54	25	68
GmPP2C132	D	4	3	42720.79	6.87	388	166	79	41	102
GmPP2C133	E	2	1	39896.1	7.16	365	89	89	40	147
GmPP2C134	F	5	4	30909.13	6.56	282	105	63	29	85
