# Supplementary material for: Pervasive duplication, biased molecular evolution and comprehensive functional analysis of the PP2C family in Glycine max
Source: BMC Genomics. 2020 Jul 6;21:465. doi: 10.1186/s12864-020-06877-4 (PMC7339511; doi:10.1186/s12864-020-06877-4)
Supplement: Supplementary file 17 [file 12864_2020_6877_MOESM15_ESM.docx]

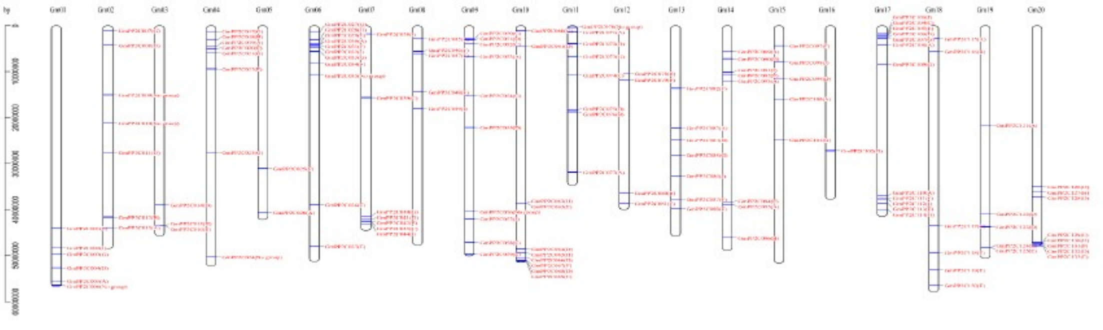

Additional file 15. Chromosomal locations of GmPP2Cs on all 20 chromosomes. The scale used is base pair (bp). Parentheses after the gene names show the PP2C subfamily.
