# Supplementary material for: Pervasive duplication, biased molecular evolution and comprehensive functional analysis of the PP2C family in Glycine max
Source: BMC Genomics. 2020 Jul 6;21:465. doi: 10.1186/s12864-020-06877-4 (PMC7339511; doi:10.1186/s12864-020-06877-4)
Supplement: Supplementary file 18 [file 12864_2020_6877_MOESM16_ESM.docx]

Additional file 16. Genomic locations of the *GmPP2C* genes.

Name	Subfamily	Chromosome	Start (bp)	End (bp)
GmPP2C001	E	Chr01	43941431	43943767
GmPP2C002	J	Chr01	48181351	48189905
GmPP2C003	G	Chr01	49612386	49615552
GmPP2C004	D	Chr01	52591670	52595306
GmPP2C005	A	Chr01	55384453	55386369
GmPP2C006	No group	Chr01	56369198	56375265
GmPP2C007	G	Chr02	893427	896145
GmPP2C008	D	Chr02	4136000	4141195
GmPP2C009	No group	Chr02	14908610	14922263
GmPP2C010	No group	Chr02	21052064	21076949
GmPP2C011	D	Chr02	27467209	27471468
GmPP2C012	B	Chr02	41552402	41554889
GmPP2C013	A	Chr02	43779470	43781548
GmPP2C014	D	Chr03	38923777	38927139
GmPP2C015	H	Chr03	43401396	43405307
GmPP2C016	E	Chr03	43430628	43434432
GmPP2C017	G	Chr04	1222604	1225058
GmPP2C018	H	Chr04	3020991	3026045
GmPP2C019	A	Chr04	4360436	4366002
GmPP2C020	F	Chr04	4841142	4845969
GmPP2C021	G	Chr04	5836511	5840001
GmPP2C022	F	Chr04	9326168	9328990
GmPP2C023	G	Chr04	27489012	27489497
GmPP2C024	No group	Chr04	50258694	50262696
GmPP2C025	F	Chr05	30957155	30965495
GmPP2C026	A	Chr05	40527741	40537026
GmPP2C027	G	Chr06	1202587	1205865

GmPP2C028	H	Chr06	2949704	2954653
GmPP2C029	E	Chr06	3863762	3865651
GmPP2C030	A	Chr06	4085259	4091133
GmPP2C031	F	Chr06	4525998	4530480
GmPP2C032	I	Chr06	4594593	4600931
GmPP2C033	G	Chr06	5490395	5493713
GmPP2C034	F	Chr06	8193146	8196805
GmPP2C035	No group	Chr06	10753446	10757827
GmPP2C036	F	Chr06	38907277	38911596
GmPP2C037	E	Chr06	47906561	47909375
GmPP2C038	I	Chr07	1684549	1692320
GmPP2C039	C	Chr07	15563830	15567635
GmPP2C040	G	Chr07	41377147	41379829
GmPP2C041	D	Chr07	41997939	42002698
GmPP2C042	E	Chr07	42413071	42416331
GmPP2C043	C	Chr07	42643847	42645758
GmPP2C044	J	Chr07	43091095	43094864
GmPP2C045	A	Chr08	2698773	2705055
GmPP2C046	F	Chr08	5496732	5505069
GmPP2C047	F	Chr08	6152131	6155753
GmPP2C048	F	Chr08	14320298	14325223
GmPP2C049	I	Chr08	17880513	17887186
GmPP2C050	G	Chr09	2609839	2612385
GmPP2C051	D	Chr09	2921110	2926995
GmPP2C052	C	Chr09	3862717	3864565
GmPP2C053	A	Chr09	6530984	6535359
GmPP2C054	G	Chr09	15045850	15050522
GmPP2C055	D	Chr09	22038949	22043788
GmPP2C056	No group	Chr09	40391988	40396974

GmPP2C057	J	Chr09	41851899	41861697
GmPP2C058	E	Chr09	47064929	47069494
GmPP2C059	E	Chr09	49578261	49582235
GmPP2C060	G	Chr10	915906	918730
GmPP2C061	D	Chr10	4254833	4258007
GmPP2C062	H	Chr10	38511278	38514578
GmPP2C063	E	Chr10	38541140	38544102
GmPP2C064	D	Chr10	48531124	48534411
GmPP2C065	H	Chr10	49382343	49387295
GmPP2C066	D	Chr10	50358644	50363494
GmPP2C067	F	Chr10	50970944	50977093
GmPP2C068	D	Chr10	51164361	51168273
GmPP2C069	E	Chr10	51453291	51454190
GmPP2C070	No group	Chr11	299685	304643
GmPP2C071	A	Chr11	1246178	1247949
GmPP2C072	D	Chr11	3801015	3805086
GmPP2C073	G	Chr11	6526033	6529595
GmPP2C074	C	Chr11	10627630	10631082
GmPP2C075	B	Chr11	18166537	18168852
GmPP2C076	B	Chr11	18636508	18638912
GmPP2C077	A	Chr11	31769175	31771265
GmPP2C078	E	Chr12	10308026	10314092
GmPP2C079	F	Chr12	11703333	11706380
GmPP2C080	E	Chr12	36289704	36294129
GmPP2C081	F	Chr12	38562918	38565937
GmPP2C082	F	Chr13	13516047	13530998
GmPP2C083	A	Chr13	22115158	22119612
GmPP2C084	D	Chr13	24770681	24773826
GmPP2C085	G	Chr13	28011615	28015224

GmPP2C086	J	Chr13	32552265	32556704
GmPP2C087	F	Chr13	37649393	37653534
GmPP2C088	E	Chr13	39758271	39762979
GmPP2C089	A	Chr14	5469253	5471523
GmPP2C090	H	Chr14	7100065	7105778
GmPP2C091	I	Chr14	10034501	10040535
GmPP2C092	F	Chr14	10755592	10760847
GmPP2C093	A	Chr14	11901726	11906954
GmPP2C094	F	Chr14	38181392	38192562
GmPP2C095	A	Chr14	38860097	38863074
GmPP2C096	B	Chr14	46049236	46051706
GmPP2C097	F	Chr15	4224743	4229800
GmPP2C098	J	Chr15	7855960	7860113
GmPP2C099	D	Chr15	11384148	11390333
GmPP2C100	A	Chr15	15791210	15796087
GmPP2C101	G	Chr15	24734338	24738231
GmPP2C102	D	Chr16	27104326	27110822
GmPP2C103	J	Chr17	1513719	1517368
GmPP2C104	C	Chr17	1922609	1925789
GmPP2C105	E	Chr17	2157196	2159909
GmPP2C106	D	Chr17	2518522	2522983
GmPP2C107	G	Chr17	2806050	2808803
GmPP2C108	A	Chr17	3977763	3981813
GmPP2C109	G	Chr17	8293050	8296327
GmPP2C110	A	Chr17	36986191	36990855
GmPP2C111	F	Chr17	37364357	37369545
GmPP2C112	I	Chr17	37680923	37686740
GmPP2C113	E	Chr17	38566124	38570491
GmPP2C114	H	Chr17	39903187	39908974

GmPP2C115	A	Chr18	2727985	2730047
GmPP2C116	B	Chr18	5498935	5501512
GmPP2C117	C	Chr18	43424554	43427474
GmPP2C118	E	Chr18	49286980	49291000
GmPP2C119	E	Chr18	53105841	53110267
GmPP2C120	E	Chr18	56411520	56416642
GmPP2C121	A	Chr19	21550938	21554520
GmPP2C122	D	Chr19	40846631	40850759
GmPP2C123	D	Chr19	43642041	43645437
GmPP2C124	H	Chr19	48103324	48106756
GmPP2C125	E	Chr19	48140101	48144083
GmPP2C126	D	Chr20	34935548	34940516
GmPP2C127	H	Chr20	36180104	36185840
GmPP2C128	D	Chr20	37212906	37215895
GmPP2C129	E	Chr20	46996209	47000167
GmPP2C130	H	Chr20	47035066	47038474
GmPP2C131	F	Chr20	47224329	47228932
GmPP2C132	D	Chr20	47442173	47446634
GmPP2C133	E	Chr20	47792825	47794559
GmPP2C134	F	scaffold_23	688255	692458
