# Supplementary material for: Pervasive duplication, biased molecular evolution and comprehensive functional analysis of the PP2C family in Glycine max
Source: BMC Genomics. 2020 Jul 6;21:465. doi: 10.1186/s12864-020-06877-4 (PMC7339511; doi:10.1186/s12864-020-06877-4)
Supplement: Supplementary file 19 [file 12864_2020_6877_MOESM17_ESM.docx]

Additional file 17. Ka/Ks analysis for the duplicated PP2Cs in soybean.

Duplicated gene 1	Duplicated gene 2	Subfamily	Ka	Ks	Ka/Ks	Purifying selection
GmPP2C001	GmPP2C059	E	0.203805	0.654997	0.311154	Yes
GmPP2C001	GmPP2C118	E	0.203184	0.57676	0.352285	Yes
GmPP2C002	GmPP2C057	J	0.022405	0.103967	0.215496	Yes
GmPP2C003	GmPP2C027	G	0.28179	2.521297	0.111764	Yes
GmPP2C003	GmPP2C073	G	0.021195	0.134061	0.158097	Yes
GmPP2C004	GmPP2C072	D	0.024378	0.113612	0.214571	Yes
GmPP2C004	GmPP2C128	D	0.215075	2.43196	0.088437	Yes
GmPP2C005	GmPP2C071	A	0.100898	0.199399	0.506012	Yes
GmPP2C006	GmPP2C070	No group	0.028266	0.09528	0.296659	Yes
GmPP2C007	GmPP2C060	G	0.017855	0.127638	0.139885	Yes
GmPP2C007	GmPP2C107	G	0.293442	2.151032	0.136419	Yes
GmPP2C008	GmPP2C102	D	0.021888	0.087763	0.249394	Yes
GmPP2C011	GmPP2C055	D	0.026816	0.132851	0.201848	Yes
GmPP2C011	GmPP2C122	D	0.140754	0.718706	0.195843	Yes
GmPP2C012	GmPP2C075	B	0.058189	0.559263	0.104046	Yes
GmPP2C012	GmPP2C076	B	0.140875	0.739176	0.190584	Yes
GmPP2C012	GmPP2C096	B	0.01864	0.117204	0.159038	Yes
GmPP2C012	GmPP2C116	B	0.143358	0.702207	0.204154	Yes
GmPP2C013	GmPP2C077	A	0.212626	0.892231	0.238308	Yes
GmPP2C013	GmPP2C089	A	0.058488	0.240813	0.242877	Yes
GmPP2C013	GmPP2C115	A	0.199539	0.876057	0.22777	Yes
GmPP2C014	GmPP2C061	D	0.133153	0.568146	0.234365	Yes
GmPP2C014	GmPP2C084	D	0.128987	0.564731	0.228405	Yes
GmPP2C014	GmPP2C123	D	0.01472	0.113053	0.130204	Yes
GmPP2C015	GmPP2C062	H	0.069678	0.432823	0.160986	Yes
GmPP2C015	GmPP2C124	H	0.014673	0.103078	0.142348	Yes
GmPP2C015	GmPP2C130	H	0.067376	0.378026	0.17823	Yes

GmPP2C016	GmPP2C063	E	0.104043	0.490943	0.211925	Yes
GmPP2C016	GmPP2C105	E	0.248471	2.482159	0.100103	Yes
GmPP2C016	GmPP2C125	E	0.014073	0.081421	0.172836	Yes
GmPP2C016	GmPP2C129	E	0.102513	0.497471	0.206068	Yes
GmPP2C017	GmPP2C027	G	0.03889	0.129378	0.300591	Yes
GmPP2C017	GmPP2C073	G	0.346917	1.96362	0.176672	Yes
GmPP2C018	GmPP2C028	H	0.021185	0.095866	0.220982	Yes
GmPP2C018	GmPP2C090	H	0.08303	0.50521	0.164347	Yes
GmPP2C018	GmPP2C114	H	0.086157	0.515967	0.166981	Yes
GmPP2C019	GmPP2C030	A	0.028138	0.108004	0.260526	Yes
GmPP2C020	GmPP2C031	F	0.024851	0.104665	0.237437	Yes
GmPP2C020	GmPP2C092	F	0.043444	0.385865	0.112589	Yes
GmPP2C020	GmPP2C111	F	0.043472	0.40108	0.108387	Yes
GmPP2C021	GmPP2C033	G	0.016533	0.142773	0.115798	Yes
GmPP2C022	GmPP2C034	F	0.048574	0.17808	0.272763	Yes
GmPP2C024	GmPP2C035	No group	0.026169	0.100797	0.259619	Yes
GmPP2C025	GmPP2C046	F	0.009302	0.074662	0.124594	Yes
GmPP2C025	GmPP2C048	F	0.088518	0.409673	0.216071	Yes
GmPP2C025	GmPP2C097	F	0.10113	0.399587	0.253087	Yes
GmPP2C026	GmPP2C045	A	0.027829	0.097476	0.285498	Yes
GmPP2C027	GmPP2C073	G	0.281656	2.057123	0.136918	Yes
GmPP2C028	GmPP2C090	H	0.077235	0.527424	0.146438	Yes
GmPP2C028	GmPP2C114	H	0.080912	0.528569	0.153077	Yes
GmPP2C029	GmPP2C113	E	0.213829	0.478894	0.446507	Yes
GmPP2C031	GmPP2C092	F	0.050575	0.343012	0.147444	Yes
GmPP2C031	GmPP2C111	F	0.050608	0.381053	0.13281	Yes
GmPP2C032	GmPP2C049	I	0.119731	1.23141	0.097231	Yes
GmPP2C032	GmPP2C091	I	0.095607	0.443532	0.215557	Yes
GmPP2C032	GmPP2C112	I	0.097888	0.436837	0.224084	Yes

GmPP2C036	GmPP2C081	F	0.097493	0.552759	0.176375	Yes
GmPP2C037	GmPP2C078	E	0.013807	0.108572	0.127171	Yes
GmPP2C037	GmPP2C080	E	0.074558	0.613962	0.121437	Yes
GmPP2C037	GmPP2C088	E	0.081571	0.573632	0.142201	Yes
GmPP2C038	GmPP2C049	I	0.015214	0.051559	0.29508	Yes
GmPP2C039	GmPP2C117	C	0.038967	0.152491	0.255533	Yes
GmPP2C040	GmPP2C107	G	0.017478	0.154757	0.11294	Yes
GmPP2C041	GmPP2C051	D	0.110745	0.720454	0.153716	Yes
GmPP2C041	GmPP2C099	D	0.109983	0.651631	0.168782	Yes
GmPP2C041	GmPP2C106	D	0.012356	0.116006	0.106509	Yes
GmPP2C042	GmPP2C105	E	0.014731	0.089074	0.165379	Yes
GmPP2C042	GmPP2C125	E	0.247216	2.065587	0.119683	Yes
GmPP2C043	GmPP2C052	C	0.110647	0.468931	0.235956	Yes
GmPP2C043	GmPP2C104	C	0.029313	0.083685	0.350284	Yes
GmPP2C044	GmPP2C086	J	0.138479	0.501228	0.27628	Yes
GmPP2C044	GmPP2C098	J	0.13821	0.505641	0.273336	Yes
GmPP2C044	GmPP2C103	J	0.04797	0.109028	0.439974	Yes
GmPP2C046	GmPP2C048	F	0.087721	0.403833	0.217221	Yes
GmPP2C046	GmPP2C097	F	0.088264	0.374896	0.235437	Yes
GmPP2C048	GmPP2C097	F	0.018852	0.066189	0.284815	Yes
GmPP2C050	GmPP2C107	G	0.098366	0.729923	0.134762	Yes
GmPP2C051	GmPP2C099	D	0.014133	0.098943	0.142844	Yes
GmPP2C051	GmPP2C106	D	0.110954	0.684736	0.162039	Yes
GmPP2C052	GmPP2C104	C	0.107998	0.514069	0.210084	Yes
GmPP2C053	GmPP2C083	A	0.20323	0.561359	0.362033	Yes
GmPP2C053	GmPP2C100	A	0.045361	0.120661	0.375941	Yes
GmPP2C053	GmPP2C108	A	0.202139	0.514184	0.393125	Yes
GmPP2C054	GmPP2C085	G	0.133281	0.708275	0.188177	Yes
GmPP2C054	GmPP2C101	G	0.034702	0.101707	0.341192	Yes

GmPP2C054	GmPP2C109	G	0.140337	0.663913	0.211379	Yes
GmPP2C055	GmPP2C122	D	0.124876	0.752835	0.165874	Yes
GmPP2C058	GmPP2C119	E	0.023851	0.08435	0.282762	Yes
GmPP2C059	GmPP2C118	E	0.020668	0.095162	0.217184	Yes
GmPP2C061	GmPP2C084	D	0.008258	0.093872	0.087971	Yes
GmPP2C061	GmPP2C123	D	0.127477	0.583241	0.218567	Yes
GmPP2C062	GmPP2C124	H	0.06754	0.426201	0.158469	Yes
GmPP2C062	GmPP2C130	H	0.018709	0.118645	0.157687	Yes
GmPP2C063	GmPP2C125	E	0.115512	0.524385	0.22028	Yes
GmPP2C063	GmPP2C129	E	0.017763	0.108966	0.163013	Yes
GmPP2C064	GmPP2C128	D	0.017945	0.145837	0.12305	Yes
GmPP2C065	GmPP2C127	H	0.006162	0.098113	0.062808	Yes
GmPP2C066	GmPP2C126	D	0.009439	0.067936	0.138944	Yes
GmPP2C067	GmPP2C131	F	0.057963	0.155086	0.373746	Yes
GmPP2C068	GmPP2C132	D	0.021521	0.082625	0.260466	Yes
GmPP2C069	GmPP2C133	E	0.241597	0.300936	0.802816	Yes
GmPP2C075	GmPP2C076	B	0	0.012726	0	Yes
GmPP2C075	GmPP2C096	B	0.145607	0.692134	0.210374	Yes
GmPP2C075	GmPP2C116	B	0.032675	0.162798	0.200711	Yes
GmPP2C076	GmPP2C096	B	0.144832	0.712286	0.203333	Yes
GmPP2C076	GmPP2C116	B	0.031227	0.167358	0.186587	Yes
GmPP2C077	GmPP2C089	A	0.190987	1.051405	0.18165	Yes
GmPP2C077	GmPP2C115	A	0.039801	0.208996	0.190439	Yes
GmPP2C078	GmPP2C080	E	0.07377	0.624925	0.118047	Yes
GmPP2C078	GmPP2C088	E	0.081121	0.541909	0.149695	Yes
GmPP2C080	GmPP2C088	E	0.030127	0.11948	0.252151	Yes
GmPP2C081	GmPP2C087	F	0.018741	0.088517	0.211719	Yes
GmPP2C083	GmPP2C100	A	0.213388	0.558823	0.381853	Yes
GmPP2C083	GmPP2C108	A	0.029761	0.129599	0.229642	Yes

GmPP2C084	GmPP2C123	D	0.124854	0.588219	0.212257	Yes
GmPP2C085	GmPP2C101	G	0.13194	0.677284	0.194808	Yes
GmPP2C085	GmPP2C109	G	0.023328	0.148232	0.157376	Yes
GmPP2C086	GmPP2C098	J	0.011272	0.126282	0.089258	Yes
GmPP2C086	GmPP2C103	J	0.171761	0.536971	0.319869	Yes
GmPP2C089	GmPP2C115	A	0.189122	0.999327	0.189249	Yes
GmPP2C090	GmPP2C114	H	0.007699	0.118832	0.064789	Yes
GmPP2C091	GmPP2C112	I	0.019154	0.120937	0.15838	Yes
GmPP2C092	GmPP2C111	F	0.014331	0.098172	0.14598	Yes
GmPP2C093	GmPP2C110	A	0.03069	0.129564	0.23687	Yes
GmPP2C096	GmPP2C116	B	0.14826	0.7207	0.205717	Yes
GmPP2C098	GmPP2C103	J	0.173766	0.558063	0.311374	Yes
GmPP2C099	GmPP2C106	D	0.102643	0.634514	0.161767	Yes
GmPP2C100	GmPP2C108	A	0.204429	0.504441	0.405259	Yes
GmPP2C101	GmPP2C109	G	0.138983	0.663204	0.209563	Yes
GmPP2C124	GmPP2C130	H	0.065235	0.38887	0.167756	Yes
GmPP2C125	GmPP2C129	E	0.103826	0.530547	0.195697	Yes
