# Supplementary material for: Pervasive duplication, biased molecular evolution and comprehensive functional analysis of the PP2C family in Glycine max
Source: BMC Genomics. 2020 Jul 6;21:465. doi: 10.1186/s12864-020-06877-4 (PMC7339511; doi:10.1186/s12864-020-06877-4)
Supplement: Supplementary file 20 [file 12864_2020_6877_MOESM18_ESM.docx]

Additional file 18. Ka/Ks analysis for the duplicated PP2Cs in grape.

Duplicated gene 1	Duplicated gene 2	Subfamily	Ka	Ks	Ka/Ks	Purifying selection
VvPP2C18	VvPP2C23	A	0.243509594	1.195785041	0.20363994	Yes
VvPP2C55	VvPP2C23	A	0.234769274	1.059539185	0.221576773	Yes
VvPP2C19	VvPP2C24	A	0.323973704	1.271419418	0.254812613	Yes
VvPP2C42	VvPP2C31	A	0.353335787	NaN	NaN	NaN
VvPP2C22	VvPP2C37	A	0.151893198	1.67211408	0.090839016	Yes
VvPP2C18	VvPP2C55	A	0.212886109	1.26860633	0.16781101	Yes
VvPP2C49	VvPP2C36	B	0.158113496	1.184883183	0.133442265	Yes
VvPP2C10	VvPP2C01	D	0.165893874	1.151385856	0.14408191	Yes
VvPP2C60	VvPP2C01	D	0.138460583	1.794099566	0.077175529	Yes
VvPP2C48	VvPP2C39	D	0.169141041	1.736761672	0.097388746	Yes
VvPP2C10	VvPP2C60	D	0.161418514	1.469449455	0.109849654	Yes
VvPP2C33	VvPP2C16	F	0.154288177	1.290901741	0.119519691	Yes
VvPP2C15	VvPP2C34	G	0.135018314	1.260478135	0.107116745	Yes
