# Supplementary material for: Pervasive duplication, biased molecular evolution and comprehensive functional analysis of the PP2C family in Glycine max
Source: BMC Genomics. 2020 Jul 6;21:465. doi: 10.1186/s12864-020-06877-4 (PMC7339511; doi:10.1186/s12864-020-06877-4)
Supplement: Supplementary file 21 [file 12864_2020_6877_MOESM19_ESM.docx]

Additional file 19. Ka/Ks analysis for GmPP2Cs and its corresponding orthologous genes in grape.

GmPP2C name	Name of its corresponding orthologous gene in grape	subfamily	Ka	Ks	Ka_Ks	Purifying selection
GmPP2C004	VvPP2C01	D	0.167674	1.622306	0.103355	Yes
GmPP2C072	VvPP2C01	D	0.155977	1.613071	0.096696	Yes
GmPP2C009	VvPP2C04	No group	0.391902	1.860263	0.21067	Yes
GmPP2C056	VvPP2C08	No group	0.204633	1.000299	0.204572	Yes
GmPP2C022	VvPP2C09	F	0.121685	1.522136	0.079944	Yes
GmPP2C034	VvPP2C09	F	0.103712	1.250493	0.082937	Yes
GmPP2C018	VvPP2C11	H	0.118669	1.082941	0.10958	Yes
GmPP2C028	VvPP2C11	H	0.106658	1.077639	0.098974	Yes
GmPP2C090	VvPP2C11	H	0.096521	1.061002	0.090971	Yes
GmPP2C114	VvPP2C11	H	0.097252	1.047613	0.092832	Yes
GmPP2C065	VvPP2C13	H	0.06912	0.834864	0.082792	Yes
GmPP2C127	VvPP2C13	H	0.067413	0.821156	0.082095	Yes
GmPP2C085	VvPP2C14	G	0.156456	1.435962	0.108955	Yes
GmPP2C101	VvPP2C14	G	0.1729	1.219162	0.141819	Yes
GmPP2C109	VvPP2C14	G	0.149821	1.512197	0.099075	Yes
GmPP2C017	VvPP2C15	G	0.173464	1.203024	0.14419	Yes
GmPP2C027	VvPP2C15	G	0.138233	1.359346	0.101691	Yes
GmPP2C081	VvPP2C16	F	0.145289	0.866339	0.167705	Yes
GmPP2C087	VvPP2C16	F	0.143636	0.879747	0.163269	Yes
GmPP2C026	VvPP2C19	A	0.144012	0.956829	0.15051	Yes
GmPP2C045	VvPP2C19	A	0.142565	0.906817	0.157214	Yes
GmPP2C013	VvPP2C22	A	0.203603	3.55582	0.057259	Yes
GmPP2C115	VvPP2C22	A	0.18383	2.123592	0.086566	Yes
GmPP2C077	VvPP2C22	A	0.171843	2.003856	0.085756	Yes
GmPP2C040	VvPP2C26	G	0.23707	1.69915	0.139523	Yes
GmPP2C050	VvPP2C26	G	0.240852	1.682489	0.143152	Yes

GmPP2C107	VvPP2C26	G	0.236841	1.461102	0.162098	Yes
GmPP2C043	VvPP2C27	C	0.13229	0.794565	0.166494	Yes
GmPP2C052	VvPP2C27	C	0.148421	0.867327	0.171125	Yes
GmPP2C104	VvPP2C27	C	0.145958	0.847118	0.172299	Yes
GmPP2C006	VvPP2C28	No group	0.216578	0.984404	0.220009	Yes
GmPP2C070	VvPP2C28	No group	0.213552	0.99064	0.215569	Yes
GmPP2C058	VvPP2C30	E	0.177365	1.453964	0.121988	Yes
GmPP2C119	VvPP2C30	E	0.164904	1.323255	0.12462	Yes
GmPP2C005	VvPP2C31	A	0.397036	2.845096	0.139551	Yes
GmPP2C071	VvPP2C31	A	0.392286	2.828907	0.138671	Yes
GmPP2C079	VvPP2C33	F	0.162852	1.774254	0.091786	Yes
GmPP2C003	VvPP2C34	G	0.155544	1.262692	0.123184	Yes
GmPP2C073	VvPP2C34	G	0.157729	1.30056	0.121277	Yes
GmPP2C008	VvPP2C35	D	0.14437	1.437368	0.100441	Yes
GmPP2C066	VvPP2C35	D	0.087631	1.014207	0.086404	Yes
GmPP2C126	VvPP2C35	D	0.096947	1.037522	0.093441	Yes
GmPP2C012	VvPP2C36	B	0.142026	1.028252	0.138124	Yes
GmPP2C075	VvPP2C36	B	0.160394	1.869318	0.085804	Yes
GmPP2C096	VvPP2C36	B	0.146663	1.027635	0.142719	Yes
GmPP2C116	VvPP2C36	B	0.153838	1.571147	0.097915	Yes
GmPP2C039	VvPP2C38	C	0.446442	1.948054	0.229173	Yes
GmPP2C074	VvPP2C38	C	0.111098	1.709052	0.065006	Yes
GmPP2C014	VvPP2C39	D	0.171214	1.495427	0.114491	Yes
GmPP2C061	VvPP2C39	D	0.190959	1.127694	0.169336	Yes
GmPP2C084	VvPP2C39	D	0.18893	1.161776	0.162621	Yes
GmPP2C123	VvPP2C39	D	0.176245	1.290184	0.136605	Yes
GmPP2C007	VvPP2C41	G	0.1693	1.326105	0.127667	Yes
GmPP2C060	VvPP2C41	G	0.159238	1.42784	0.111524	Yes
GmPP2C059	VvPP2C44	E	0.336387	1.395265	0.241092	Yes

GmPP2C118	VvPP2C44	E	0.325592	1.412009	0.230588	Yes
GmPP2C037	VvPP2C45	E	0.100666	0.997593	0.100909	Yes
GmPP2C078	VvPP2C45	E	0.097251	1.087705	0.089409	Yes
GmPP2C080	VvPP2C45	E	0.103598	1.259076	0.082281	Yes
GmPP2C088	VvPP2C45	E	0.124793	1.234318	0.101103	Yes
GmPP2C016	VvPP2C46	E	0.120206	1.555976	0.077254	Yes
GmPP2C063	VvPP2C46	E	0.131181	1.344582	0.097563	Yes
GmPP2C125	VvPP2C46	E	0.120013	1.502811	0.079859	Yes
GmPP2C015	VvPP2C47	H	0.073428	0.991317	0.074071	Yes
GmPP2C062	VvPP2C47	H	0.093131	0.995567	0.093546	Yes
GmPP2C124	VvPP2C47	H	0.06541	0.972316	0.067273	Yes
GmPP2C130	VvPP2C47	H	0.08439	1.007374	0.083772	Yes
GmPP2C068	VvPP2C48	D	0.177095	1.313825	0.134794	Yes
GmPP2C132	VvPP2C48	D	0.173375	1.287569	0.134653	Yes
GmPP2C011	VvPP2C50	D	0.124641	1.070987	0.116379	Yes
GmPP2C055	VvPP2C50	D	0.117022	1.0382	0.112716	Yes
GmPP2C122	VvPP2C50	D	0.161132	1.225646	0.131467	Yes
GmPP2C041	VvPP2C52	D	0.098293	0.99976	0.098317	Yes
GmPP2C051	VvPP2C52	D	0.123953	1.340952	0.092437	Yes
GmPP2C099	VvPP2C52	D	0.123967	1.194732	0.103761	Yes
GmPP2C106	VvPP2C52	D	0.104138	0.94325	0.110404	Yes
GmPP2C042	VvPP2C53	E	0.220482	1.134467	0.194348	Yes
GmPP2C105	VvPP2C53	E	0.229421	1.110271	0.206635	Yes
GmPP2C029	VvPP2C54	E	0.208059	0.971937	0.214066	Yes
GmPP2C113	VvPP2C54	E	0.233454	0.976122	0.239164	Yes
GmPP2C019	VvPP2C55	A	0.185056	1.056444	0.175169	Yes
GmPP2C083	VvPP2C55	A	0.369938	2.364998	0.156422	Yes
GmPP2C021	VvPP2C56	G	0.134294	1.273096	0.105486	Yes
GmPP2C033	VvPP2C56	G	0.136682	1.322528	0.103349	Yes

GmPP2C032	VvPP2C57	I	0.074376	0.878037	0.084708	Yes
GmPP2C091	VvPP2C57	I	0.089207	0.912987	0.097709	Yes
GmPP2C112	VvPP2C57	I	0.097215	0.841764	0.115489	Yes
GmPP2C020	VvPP2C58	F	0.050016	0.765526	0.065336	Yes
GmPP2C031	VvPP2C58	F	0.05808	0.819167	0.070901	Yes
GmPP2C092	VvPP2C58	F	0.043144	0.914476	0.047179	Yes
GmPP2C111	VvPP2C58	F	0.043172	0.805608	0.053589	Yes
GmPP2C064	VvPP2C60	D	0.124795	1.13651	0.109805	Yes
GmPP2C128	VvPP2C60	D	0.122836	1.187267	0.103461	Yes
GmPP2C024	VvPP2C61	No group	0.211668	1.022423	0.207026	Yes
GmPP2C035	VvPP2C61	No group	0.213925	0.99149	0.215761	Yes
GmPP2C046	VvPP2C62	F	0.10284	0.947051	0.10859	Yes
