# Supplementary material for: Pervasive duplication, biased molecular evolution and comprehensive functional analysis of the PP2C family in Glycine max
Source: BMC Genomics. 2020 Jul 6;21:465. doi: 10.1186/s12864-020-06877-4 (PMC7339511; doi:10.1186/s12864-020-06877-4)
Supplement: Supplementary file 22 [file 12864_2020_6877_MOESM1_ESM.docx]

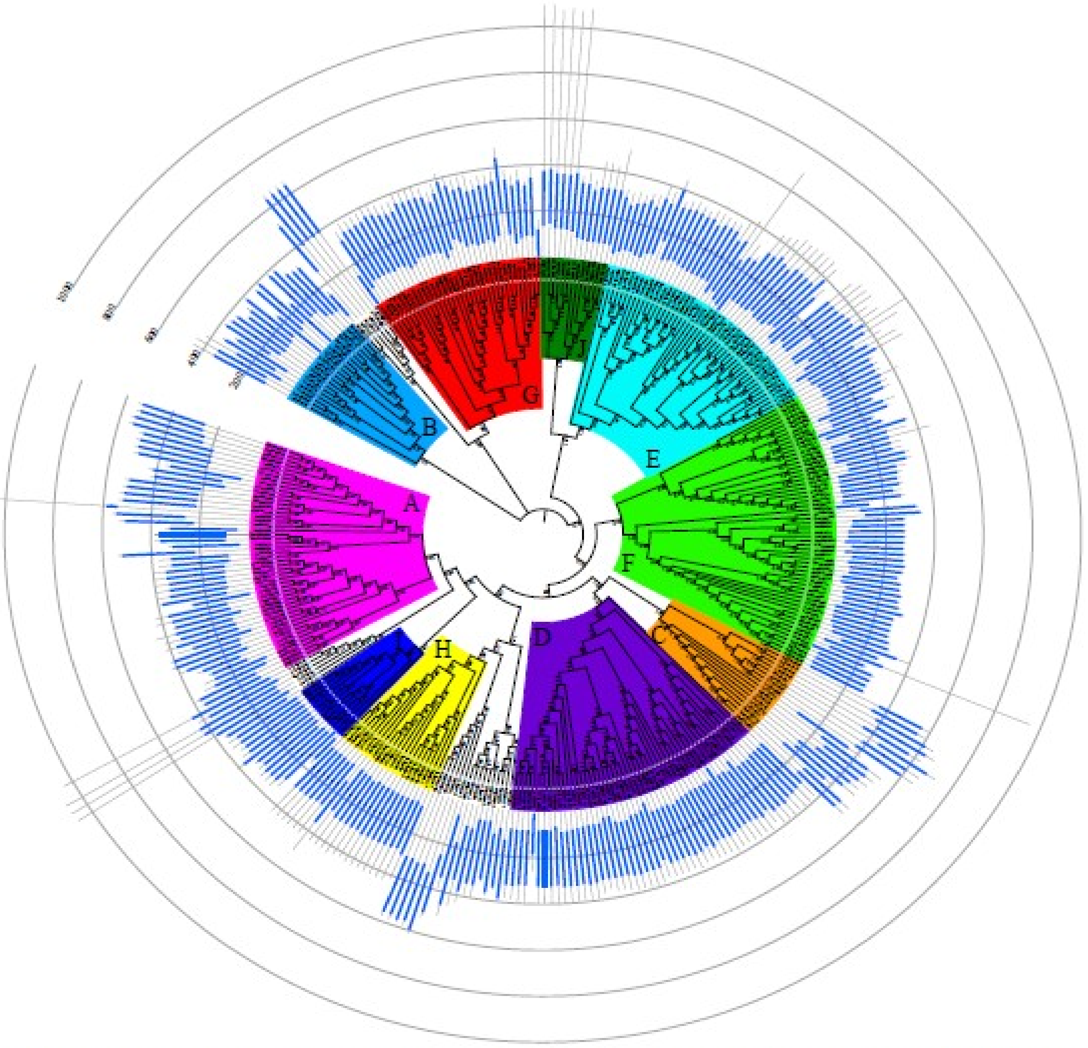

Additional file 1. Phylogenetic and sequence analysis of the PP2C members in soybean, Arabidopsis, grape, *A.trichopoda* and *N.colorata*. The inner circle is the phylogenetic tree using the FastTree method The numbers in the clades are the FastTree bootstrap values. The PP2C subfamilies are labeled using different letters and are indicated using different colors. The outer circle is the sequence analysis of PP2Cs. The conserved PP2C domain was represented by the blue box. The location of each PP2C protein can be estimated using the scale at the end of the circle.
