# Supplementary material for: Pervasive duplication, biased molecular evolution and comprehensive functional analysis of the PP2C family in Glycine max
Source: BMC Genomics. 2020 Jul 6;21:465. doi: 10.1186/s12864-020-06877-4 (PMC7339511; doi:10.1186/s12864-020-06877-4)
Supplement: Supplementary file 23 [file 12864_2020_6877_MOESM20_ESM.docx]

Additional file 20. Number of VvPP2C orthologous genes in soybean.

Name	Number orthologous in GmPP2C	Subfamily
VvPP2C04	1	No group
VvPP2C08	1	No group
VvPP2C33	1	F
VvPP2C62	1	F
VvPP2C01	2	D
VvPP2C09	2	F
VvPP2C13	2	H
VvPP2C15	2	G
VvPP2C16	2	F
VvPP2C19	2	A
VvPP2C28	2	No group
VvPP2C30	2	E
VvPP2C31	2	A
VvPP2C34	2	G
VvPP2C38	2	C
VvPP2C41	2	G
VvPP2C44	2	E
VvPP2C48	2	D
VvPP2C53	2	E
VvPP2C54	2	E
VvPP2C55	2	A
VvPP2C56	2	G
VvPP2C60	2	D
VvPP2C61	2	No group
VvPP2C14	3	G
VvPP2C22	3	A
VvPP2C26	3	G

VvPP2C27	3	C
VvPP2C35	3	D
VvPP2C46	3	E
VvPP2C50	3	D
VvPP2C57	3	I
VvPP2C11	4	H
VvPP2C36	4	B
VvPP2C39	4	D
VvPP2C45	4	E
VvPP2C47	4	H
VvPP2C52	4	D
VvPP2C58	4	F
