# Supplementary material for: Pervasive duplication, biased molecular evolution and comprehensive functional analysis of the PP2C family in Glycine max
Source: BMC Genomics. 2020 Jul 6;21:465. doi: 10.1186/s12864-020-06877-4 (PMC7339511; doi:10.1186/s12864-020-06877-4)
Supplement: Supplementary file 24 [file 12864_2020_6877_MOESM2_ESM.docx]

Additional file 2. GmPP2C proteins identified in *G. max*

Name	Locus name	Subfamily	E-value	Score
GmPP2C001	Glyma.01G127600	E	5.10E-40	140
GmPP2C002	Glyma.01G146600	J	5.30E-35	123.6
GmPP2C003	Glyma.01G158000	G	6.10E-68	231.5
GmPP2C004	Glyma.01G191300	D	7.10E-37	129.7
GmPP2C005	Glyma.01G225100	A	5.00E-63	215.4
GmPP2C006	Glyma.01G239700	No group	1.40E-45	158.3
GmPP2C007	Glyma.02G009100	G	3.50E-66	225.8
GmPP2C008	Glyma.02G044700	D	1.00E-37	132.5
GmPP2C009	Glyma.02G144500	No group	5.00E-43	149.9
GmPP2C010	Glyma.02G162300	No group	1.10E-61	211
GmPP2C011	Glyma.02G172900	D	2.00E-39	138.1
GmPP2C012	Glyma.02G228200	B	3.10E-61	209.6
GmPP2C013	Glyma.02G250200	A	4.20E-68	232
GmPP2C014	Glyma.03G176100	D	5.40E-37	130.1
GmPP2C015	Glyma.03G233300	H	3.30E-31	111.2
GmPP2C016	Glyma.03G233500	E	8.50E-39	136
GmPP2C017	Glyma.04G016100	G	7.00E-55	188.7
GmPP2C018	Glyma.04G038000	H	7.70E-29	103.4
GmPP2C019	Glyma.04G053800	A	4.50E-66	225.4
GmPP2C020	Glyma.04G059000	F	6.70E-67	228.1

GmPP2C021	Glyma.04G069900	G	1.70E-68	233.3
GmPP2C022	Glyma.04G101400	F	1.50E-57	197.4
GmPP2C023	Glyma.04G146200	G	4.00E-38	133.8
GmPP2C024	Glyma.04G233900	No group	4.80E-55	189.2
GmPP2C025	Glyma.05G116800	F	1.40E-53	184.4
GmPP2C026	Glyma.05G227100	A	5.80E-58	198.8
GmPP2C027	Glyma.06G016100	G	1.10E-72	247.1
GmPP2C028	Glyma.06G039000	H	1.20E-29	106
GmPP2C029	Glyma.06G050900	E	3.20E-40	140.7
GmPP2C030	Glyma.06G054000	A	5.20E-65	221.9
GmPP2C031	Glyma.06G059700	F	1.60E-68	233.4
GmPP2C032	Glyma.06G060800	I	4.00E-74	251.8
GmPP2C033	Glyma.06G071900	G	5.90E-69	234.8
GmPP2C034	Glyma.06G103100	F	1.40E-57	197.5
GmPP2C035	Glyma.06G130900	No group	6.70E-55	188.8
GmPP2C036	Glyma.06G238200	F	3.10E-51	176.7
GmPP2C037	Glyma.06G296200	E	5.30E-38	133.4
GmPP2C038	Glyma.07G021700	I	1.70E-75	256.2
GmPP2C039	Glyma.07G131800	C	2.90E-31	111.3
GmPP2C040	Glyma.07G233000	G	3.70E-64	219.1
GmPP2C041	Glyma.07G239000	D	6.60E-37	129.8
GmPP2C042	Glyma.07G244700	E	1.10E-41	145.5

GmPP2C043	Glyma.07G248000	C	1.90E-31	111.9
GmPP2C044	Glyma.07G253900	J	4.20E-40	140.3
GmPP2C045	Glyma.08G033800	A	3.30E-59	202.9
GmPP2C046	Glyma.08G071900	F	2.20E-53	183.8
GmPP2C047	Glyma.08G081400	F	1.50E-58	200.7
GmPP2C048	Glyma.08G178800	F	1.10E-54	188
GmPP2C049	Glyma.08G220100	I	1.20E-74	253.5
GmPP2C050	Glyma.09G031700	G	4.00E-65	222.3
GmPP2C051	Glyma.09G034800	D	1.10E-35	125.8
GmPP2C052	Glyma.09G045100	C	4.10E-34	120.6
GmPP2C053	Glyma.09G066500	A	8.80E-69	234.3
GmPP2C054	Glyma.09G096600	G	3.50E-69	235.6
GmPP2C055	Glyma.09G111300	D	7.70E-39	136.1
GmPP2C056	Glyma.09G179200	No group	4.50E-52	179.5
GmPP2C057	Glyma.09G194200	J	9.50E-35	122.7
GmPP2C058	Glyma.09G249700	E	1.40E-40	141.9
GmPP2C059	Glyma.09G280700	E	2.90E-41	144.1
GmPP2C060	Glyma.10G009800	G	4.70E-66	225.3
GmPP2C061	Glyma.10G047500	D	1.60E-39	138.4
GmPP2C062	Glyma.10G149600	H	1.80E-33	118.6
GmPP2C063	Glyma.10G150100	E	3.20E-41	143.9
GmPP2C064	Glyma.10G259400	D	6.50E-39	136.4

GmPP2C065	Glyma.10G271500	H	1.00E-35	125.9
GmPP2C066	Glyma.10G282900	D	5.30E-39	136.7
GmPP2C067	Glyma.10G291800	F	1.90E-63	216.8
GmPP2C068	Glyma.10G294400	D	1.20E-40	142.1
GmPP2C069	Glyma.10G297900	E	1.20E-32	115.8
GmPP2C070	Glyma.11G004000	No group	1.10E-46	161.8
GmPP2C071	Glyma.11G018000	A	1.70E-60	207.1
GmPP2C072	Glyma.11G050900	D	9.40E-37	129.3
GmPP2C073	Glyma.11G087000	G	2.10E-68	233.1
GmPP2C074	Glyma.11G139500	C	1.00E-29	106.3
GmPP2C075	Glyma.11G170200	B	5.30E-60	205.5
GmPP2C076	Glyma.11G171600	B	5.30E-60	205.5
GmPP2C077	Glyma.11G222600	A	7.50E-65	221.4
GmPP2C078	Glyma.12G109500	E	1.40E-38	135.3
GmPP2C079	Glyma.12G116800	F	1.30E-57	197.7
GmPP2C080	Glyma.12G202400	E	1.20E-37	132.3
GmPP2C081	Glyma.12G226100	F	5.50E-39	136.6
GmPP2C082	Glyma.13G042100	F	2.30E-65	223.1
GmPP2C083	Glyma.13G106800	A	2.30E-66	226.3
GmPP2C084	Glyma.13G135300	D	2.40E-38	134.5
GmPP2C085	Glyma.13G165300	G	3.50E-68	232.3
GmPP2C086	Glyma.13G211800	J	1.30E-37	132.2

GmPP2C087	Glyma.13G274800	F	3.40E-53	183.2
GmPP2C088	Glyma.13G299300	E	3.00E-38	134.2
GmPP2C089	Glyma.14G066400	A	2.10E-65	223.2
GmPP2C090	Glyma.14G082200	H	9.20E-29	103.1
GmPP2C091	Glyma.14G101400	I	2.10E-70	239.6
GmPP2C092	Glyma.14G104900	F	3.70E-67	228.9
GmPP2C093	Glyma.14G108400	A	2.40E-67	229.6
GmPP2C094	Glyma.14G161400	F	4.70E-65	222.1
GmPP2C095	Glyma.14G162100	A	4.70E-60	205.7
GmPP2C096	Glyma.14G195200	B	4.50E-62	212.3
GmPP2C097	Glyma.15G053700	F	4.20E-53	182.9
GmPP2C098	Glyma.15G100900	J	9.40E-38	132.6
GmPP2C099	Glyma.15G139500	D	1.80E-34	121.8
GmPP2C100	Glyma.15G172500	A	9.50E-69	234.2
GmPP2C101	Glyma.15G201300	G	4.90E-67	228.5
GmPP2C102	Glyma.16G122200	D	5.80E-39	136.6
GmPP2C103	Glyma.17G020400	J	1.40E-39	138.5
GmPP2C104	Glyma.17G026100	C	1.10E-31	112.7
GmPP2C105	Glyma.17G029200	E	3.20E-41	143.9
GmPP2C106	Glyma.17G034500	D	6.80E-36	126.5
GmPP2C107	Glyma.17G038000	G	1.20E-62	214.1
GmPP2C108	Glyma.17G052500	A	2.30E-66	226.4

GmPP2C109	Glyma.17G105800	G	1.10E-66	227.4
GmPP2C110	Glyma.17G218700	A	1.20E-66	227.2
GmPP2C111	Glyma.17G221100	F	3.70E-67	228.9
GmPP2C112	Glyma.17G223800	I	9.60E-72	244
GmPP2C113	Glyma.17G230600	E	4.90E-41	143.3
GmPP2C114	Glyma.17G243100	H	3.80E-28	101.1
GmPP2C115	Glyma.18G035000	A	4.30E-63	215.6
GmPP2C116	Glyma.18G061000	B	1.60E-60	207.2
GmPP2C117	Glyma.18G180400	C	8.80E-33	116.3
GmPP2C118	Glyma.18G208100	E	1.10E-40	142.2
GmPP2C119	Glyma.18G242700	E	1.50E-41	145
GmPP2C120	Glyma.18G283300	E	1.60E-41	144.9
GmPP2C121	Glyma.19G069200	A	3.80E-61	209.3
GmPP2C122	Glyma.19G147800	D	1.10E-38	135.6
GmPP2C123	Glyma.19G176600	D	2.60E-37	131.1
GmPP2C124	Glyma.19G230200	H	5.20E-34	120.3
GmPP2C125	Glyma.19G230600	E	1.60E-39	138.4
GmPP2C126	Glyma.20G106800	D	5.30E-39	136.7
GmPP2C127	Glyma.20G119100	H	6.30E-35	123.3
GmPP2C128	Glyma.20G131500	D	1.30E-39	138.7
GmPP2C129	Glyma.20G238100	E	9.10E-40	139.2
GmPP2C130	Glyma.20G238700	H	1.50E-32	115.5

GmPP2C131	Glyma.20G241100	F	5.90E-46	159.5
GmPP2C132	Glyma.20G244300	D	2.80E-41	144.1
GmPP2C133	Glyma.20G248900	E	8.10E-45	155.7
GmPP2C134	Glyma.U025900	F	1.80E-52	180.8
