# Supplementary material for: Pervasive duplication, biased molecular evolution and comprehensive functional analysis of the PP2C family in Glycine max
Source: BMC Genomics. 2020 Jul 6;21:465. doi: 10.1186/s12864-020-06877-4 (PMC7339511; doi:10.1186/s12864-020-06877-4)
Supplement: Supplementary file 25 [file 12864_2020_6877_MOESM3_ESM.docx]

**Additional file 3.** VvPP2C proteins identified in *V. vinifera.*

| Name | Locus name | Subfamily | E-value | Score |
|---|---|---|---|---|
| VvPP2C01 | GSVIVT01000159001 | D | 2.70E-38 | 132.6 |
| VvPP2C02 | GSVIVT01000543001 | B | 8.70E-58 | 196.5 |
| VvPP2C03 | GSVIVT01001129001 | C | 1.20E-33 | 117.4 |
| VvPP2C04 | GSVIVT01003115001 | No group | 9.30E-51 | 173.5 |
| VvPP2C05 | GSVIVT01003117001 | No group | 2.40E-44 | 152.5 |
| VvPP2C06 | GSVIVT01004849001 | F | 8.40E-55 | 186.7 |
| VvPP2C07 | GSVIVT01006890001 | D | 4.30E-32 | 112.3 |
| VvPP2C08 | GSVIVT01008355001 | No group | 2.40E-57 | 195.1 |
| VvPP2C09 | GSVIVT01009035001 | F | 6.40E-55 | 187.1 |
| VvPP2C10 | GSVIVT01009374001 | D | 4.20E-40 | 138.6 |
| VvPP2C11 | GSVIVT01009518001 | H | 2.20E-30 | 106.7 |
| VvPP2C12 | GSVIVT01009984001 | F | 2.80E-63 | 214.5 |
| VvPP2C13 | GSVIVT01011853001 | H | 5.10E-33 | 115.3 |
| VvPP2C14 | GSVIVT01012879001 | G | 4.90E-66 | 223.5 |
| VvPP2C15 | GSVIVT01013399001 | G | 6.10E-72 | 242.9 |
| VvPP2C16 | GSVIVT01014301001 | F | 2.90E-55 | 188.2 |
| VvPP2C17 | GSVIVT01014860001 | E | 3.60E-41 | 142.1 |
| VvPP2C18 | GSVIVT01015156001 | A | 3.10E-65 | 220.9 |
| VvPP2C19 | GSVIVT01015308001 | A | 6.00E-62 | 210.1 |
| VvPP2C20 | GSVIVT01015338001 | J | 4.30E-36 | 125.4 |

| | | | | |
|---|---|---|---|---|
| VvPP2C21 | GSVIVT01016046001 | I | 1.00E-72 | 245.5 |
| VvPP2C22 | GSVIVT01016485001 | A | 2.30E-61 | 208.2 |
| VvPP2C23 | GSVIVT01016816001 | A | 1.30E-65 | 222.1 |
| VvPP2C24 | GSVIVT01016988001 | A | 2.90E-56 | 191.5 |
| VvPP2C25 | GSVIVT01016995001 | A | 2.30E-56 | 191.9 |
| VvPP2C26 | GSVIVT01017639001 | G | 2.00E-60 | 205.2 |
| VvPP2C27 | GSVIVT01017754001 | C | 7.30E-32 | 111.6 |
| VvPP2C28 | GSVIVT01017919001 | No group | 1.70E-44 | 152.9 |
| VvPP2C29 | GSVIVT01018464001 | A | 9.00E-57 | 193.2 |
| VvPP2C30 | GSVIVT01019076001 | E | 1.70E-42 | 146.4 |
| VvPP2C31 | GSVIVT01019525001 | A | 1.80E-63 | 215.1 |
| VvPP2C32 | GSVIVT01019544001 | A | 4.30E-52 | 177.8 |
| VvPP2C33 | GSVIVT01020581001 | F | 3.70E-57 | 194.5 |
| VvPP2C34 | GSVIVT01022296001 | G | 2.70E-71 | 240.8 |
| VvPP2C35 | GSVIVT01023994001 | D | 1.10E-37 | 130.6 |
| VvPP2C36 | GSVIVT01024710001 | B | 3.70E-58 | 197.7 |
| VvPP2C37 | GSVIVT01024875001 | A | 1.80E-66 | 224.9 |
| VvPP2C38 | GSVIVT01024976001 | C | 5.40E-31 | 108.7 |
| VvPP2C39 | GSVIVT01025572001 | D | 4.00E-38 | 132.1 |
| VvPP2C40 | GSVIVT01025802001 | No group | 2.30E-65 | 221.4 |
| VvPP2C41 | GSVIVT01028192001 | G | 1.70E-65 | 221.8 |
| VvPP2C42 | GSVIVT01028698001 | A | 8.20E-47 | 160.5 |

| | | | | |
|---|---|---|---|---|
| VvPP2C43 | GSVIVT01028710001 | F | 1.00E-64 | 219.2 |
| VvPP2C44 | GSVIVT01030261001 | E | 8.20E-41 | 140.9 |
| VvPP2C45 | GSVIVT01030553001 | E | 2.40E-38 | 132.8 |
| VvPP2C46 | GSVIVT01031155001 | E | 3.10E-41 | 142.3 |
| VvPP2C47 | GSVIVT01031195001 | H | 1.40E-32 | 113.9 |
| VvPP2C48 | GSVIVT01032637001 | D | 2.90E-39 | 135.8 |
| VvPP2C49 | GSVIVT01032793001 | B | 2.70E-59 | 201.5 |
| VvPP2C50 | GSVIVT01033496001 | D | 7.80E-39 | 134.4 |
| VvPP2C51 | GSVIVT01034268001 | F | 1.00E-56 | 193 |
| VvPP2C52 | GSVIVT01034979001 | D | 2.40E-37 | 129.5 |
| VvPP2C53 | GSVIVT01035094001 | E | 1.40E-42 | 146.6 |
| VvPP2C54 | GSVIVT01035345001 | E | 4.50E-44 | 151.6 |
| VvPP2C55 | GSVIVT01035420001 | A | 2.80E-67 | 227.6 |
| VvPP2C56 | GSVIVT01035778001 | G | 6.00E-70 | 236.4 |
| VvPP2C57 | GSVIVT01035977001 | I | 6.80E-73 | 246 |
| VvPP2C58 | GSVIVT01036034001 | F | 8.70E-68 | 229.3 |
| VvPP2C59 | GSVIVT01036289001 | B | 4.30E-56 | 191 |
| VvPP2C60 | GSVIVT01037875001 | D | 1.50E-39 | 136.7 |
| VvPP2C61 | GSVIVT01038572001 | No group | 1.10E-52 | 179.8 |
| VvPP2C62 | GSVIVT01038792001 | F | 2.10E-58 | 198.5 |
