# Supplementary material for: Pervasive duplication, biased molecular evolution and comprehensive functional analysis of the PP2C family in Glycine max
Source: BMC Genomics. 2020 Jul 6;21:465. doi: 10.1186/s12864-020-06877-4 (PMC7339511; doi:10.1186/s12864-020-06877-4)
Supplement: Supplementary file 26 [file 12864_2020_6877_MOESM4_ESM.docx]

Additional file 4. AtPP2C proteins identified in *A. thaliana*

Name	Subfamily	E-value	Score
AT1G03590	E	1.20E-43	150.6
AT1G07160	B	4.40E-59	201.2
AT1G07430	A	8.70E-69	233
AT1G07630	C	8.00E-32	111.9
AT1G09160	H	1.90E-34	120.5
AT1G16220	E	6.00E-40	138.5
AT1G17550	A	6.10E-63	213.8
AT1G18030	No group	9.00E-56	190.3
AT1G22280	F	3.70E-53	181.8
AT1G34750	F	1.00E-52	180.3
AT1G43900	F	3.20E-62	211.5
AT1G47380	H	6.70E-29	102.3
AT1G48040	G	2.40E-63	215.2
AT1G67820	B	2.20E-60	205.4
AT1G68410	H	1.90E-37	130.3
AT1G72770	A	1.60E-62	212.4
AT1G78200	F	2.90E-57	195.2
AT1G79630	E	2.50E-42	146.3
AT2G20050	J	2.20E-35	123.5
AT2G20630	F	1.10E-53	183.5

AT2G25070	I	8.10E-75	252.7
AT2G25620	G	2.50E-69	234.8
AT2G28890	C	1.90E-30	107.4
AT2G29380	A	4.40E-66	224.1
AT2G30020	B	1.10E-57	196.7
AT2G33700	G	9.80E-79	265.6
AT2G34740	F	7.40E-54	184
AT2G40180	B	1.10E-55	190.1
AT2G40860	No group	2.50E-71	241.3
AT3G02750	E	3.70E-42	145.7
AT3G05640	E	3.00E-42	146
AT3G06270	J	4.00E-42	145.6
AT3G09400	C	1.50E-30	107.7
AT3G11410	A	9.80E-67	226.2
AT3G12620	D	4.80E-42	145.4
AT3G15260	F	7.40E-55	187.3
AT3G16560	C	1.00E-37	131.2
AT3G16800	E	2.50E-43	149.6
AT3G17090	D	4.40E-38	132.4
AT3G17250	G	1.80E-60	205.7
AT3G51370	D	8.40E-39	134.7
AT3G51470	G	4.30E-70	237.3

AT3G55050	D	8.60E-40	137.9
AT3G62260	G	1.90E-68	231.8
AT3G63320	No group	6.20E-39	135.2
AT3G63340	No group	1.30E-40	140.6
AT4G03415	E	8.90E-42	144.5
AT4G26080	A	1.10E-73	249
AT4G27800	No group	9.90E-56	190.2
AT4G28400	F	9.80E-57	193.5
AT4G31750	F	1.20E-66	225.9
AT4G31860	I	4.30E-73	247.1
AT4G32950	E	1.80E-44	153.3
AT4G33920	D	3.60E-39	135.9
AT4G38520	D	2.20E-43	149.7
AT5G01700	E	2.30E-36	126.7
AT5G02400	C	3.70E-30	106.4
AT5G02760	D	1.50E-41	143.8
AT5G06750	D	1.10E-37	131.1
AT5G10740	F	4.10E-65	220.9
AT5G19280	No group	4.20E-57	194.7
AT5G24940	F	4.80E-66	224
AT5G26010	E	7.90E-46	157.7
AT5G27930	E	3.60E-42	145.7

AT5G36250	E	1.80E-35	123.8
AT5G51760	A	1.50E-64	219.1
AT5G53140	F	9.70E-65	219.7
AT5G57050	A	1.50E-73	248.6
AT5G59220	A	7.10E-63	213.6
AT5G66080	D	1.80E-38	133.6
