# Supplementary material for: Pervasive duplication, biased molecular evolution and comprehensive functional analysis of the PP2C family in Glycine max
Source: BMC Genomics. 2020 Jul 6;21:465. doi: 10.1186/s12864-020-06877-4 (PMC7339511; doi:10.1186/s12864-020-06877-4)
Supplement: Supplementary file 27 [file 12864_2020_6877_MOESM5_ESM.docx]

Additional file 5. AtrPP2C proteins identified in *A. trichopoda*.

Name	Locus name	Subfamily	E-value	Score
AtrPP2C01	evm_27.model.AmTr_v1.0_scaffold00001.113	D	1.3E-40	140.3
AtrPP2C02	evm_27.model.AmTr_v1.0_scaffold00002.591	No group	4.4E-43	148.3
AtrPP2C03	evm_27.model.AmTr_v1.0_scaffold00003.176	G	4.7E-58	197.4
AtrPP2C04	evm_27.model.AmTr_v1.0_scaffold00003.63	D	4.3E-39	135.3
AtrPP2C05	evm_27.model.AmTr_v1.0_scaffold00007.121	B	1E-49	170.1
AtrPP2C06	evm_27.model.AmTr_v1.0_scaffold00009.126	A	2.2E-57	195.2
AtrPP2C07	evm_27.model.AmTr_v1.0_scaffold00012.311	A	2.1E-62	211.6
AtrPP2C08	evm_27.model.AmTr_v1.0_scaffold00016.308	C	2.6E-33	116.3
AtrPP2C09	evm_27.model.AmTr_v1.0_scaffold00017.291	F	5.7E-63	213.5
AtrPP2C10	evm_27.model.AmTr_v1.0_scaffold00019.384	F	2.8E-67	227.6
AtrPP2C11	evm_27.model.AmTr_v1.0_scaffold00023.135	D	3.9E-42	145.2
AtrPP2C12	evm_27.model.AmTr_v1.0_scaffold00023.47	E	1.2E-41	143.7
AtrPP2C13	evm_27.model.AmTr_v1.0_scaffold00025.147	J	1E-39	137.4
AtrPP2C14	evm_27.model.AmTr_v1.0_scaffold00025.340	F	4.7E-56	190.9
AtrPP2C15	evm_27.model.AmTr_v1.0_scaffold00025.395	No group	1.3E-41	143.6
AtrPP2C16	evm_27.model.AmTr_v1.0_scaffold00036.116	E	9.7E-47	160.3
AtrPP2C17	evm_27.model.AmTr_v1.0_scaffold00038.131	F	3.7E-55	187.9
AtrPP2C18	evm_27.model.AmTr_v1.0_scaffold00039.225	A	3.2E-62	211.1
AtrPP2C19	evm_27.model.AmTr_v1.0_scaffold00044.135	G	2E-70	238
AtrPP2C20	evm_27.model.AmTr_v1.0_scaffold00044.247	No group	7.1E-55	187

AtrPP2C21	evm_27.model.AmTr_v1.0_scaffold00044.67	D	6.5E-39	134.7
AtrPP2C22	evm_27.model.AmTr_v1.0_scaffold00045.253	C	6.2E-30	105.3
AtrPP2C23	evm_27.model.AmTr_v1.0_scaffold00051.41	No group	2.9E-65	221
AtrPP2C24	evm_27.model.AmTr_v1.0_scaffold00056.98	A	1.5E-57	195.8
AtrPP2C25	evm_27.model.AmTr_v1.0_scaffold00057.71	H	1.2E-36	127.3
AtrPP2C26	evm_27.model.AmTr_v1.0_scaffold00057.96	E	3.9E-40	138.7
AtrPP2C27	evm_27.model.AmTr_v1.0_scaffold00058.124	No group	2E-51	175.7
AtrPP2C28	evm_27.model.AmTr_v1.0_scaffold00061.19	E	1E-43	150.4
AtrPP2C29	evm_27.model.AmTr_v1.0_scaffold00065.64	B	2.6E-57	195
AtrPP2C30	evm_27.model.AmTr_v1.0_scaffold00067.67	F	1.9E-35	123.3
AtrPP2C31	evm_27.model.AmTr_v1.0_scaffold00078.152	B	5.8E-54	184
AtrPP2C32	evm_27.model.AmTr_v1.0_scaffold00079.109	H	5.7E-36	125
AtrPP2C33	evm_27.model.AmTr_v1.0_scaffold00086.102	F	1.2E-64	219
AtrPP2C34	evm_27.model.AmTr_v1.0_scaffold00099.46	B	2.5E-57	195.1
AtrPP2C35	evm_27.model.AmTr_v1.0_scaffold00109.155	J	1.9E-35	123.3
AtrPP2C36	evm_27.model.AmTr_v1.0_scaffold00122.48	G	6.6E-64	216.6
AtrPP2C37	evm_27.model.AmTr_v1.0_scaffold00126.48	H	7.8E-33	114.8
AtrPP2C38	evm_27.model.AmTr_v1.0_scaffold00137.1	I	7.6E-73	245.9
AtrPP2C39	evm_27.model.AmTr_v1.0_scaffold00146.58	A	2.7E-67	227.7
AtrPP2C40	evm_27.model.AmTr_v1.0_scaffold00163.14	E	3.8E-43	148.6
AtrPP2C41	evm_27.model.AmTr_v1.0_scaffold00183.21	F	1.8E-56	192.2
AtrPP2C42	evm_27.model.AmTr_v1.0_scaffold00309.1	F	4.3E-65	220.5

AtrPP2C43	evm_27.model.AmTr_v1.0_scaffold02147.1	G	5.1E-46	158
