# Supplementary material for: Pervasive duplication, biased molecular evolution and comprehensive functional analysis of the PP2C family in Glycine max
Source: BMC Genomics. 2020 Jul 6;21:465. doi: 10.1186/s12864-020-06877-4 (PMC7339511; doi:10.1186/s12864-020-06877-4)
Supplement: Supplementary file 28 [file 12864_2020_6877_MOESM6_ESM.docx]

Additional file 6. NcPP2C proteins identified in *N. colorata*.

Name	Locus name	Subfamily	E-value	Score
NcPP2C01	GWHPAAYW000011	F	1.1E-58	199.8
NcPP2C02	GWHPAAYW000224	B	5.2E-51	174.6
NcPP2C03	GWHPAAYW001233	A	1.8E-62	212.1
NcPP2C04	GWHPAAYW001792	No group	3.7E-33	116.1
NcPP2C05	GWHPAAYW001948	F	1.9E-54	185.8
NcPP2C06	GWHPAAYW002299	J	1.1E-33	117.8
NcPP2C07	GWHPAAYW003097	No group	1.6E-42	146.8
NcPP2C08	GWHPAAYW003248	F	3E-64	217.9
NcPP2C09	GWHPAAYW005484	D	8.4E-35	121.4
NcPP2C10	GWHPAAYW005594	E	3.3E-40	139.2
NcPP2C11	GWHPAAYW006104	F	6.4E-54	184.1
NcPP2C12	GWHPAAYW006890	H	3.7E-32	112.8
NcPP2C13	GWHPAAYW007004	No group	5.7E-69	233.4
NcPP2C14	GWHPAAYW009292	D	2.8E-43	149.2
NcPP2C15	GWHPAAYW009731	F	6E-54	184.2
NcPP2C16	GWHPAAYW012563	D	2.8E-40	139.4
NcPP2C17	GWHPAAYW012887	D	7.2E-38	131.5
NcPP2C18	GWHPAAYW012903	G	2.9E-69	234.4
NcPP2C19	GWHPAAYW013134	C	9.7E-34	118
NcPP2C20	GWHPAAYW013921	D	4.2E-41	142.1

NcPP2C21	GWHPAAYW014619	E	3.2E-40	139.2
NcPP2C22	GWHPAAYW014647	B	3.7E-50	171.8
NcPP2C23	GWHPAAYW015331	F	1.5E-63	215.6
NcPP2C24	GWHPAAYW015785	F	8.4E-61	206.6
NcPP2C25	GWHPAAYW016370	I	2.1E-57	195.5
NcPP2C26	GWHPAAYW019311	D	1E-39	137.6
NcPP2C27	GWHPAAYW019610	E	2E-42	146.4
NcPP2C28	GWHPAAYW021159	A	3.7E-55	188.1
NcPP2C29	GWHPAAYW021161	A	1.8E-55	189.2
NcPP2C30	GWHPAAYW022075	A	1.8E-60	205.5
NcPP2C31	GWHPAAYW023063	B	1.6E-56	192.6
NcPP2C32	GWHPAAYW025444	H	4.2E-29	102.8
NcPP2C33	GWHPAAYW025577	F	3E-51	175.3
NcPP2C34	GWHPAAYW026531	F	1.5E-46	160
NcPP2C35	GWHPAAYW027157	D	2.7E-40	139.5
NcPP2C36	GWHPAAYW027193	G	9.6E-71	239.2
NcPP2C37	GWHPAAYW027407	No group	8.3E-41	141.1
NcPP2C38	GWHPAAYW027725	J	4.5E-38	132.2
NcPP2C39	GWHPAAYW028183	D	4.8E-39	135.4
NcPP2C40	GWHPAAYW029188	I	1.8E-40	140
