# Supplementary material for: Pervasive duplication, biased molecular evolution and comprehensive functional analysis of the PP2C family in Glycine max
Source: BMC Genomics. 2020 Jul 6;21:465. doi: 10.1186/s12864-020-06877-4 (PMC7339511; doi:10.1186/s12864-020-06877-4)
Supplement: Supplementary file 29 [file 12864_2020_6877_MOESM7_ESM.docx]

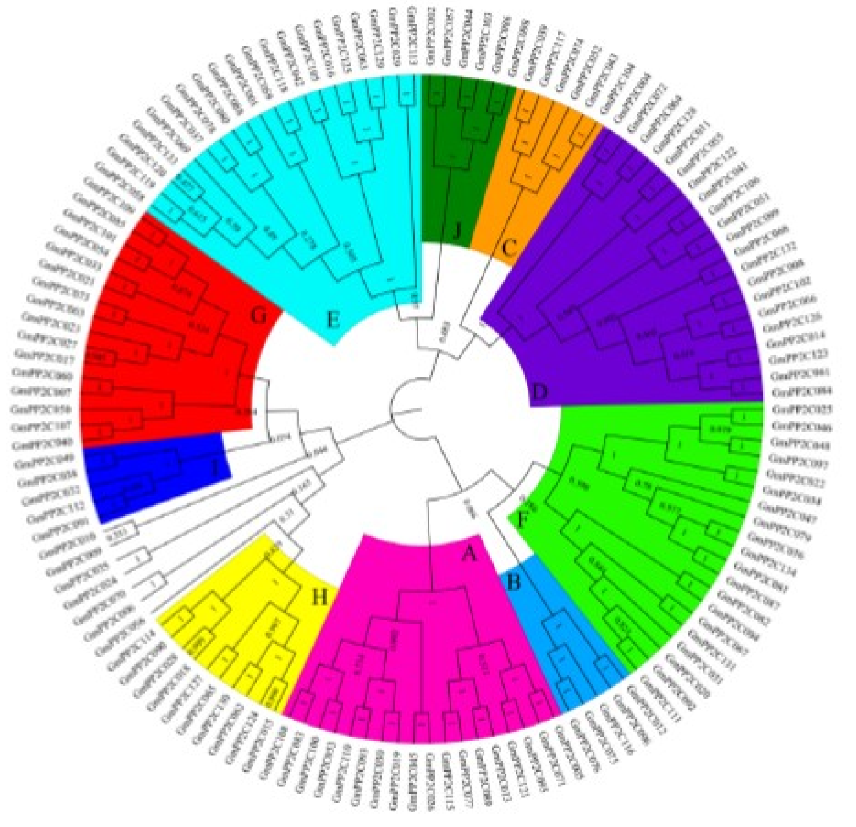

Additional file 7. Phylogenetic relationship of the GmPP2C members. The neighbor-joining (NJ) tree was constructed through the MEGA program, and the numbers in the clades are the bootstrap values. The PP2C subfamilies are grouped by colors.
